# Dissecting the genotypic features of a fluoroquinolone-resistant *Pseudomonas aeruginosa* ST316 sublineage causing ear infections in Shanghai, China

**DOI:** 10.1099/mgen.0.000989

**Published:** 2023-04-20

**Authors:** Zhewei Sun, Feifei Yang, Jian Ji, Wenjun Cao, Chunhong Liu, Baixing Ding, Xiaogang Xu

**Affiliations:** ^1^​ Shanghai Institute of Infectious Disease and Biosecurity, Fudan University, Shanghai, PR China; ^2^​ Institute of Antibiotics, Huashan Hospital, Fudan University, Shanghai, PR China; ^3^​ Key Laboratory of Clinical Pharmacology of Antibiotics, Ministry of Health, Shanghai, PR China; ^4^​ Department of Clinical Laboratory, Eye and ENT Hospital, Fudan University, Shanghai, PR China

**Keywords:** *Pseudomonas aeruginosa*, sequence type 316, microbial evolution, machine learning, comparative genomics

## Abstract

Limited information is available regarding the genomic characteristics of *P. aeruginosa* causing ear infections. Our aim is to characterize the genotypic features of an emerging ST316 sublineage causing aural infections in Shanghai. A total of 199 ear swab isolates were subjected to whole genome sequencing (WGS). Complete genomes for two isolates were resolved. We showed this recently emerged sublineage exhibited high-level resistance to fluoroquinolones (FQs) primarily by accumulation of known mutations in quinolone resistance determining regions (QRDRs). Loss-of-function mutations in *mexR* and *mexCD* were frequently detected. Mutations in *fusA1* (P166S) and *parE* (S492F) were resident in this sublinage about 2 years after its emergence. Recombination events might be a key driver of genomic diversity in this sublineage. Convergent evolution events on Multidrug-resistant (MDR) determinants were also observed. We generated predictive machine models and identified biomarkers of resistance to gentamicin, fosfomycin, and cefoperazone-sulbactam in this sublineage. This sublineage tended to be less virulent by loss of a series virulence genes represented by *ppkA, rhlI*, and iron uptake- and antimicrobial activity-related genes. Specific mutations were detected in *pilU* and *lpxB* genes that related to surface structures. Moreover, this sublineage differed from non-ST316 isolates in several ways, including virulence genes related to cell surface structure. Our analysis suggested acquisition of a roughly 390 kbp MDR plasmid carrying *qnrVC1* might play an important role in the success of this sublinage. Clonal expansion of this sublineage exhibiting enhanced adaptation to cause ear infections is concerning, which requires urgent control measures to be implemented.

## Data Summary

One hundred and ninety-nine *P. aeruginosa* genomes generated in this study are available in the Sequence Read Archive (SRA) and GenBank databases under BioProject accession PRJNA793523.

**Impact Statement**

*
P. aeruginosa* is one of the leading species causing aural infections. Despite the intense interest in *P. aeruginosa* aural infections, little is known about the genetic features of *P. aeruginosa* isolated from ear canal. In this study, we showed that a relatively uncommon clonal type, ST316, caused the plurality of multidrug-resistant *P. aeruginosa* aural infections in our patients. We performed the first large scale genome-wide analysis of *P. aeruginosa* ear swab isolates to characterize their potential genetic requirement for conferring an advantage in adapting during ear infections. Our results may portend the emergence of an especially successful clonal group of antibiotic-resistant *P. aeruginosa* causing aural infections.

## Introduction

*Pseudomonas aeruginosa* is an opportunistic pathogen that causes a wide range of syndromes in humans, which can vary from local to systemic, subacute to chronic and superficial, and self-limiting to life-threatening [1]. Due to the prevalence of MDR *P. aeruginosa* and β-lactamase producing *Staphylococcus aureus* since the last few decades, aural infections are becoming increasingly difficult to treat [2]. Being one of the leading species causing aural infections, *P. aeruginosa* is isolated from aural drainage in more than 90 % of cases of malignant external otitis [3]. Aural infections caused by *P. aeruginosa* can lead to hearing loss and life-threatening central nervous system complications, including brain abscess and meningitis [4]. Despite the emerging burden of *P. aeruginosa* aural infections on the public health, little is known regarding its infection-causing genetic features.

*P. aeruginosa* can develop resistance and virulence by accumulating genomic mutations and exchanging transferable resistance or virulence determinants [5]. As such, the ecological versatility of *P. aeruginosa* is associated with its relatively large genome containing numerous regulatory genes and plasticity regions that confer an adaptive advantage during prolonged infections [6]. Three major antibiotic families are available to treat *P. aeruginosa* infections namely fluoroquinolones, β-lactams (including carbapenems), and aminoglycosides [7]. Especially, FQs are the first-line drug to treat ear infections, thus, emergence of FQ-resistant clone type causing aural infections is the most concerning.

Our previous work has investigated the antimicrobial resistance (AMR), and biofilm formation phenotype of *P. aeruginosa* ear swab isolates (*n*=199) [8]. A preliminary multi-locus sequence typing analysis revealed that 147 (73.87 %) of them belonged to a relatively uncommon clonal type, ST316. Here, using WGS, we reaffirmed that 147 of the 199 *P *. *aeruginosa* ear swab isolates belonged to a ST316 sublineage. We additionally included 45 globally sourced ST316 isolates to characterize the evolutionary history, epidemiology, genotypic features, and adaptive signatures of this sublineage (Table S1). Comparative genomic analysis of the 199 *P *. *aeruginosa* ear swab isolates was performed to identify potential ear infection causing signatures in this sublineage.

## Methods

### Bacterial isolates and antimicrobial susceptibility testing

One hundred and ninety-nine isolates were cultured from ear swab specimens of outpatients presented with aural inflammation between January 2019 and December 2020 at the Eye and ENT Hospital, Fudan University, Shanghai, China. Quality control and interpretation of the results were based on the Clinical and Laboratory Standards Institute (CLSI) 2021 breakpoints for all antimicrobial agents [9], except cefoperazone and cefoperazone/sulbactam for which CLSI criteria were not available. Therefore, the cefoperazone and cefoperazone/sulbactam MICs were estimated using the CLSI 2021 breakpoints for *Enterobacterales *.

### WGS and analysis

Whole-genome shotgun sequencing was performed on an Illumina novaseq 6000 platform (Illumina, San Diego, CA, USA). After adaptor trimming and quality filtering using Trimmomatic v0.27 [10], the processed reads were assembled *de novo* using ABySS v2.3.3 [11] and SPAdes v3.14.1 [12] software, and the better assembly was chosen according to N50. In addition, the genome of two ST316 ear swab isolates (HS_13 and HS_121) were sequenced on the PacBio RS II platform. Canu v 2.1 [13] was used to assemble the Illumina corrected long reads. Genes were predicted and annotated using Prokka v1.14.5 [14]. SNP calling was performed with Snippy v4.6.0 (https://github.com/tseemann/snippy) using HS_13 as the reference. The core SNP alignment was filtered for recombination using Gubbins v2.4.1 [15]. A maximum likelihood tree was inferred with RAxML v8.2.12 [16] using GTR+CAT (1000 bootstrap replicates) and was midpoint rooted in iTOLs (https://itol.embl.de/) for visualization with metadata. The time to the most recent common ancestor of the ED sublineage was estimated using BEAST v2.6.3 [17] on a multi-TypeTree template with a strict clock model and 5 000 000 Markov chain Monte Carlo iterations. Virulence genes were identified by examining the VFDB database (http://www.mgc.ac.cn/VFs/) using abricate (https://github.com/tseemann/abricate). To determine to presence of the 390 kb MDR plasmid in our collection, we first predicted the plasmid contigs using mlplasmids [18]. The predicted plasmid contigs were mapped to pHS13_391 (CP104566) using minimap2 [19]. Genomes were considered present on the 390 kb *qnrVC1*-bearing plasmid if they had ≥ 90 % mapping coverage. Core genes accumulated significantly more substitutions (FDR-adjusted *P*<0.05) than under the null hypothesis of neutral evolution (Poisson process) or possessed homoplastic SNPs (evolved independently at least three times) were defined as under positive selection. PAML [20] was used for ancestral state reconstruction of all SNPs. The k-mer counts were determined for each genome using unitig-counter (https://github.com/bacpop/unitig-counter). Machine learning analyses were performed on 147 ED ST316 isolates using the sci-kit learn library (https://www.jmlr.org/papers/v12/pedregosa11a.html) in Python v3.8. All possible combinations of hyperparameters were tested through nested ten-fold cross-validation. GWAS was performed using Scoary [21].

### Nucleotide data accessions

The sequence data of 147 ST316 and 52 non-ST316 isolates have been deposited in the in the NCBI GenBank database under BioProject accession no. PRJNA793523. Relevant sequencing data were deposited in the Sequence Read Archive under accession numbers SRR21536900 to SRR21537102. Complete genome sequences of HS_13 and HS_121 were submitted to NCBI GenBank with accession numbers: CP104565 to CP104566 and CP104567 to CP104568, respectively.

## Results

### The clonal expansion of *P. aeruginosa* ST316 ED sublineage

**Fig. 1.**
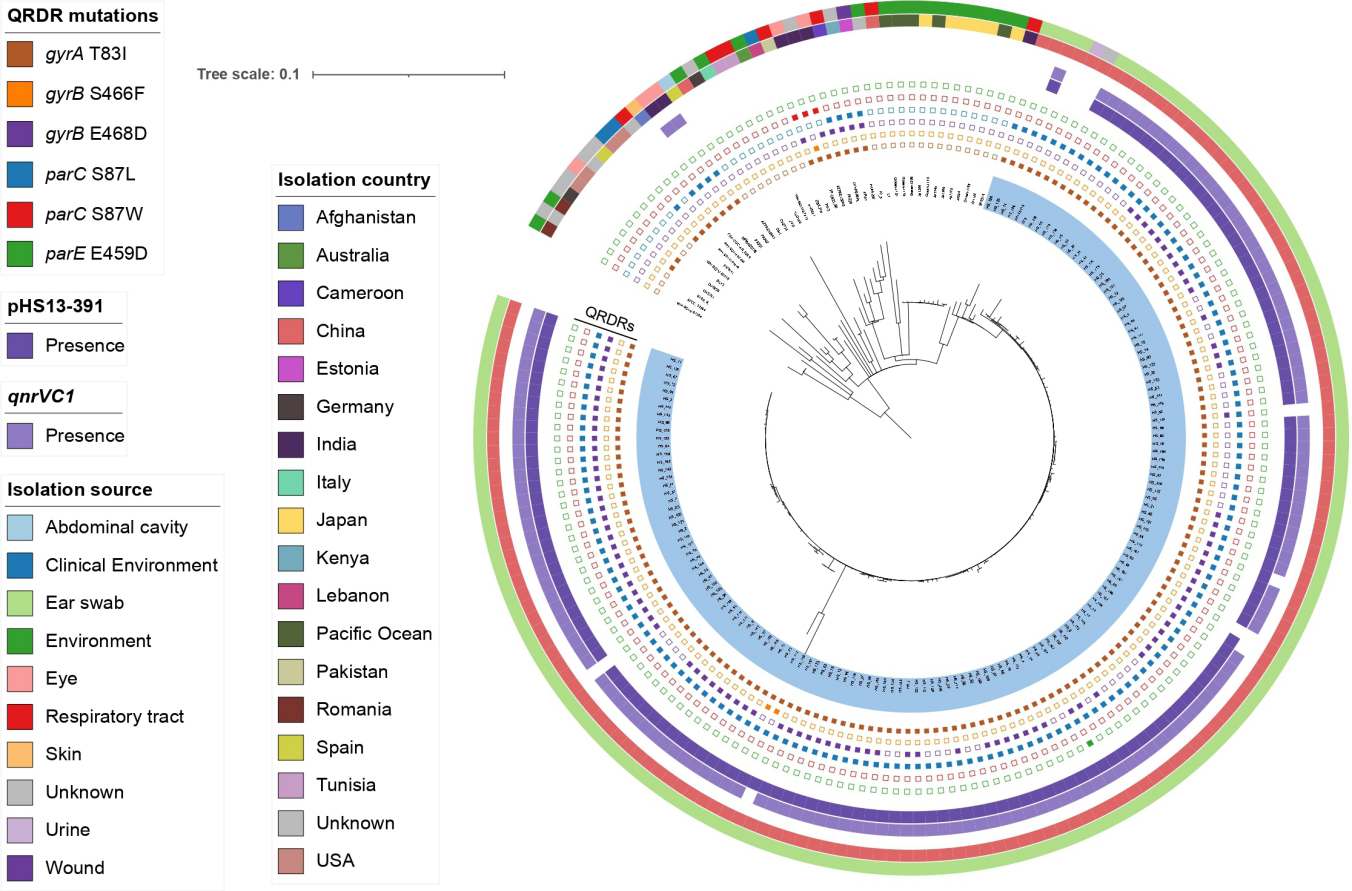
Maximum-likelihood non-recombinant core SNP-based phylogeny of *P. aeruginosa* ST316 lineage. The tree is rooted at the midpoint. Strain names of isolates belonging to the ED sublineage are coloured in light blue. Concentric rings 1 to 6 (from inside to outside) indicate the presence (closed symbol) of mutations in quinolone resistance determining region (QRDRs). Concentric rings 7 and 8 represent the presence of ~390 kbp MDR plasmid and *qnrVC1*, respectively. Concentric rings 9 and 10 represent geographical origin and isolation source, respectively.

Our previous work reported a predominance of *P. aeruginosa* ST316 strains among patients with ear infections in Shanghai, China [8]. All but two of these ST316 strains exhibited high-level resistance to FQs (Table 1). Here, we utilized a global collection of 192 *P *. *aeruginosa* ST316 isolates, including 147 contemporary ear discharge (ED) isolates sequenced for the purpose of this study. Phylogenetic analysis of the 192 isolates revealed that 149 of them form a monoclade (here named ST316 ED sublineage, 147 from our collection, the other two from Guangzhou, China) (Fig. 1), with paired SNP distances ranging from 0 to 151 (median, 5). Isolates from this sublineage differed by 7.44 SNPs on average, after excluding two possible hypermutators (HS_112 and HS_156) that contained many strain-specific SNPs (not caused by contamination). The ED sublineage was predicted to have emerged in 2012 (Fig. S1, available in the online version of this article), which suggested a recent clonal expansion event. Two classical mutations in the quinolone resistance determining region (QRDR): GyrA-T83I and ParC-S87L were fixed in this population, furthermore, 65.10 % strains (97/149) also exhibited the E468D change in GyrB. We observed a single Indian ST316 isolate (strain SP2514) that was a sister taxon to the ED sublineage exhibiting a wild-type QRDR sequences compatible with a full susceptibility to FQs.

**Table 1.** Antimicrobial susceptibility profiles of 199 ear swab isolates from our collection MIC=minimum inhibitory concentration.

		ST316 (*n*=147)				non-ST316 (*n*=52)		
Drug	MIC_50_	MIC_90_	MIC range		MIC_50_	MIC_90_	MIC range	Total Susceptibility Rate (%)
Gentamicin	32	128	0.125 to 128		2	128	1 to 128	88 (44.22)
Amikacin	4	8	0.25 to 32		4	8	1 to 128	195 (97.99)
Ciprofloxacin	>128	>128	8 to >128		0.25	128	0.0625 to 128	33 (16.58)
Levofloxacin	>128	>128	16 to >128		1	128	0.25 to 128	30 (15.08)
Imipenem	1	2	0.125 to 16		1	2	0.25 to 32	191 (95.98)
Meropenem	0.25	0.5	0.06 to 16		0.25	2	0.0625 to 128	190 (95.48)
Aztreonam	16	32	0.5 to 128		4	32	1.0 to 32	70 (35.18)
Piperacillin-tazobactam*	16	32	1 to 128		4	16	1.0 to 64	175 (87.94)
Piperacillin	16	32	2 to 64		4	32	1 to 128	161 (80.90)
Ceftazidime-avibactam†	4	8	0.25 to 8		1	4	0.25 to 8	199 (100.00)
Ceftazidime	4	8	0.5 to 64		2	4	0.5 to 16	196 (98.49)
Cefoperazone-sulbactam‡	16	32	2 to 64		4	32	1 to 128	123 (61.81)
Cefoperazone	32	32	4 to 128		4	32	1 to 128	116 (58.29)
Cefepime	4	8	0.5 to 64		2	8	0.5 to 16	182 (91.46)
Fosfomycin	64	128	8 to 128		64	128	8 to 128	156 (78.39)
Polymyxin B	1	1	0.125 to 8		1	2	0.25 to 2	197 (98.99)

*Tazobactam at fixed concentration of 4 mg l^–1^.

†Avibactam at a fixed concentration of 4 mg l^–1^.

‡Sulbactam at fixed concentration of 4 mg l^–1^. Data was taken from our previous work [8].

### Recombination events in the ED sublineage

Previous study has suggested that recombination was an important driver of genomic and phenotypic diversity in a *P. aeruginosa* during infection [22]. In this study, numerous recombination events were also identified across the ST316 lineage. The affected regions including four prophages, one genomic island, and one type IVa pilus (T4aP) pathogenicity island (PI) inserted in the 5′ end of tRNA^Thr^ (Fig. S2A). In particular, there was a high density of recombination events within the T4aP island. The overall ratios of base substitutions resulting from homologous recombination relative to point mutations (r/m) of the ED sublineage was estimated to be 4.01, suggesting recombination rather than spontaneous mutation played an important role in in generating diversity in the ED population. Strikingly, we observed a large recombination region (~ 375 kbp) that was specifically detected in a subset of ED isolates (*n*=25, here named ED* isolates) (Fig. S2A). In the HS_13 reference genome, this region carried virulence genes encoding GacA (a part of GacA/GacS two-component regulatory system), siderophore pyoverdine (*pvd* locus) and H3-type VI secretion system (H3-T6SS). Alignment of a representative genome (HS_121) of ED* to the HS_13 reference genome revealed that a prophage (closely related to phage YMC11/02 /R656, accession no. NC_028657) and a part of T4aP island had been excised from the ED* isolates (Fig. S2B). Additionally, ED* isolates independently acquired a phage JBD93 (NC_030918). Notably, *fiuA* (tonB-dependent ferrichrome receptor) was inactivated by an *IS*Pa62 element in all ED* isolates (Fig. S2B). Although these virulence factors linked to biofilm phenotype in *P. aeruginosa* [23–26], we did not observe significant differences in biofilm production between ED and ED* isolates (Fig. S3).

### Multi-drug resistance in the ED sublineage

One hundred and eighteen of the 147 ED isolates from our collection were non-susceptible to at least one agent in three or more antimicrobial categories and could be classified as MDR strains [27] (Fig. S4). Notably, three strains (HS_48, HS_54, and HS_186) could be further classified as extensively drug-resistant (XDR) strains due to the non-susceptibility to at least one agent in all but two or fewer antimicrobial categories. A total of 69 acquired AMR genes were identified in the ST316 lineage (Fig. S5). The number of AMR genes were significantly higher in the ED sublineage than in non-ED isolates (Fig. S6). Further investigation revealed that the increasing AMR genes in the ED sublineage were caused by a roughly 390 kbp MDR plasmid that was most stably maintained in this sublineage (95.30%, 142/149) (Fig. 1, Table 2). The MDR region on this plasmid consisted of two highly variable class I integrons, and carried 11 AMR genes (*qnrVC1*, *aadA11*, two *qacEΔ1*, two *sul1*, *floR*, *tet(G*), *tetA*, *arr-3*, *aac(6’)-Ib*) which could confer resistance to seven antimicrobial classes. Analysis of plasmid gene content revealed that except for *qnrVC1*, the other AMR genes were less conserved in this MDR region (Fig. S7). Insertion sequences (ISs) were often identified at the boundaries of or within the integron, suggesting transposition could play a role in the mosaic structure of this MDR region. A blastn search (pHS13-391 as a query) against a custom database of all published *qnrVC1*-bearing plasmids, revealed that all plasmids that shared high sequence identity (≥97.47 %) with ours originated from China, spanning seven provinces (Table S2), suggesting this plasmid has been prevalent across China.

**Table 2.** Distribution of antibiotic resistance genes between the ED sublineage and non-ED isolates Resistance genes leading to a *P* value<e-5 sorted by significance after performing a chi-square test or Fisher’s exact test between ED and non-ED isolates.

Gene	*P* value	ED sublineage (*n*=149)	non-ED isolates (*n*=43)
*qnrVC1*	3.82E-29	138 (92.62 %)	2 (4.65 %)
*arr-3*	8.01E-15	90 (60.40 %)	0
*floR*	1.31E-14	89 (59.73 %)	0
*aac(6')-Ib*	1.05E-11	89 (59.73 %)	2 (4.65 %)
*aph(3'')-Ib*	4.16E-09	0	12 (27.91 %)
*aph(6)-Id*	4.55E-08	1 (0.67 %)	12 (27.91 %)
*tet(G*)	2.02E-06	89 (59.73 %)	8 (18.6 %)
*floR2*	3.67E-06	0	8 (18.6 %)

It is well known that the aminoglycoside antibiotic gentamicin can cause both ototoxicity and nephrotoxicity [28], thus cannot be used to treat ear infection. However, the rate of resistance to gentamicin was very high in our ED isolates (91/147 were resistant, 10/147 were intermediate). The gentamicin resistance is highly associated with the presence of *aac(6’)-Ib* gene which was reported to confer resistance to gentamicin [29] (r=0.74, Kendall’s Tau correlation test). We hypothesized that gentamicin resistance in ED isolates could be induced by the frequent usage of neomycin ear drops in our patients.

**Fig. 2.**
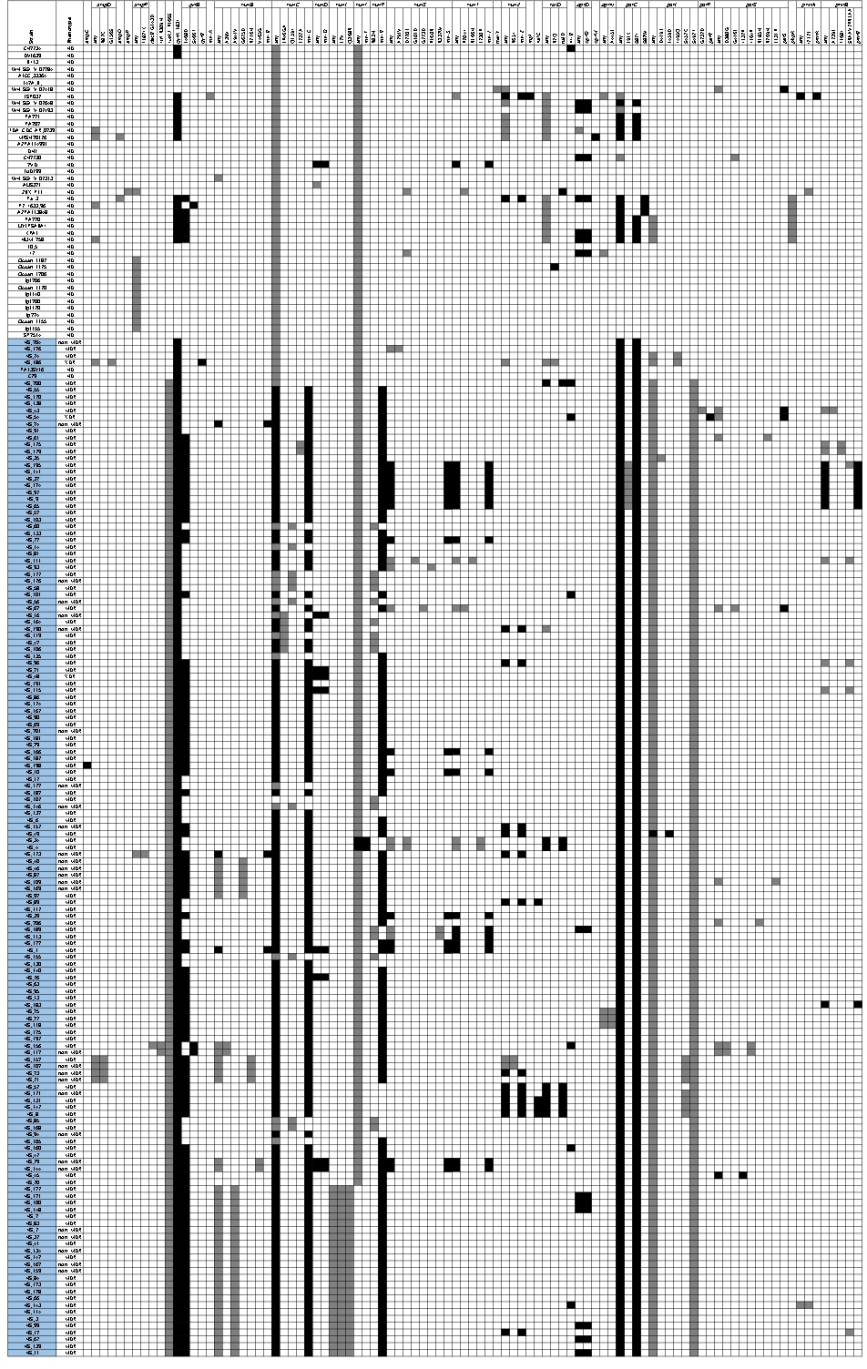
WGS mutational resistome analysis of 192 ST316 isolates used in this study. Forty chromosomal genes involved in mutational resistance (PAO1 as a reference) [46] were examined in this analysis. For AmpC regulators, reference (wild-type) gene sequence were taken from a previous study [31]. Colour codes: inactivating loss-of-function mutations and well-characterized gain-of-function mutations are indicated in black whereas any other amino acid substitutions are indicated in grey. Due to the sequence diversity of *mexR* and *oprD*, *mexR* from strain DN1 (ST316) and *oprD* from HS_13 were used as a reference. Mutations fixed in ST316 lineage are not shown. Strain names of isolates belonging to the ED sublineage are coloured in light blue.

Furthermore, we observed that many isolates from our collection exhibited intermediate resistance (susceptible, increased exposure) to antipseudomonal penicillins or cephalosporins (Fig. S4). The pre-resistance was concerning, which could increase the risk of becoming resistant in the future [30]. We did not detect any mutations in genes whose inactivation up-regulates AmpC cephalosporinase production that could be associated with the pre-resistant strains (Fig. 2) [31]. However, one hundred and twenty-two ED isolates exhibited inactivating mutations of *mexR* (76.51 %, 114/149), *nalC* (2.68 %, 4/149) or *nalD* (5.37 %, 8/149). Additionally, mutations in *mexS-mexT* and *mexZ* were observed in about a dozen of the ED isolates. Of note, *mexC* was inactivated in 126 (84.56 %) ED strains, which might lead to the compensatory changes in the expression level of the efflux pump MexAB-OprM [32]. In addition to classical mutations in QRDRs, we also observed a frequent substitution in codon 492 of ParE (S492F) in ED isolates (95.97 %, 143/149). Mutations in *fusA1* gene have been proposed to be involved in a novel mechanism of aminoglycoside resistance in *P. aeruginosa* [33]. Here, we showed that an unreported substitution (P166S) specifically affected 143 (95.97 %) ED isolates. The S492F and P166S change in ParE and FusA1*,* respectively, both first appeared in ~2014 in isolates HS_156 and HS_112 (data not shown).

Although we did not detect any carbapenemases in our collection (Fig. S5), seven of the ED isolates (HS_189, HS_11, HS_67, HS_99, HS_121, HS_100, and HS_148) showed resistance to imipenem/meropenem (Fig. S4). Mutational analysis suggested that the loss-of-function (LOF) mutations in outer membrane protein OprD could be responsible for the carbapenem-resistance in these strains. Furthermore, by searching for convergent evolution events, the independent evolution of mutations at the same nucleotide site or gene, we observed additional signatures (i.e. positively selected genes) that could contribute to the MDR phenotype in the ED sublineage. We showed that *mexR*, *mexB*, *mexC*, *parS*, and *parE* were under positive selection (Fig. S8). The ParS/ParR two component regulatory system played critical roles for MDR in *P. aeruginosa *, which could link to the *mexXY-OprM* efflux system, *arn* operon, and *oprD* porin.

### Prediction of resistance to antimicrobial agents based on whole-genome sequence data

**Fig. 3.**
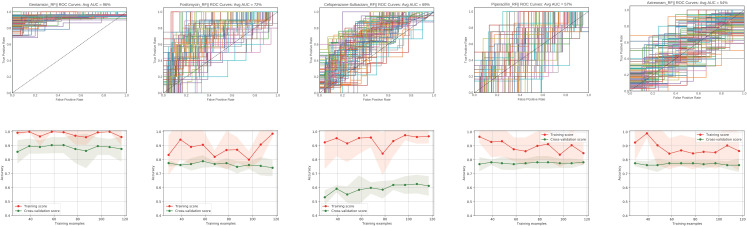
Performance of the random forest algorithm in predicting *P. aeruginosa* resistance based on k-mers. The average AUC was calculated by averaging over AUCs for the 100 independent random forest ROC curves with the best parameters determined by cross-validation. The y-axis is the true positive rate and the x-axis is the false positive rate. Learning curve (bottom) showing change in mean training accuracy (red line) and cross-validation accuracy (green line) in predicting *P. aeruginosa* resistance increasing numbers of isolates are used to train the random forest model. Shading indicates the 95 % confidence interval. Assessments at each number of training examples were through ten-fold nested cross-validation.

The phenotypic variation of antimicrobial resistance among isolates from the ED sublineage must reflect underlying genetic (or epigenetic) variation, even if the ED sublineage exhibited rapid clonal expansion. We used non-core k-mers of 147 ST316 ear swab genomes to predict susceptibility (S) and non-susceptibility (NS) phenotypes to aztreonam, cefoperazone-sulbactam, fosfomycin, gentamicin, and piperacillin (each antibiotic had at least 30 S or NS strains) (Fig. S9) with a random forest classifier. The machine learning approach here showed mean area under the curve (AUC) scores of 0.96, 0.72, 0.69, 0.57, and 0.54 for gentamicin, fosfomycin, cefoperazone-sulbactam, piperacillin, and aztreonam, respectively (Fig. 3). To assess whether the poor performance of the machine learning models for certain antibiotics (piperacillin and aztreonam) was the result of an insufficient sample size, we conducted learning curve analyses (Fig. 3). For both piperacillin and aztreonam, accuracy scores between the training and cross-validation sets of the random forest classifier analysis did not completely converge, but cross-validation scores did not substantially increase as training set sample size increased. These findings suggest that the poor performance of the random forest classifier approach for these two drugs is unlikely to be caused by an insufficient sample size. On the other hand, variables not included in this whole-genome feature set, such as epigenetic changes, could play an important role in dictating MICs for piperacillin and aztreonam.

We then extracted a ranking of k-mers according to their relative importance to the random forests model for gentamicin, fosfomycin, and cefoperazone-sulbactam (Table S5). Interestingly, for both gentamicin and cefoperazone-sulbactam, the top ranked k-mers were mapped to the class one integron region of *qnrVC1* bearing plasmid, which suggested this class one integron region could additionally carry beta‐lactamases in the ED sublineage. Additionally, k-mers corresponding to *pvdI* were also predicted to be associated with cefoperazone-sulbactam resistance. Similar association had been observed before [34]. For fosfomycin resistance, the k-mers with the highest importance aligned to genes encoding conjugative protein TraG, perhaps suggesting a general association of conjugative elements with fosfomycin resistance.

### Presence of virulence genes in the ED sublineage

To estimate if there were virulence gene loss or gain events that further defined the ED sublineage, we searched virulence genes in the ST316 lineage. A total of 230 virulence genes were identified from 192 ST316 isolates, and 173 of them were widely distributed (> 95 % in both ED and non-ED isolates) (Fig. S10). The number of virulence genes were significantly lower in ED isolates than in non-ED isolates (Fig. S11). We observed that a series of virulence genes involved in H1-type VI secretion system (*ppkA*), quorum sensing (*rhlI*), antimicrobial activity (*phzC1*) and iron uptake (*pchE/F/G/H*, *pvdD/J*) were less detected in ED isolates (*P*<1E-04, chi-square or Fisher’s exact test) (Table 3). Only two genes involved in antimicrobial activity (*phzG2* and *phzC2*) showed enrichment in the ED sublineage. Furthermore, the type III secretion system (T3SS) effector gene *exoU* was almost conserved in ST316 lineage (100 % in the ED sublineage, 93.02 % in non-ED isolates).

**Table 3.** Distribution of virulence genes between the ED sublineage and non-ED isolates Virulence genes leading to a *P* value<e-4 sorted by significance after performing a chi-square test or Fisher’s exact test between ED and non-ED isolates.

Gene	*P* value	ED sublineage (*n*=149)	non-ED isolates (*n*=43)	Category
*ppkA*	5.98E-18	41 (27.52 %)	42 (97.67 %)	Secretion system
*rhlI*	8.72E-16	55 (36.91 %)	43 (100.0 %)	Quorum sensing
*pchH*	9.51E-12	73 (48.99 %)	43 (100.0 %)	Iron uptake
*pchE*	8.22E-10	64 (42.95 %)	40 (93.02 %)	Iron uptake
*phzG2*	9.83E-09	146 (97.99 %)	28 (65.12 %)	Antimicrobial activity
*pchG*	1.31E-07	96 (64.43 %)	43 (100.0 %)	Iron uptake
*phzC2*	3.73E-07	65 (43.62 %)	2 (4.65 %)	Antimicrobial activity
*phzC1*	3.45E-06	75 (50.34 %)	38 (88.37 %)	Antimicrobial activity
*pvdJ*	2.04E-05	28 (18.79 %)	22 (51.16 %)	Iron uptake
*pchF*	5.95E-05	105 (70.47 %)	42 (97.67 %)	Iron uptake
*pvdD*	6.29E-05	4 (2.68 %)	10 (23.26 %)	Iron uptake

### ED sublineage defining mutations

ED sublineage specific mutations were detected in 25 genes (Table S4). The affected genes were mostly involved in metabolism or cellular processes and signalling. Notably, specific variants were detected in two genes encoding virulence factors in the ED sublineage: *pilU*, encoding twitching motility protein, and *lpxB*, encoding lipid-A-disaccharide synthase. PilU has been shown to be important for *in vitro* adherence [35]. LpxB is involved in lipid A biosynthesis. *P. aeruginosa* lipopolysaccharide (LPS) consisting of O-antigen, core oligosaccharide and lipid A has been shown to be crucial for mediating both bacterial virulence and host responses [36].

### Population structure of *P. aeruginosa* isolated from ear swabs

**Fig. 4.**
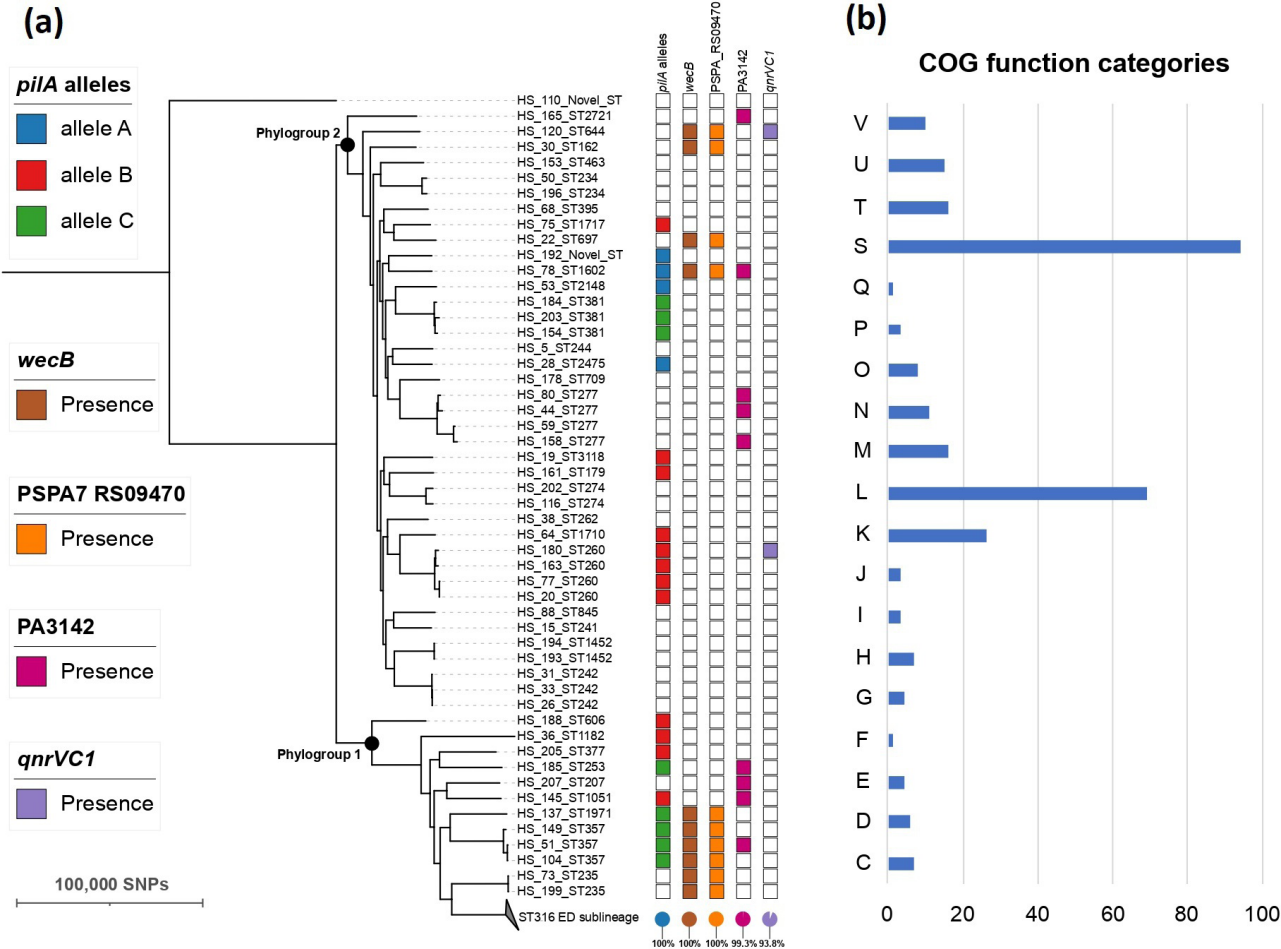
(**a**) Population structure of 199 *P *. *aeruginosa* ear swab isolates and (**b**) COG function assignment of 732 genes that were highly associated with ST316 isolates. The presence and absence of virulence or resistance genes highly associated with ST316 strains were denoted by filled and empty squares (right panel of the phylogenetic tree), respectively. COG functional categories: C, energy production and conversion; D, cell cycle control and mitosis; E, amino acid metabolism and transport; F, nucleotide metabolism and transport; G, carbohydrate metabolism and transport; H, coenzyme metabolism; I, lipid metabolism; J, translation; K, transcription; L, replication, recombination and repair; M, cell wall/membrane/envelop biogenesis; O, post-translational modification, protein turnover, chaperone functions; P, inorganic ion transport and metabolism; Q, secondary structure; S, function unknown; T, signal transduction; U, intracellular trafficking and secretion; V, defence mechanisms.

After examining the evolutionary trajectory of ST316 ED sublineage, we wanted to further explore genes highly associated with ST316 ED isolates when compared with other *P. aeruginosa* lineages isolated from ear swabs. Phylogenomic analysis of the 199 *P *. *aeruginosa* ear swab isolates revealed that most isolates (198/199) belonged to phylogroup 1 (79.90 %, 159/199) and phylogroup 2 (19.60 %, 39/199) (Fig. 4a). Only 12 non-ST316 strains belonged to phylogroup 1, therefore, most non-ST316 strains were *exoS* ^+^ isolates (76.92 %, 40/52) [37]. A genome-wide association study (GWAS) revealed that a total of 732 genes were significantly associated with ST316 strains (Fisher’s exact test, BH-adjusted p-value<1E-30) (Table S6). The associated genes were mostly enriched in replication, recombination and repair, which suggested recombination events could contribute to the success of ST316 strains (Fig. 4b). Interestingly, among the 732 genes, four are known virulence genes (type A *pilA* allele, *wecB*, PSPA7_RS09470, and PA3142) (Fig. 4a). The variation in the critical PilA structural pilin protein of T4aP has been noted [38]. The *pilA* gene was detected in 40.38 % (21/52) non-ST316 strains, and most of them (80.95 %, 17/21) belonged to the type B and C *pilA* alleles, typified by the protein under GenBank accession number WP_003122079.1 and WP_003141352.1 from LESB58 and UCBPP-PA14, respectively. All the ST316 strains carried a type A *pilA* allele homologous to NP_253215.1 from PAO1. The three encoded proteins shared only 42–79% amino acid identities. Furthermore, three virulence genes (*wecB*, PSPA7_RS09470, and PA3142) related to LPS were enriched in ST316 strains, which suggested additional differences in cell surface structure compared with non-ST316 ear swab isolates. The *qnrVC1* gene was also significantly enriched in ST316 strains when compared with non-ST316 strains, suggesting the expansion of ST316 strains could be associated with the acquisition of *qnrVC1*-bearing plasmid.

## Discussion

Although the *P. aeruginosa* ST316 clone was less frequently encountered in both hospital settings and community, our 2 year period surveillance of *P. aeruginosa* in an ENT hospital suggested ear infections caused by a FQr ST316 ED sublineage account for the most cases [8]. Overall, our data represented a comprehensive genomic analysis of the ST316 ED sublineage in Shanghai, China. We discovered that the ED sublineage possibly emerged in 2012 and was genetically close to an Indian isolate. In addition to Shanghai, we found two ED isolates were isolated from Guangzhou (a southeast province of China, 1 214 km away from Shanghai), providing evidence of cross-provincial dissemination.

In addition to the six recombination regions that were commonly found in the ST316 lineage, we also observed a recombination region was specifically detected in a subclade of ED isolates (ED*). The affected region encoded numerous of virulence factors including the complete siderophore pyoverdine synthesis locus, H3-type VI secretion system, and a GacA regulatory protein. Furthermore, using long-read sequencing we found that ED* isolates were further charactered as inactivation of a *fiuA* gene by an *IS*Pa62 element. These findings provided evidence that recombination events might play an important role in the pathogenicity of the ED sublineage [22].

We observed a *qnrVC1*-bearing MDR plasmid was most stably maintained in the ED sublineage. It is possible that *qnrVC1*-producing strains acquired this epidemic plasmid while circulating locally in Shanghai. Consistent with this idea, the *qnrVC1*-bearing plasmid reported here shared a similar backbone with an IncP-2 plasmid sublineage (pHS17-127) associated with dissemination of *bla*_IMP-45_ among CR *P. aeruginosa* that was described by our previous work (Table S2) [39]. It has been previously proposed that acquisition of low-level quinolone resistant genes such as *qnrVC1* may create an environment facilitating the selection of more highly resistant determinants such as mutations in DNA gyrase [40]. Therefore, transmission of this plasmid should be closely monitored, especially in the wake of increased MDR *P. aeruginosa* incidence.

Mutational analysis suggested mutations in genes involved in *mex*-type multidrug resistance efflux pump played important roles in inducing the MDR phenotype in the ED sublineage. Furthermore, we also identified novel mutations in *fusA1* and *parE* which could be involved in aminoglycoside and FQ resistance, respectively, in the ED sublineage. Signs of strong selective pressure were observed in genes associated with drug resistance (*mexR*, *mexB*, *mexC*, *parS*, and *parE*). Effective treatment mainly relies on fluoroquinolones, aminoglycosides, and β-lactams. In this context, the repeated, independent emergence of variants associated with MDR is particularly worrisome and raises the concern of the emergence of an MDR ST316 ED sublineage causing ear infections. Furthermore, we showed that a k-mer based machine learning model was predictive of gentamicin, fosfomycin, and cefoperazone-sulbactam resistance, however, it was less predictive of piperacillin and aztreonam resistance. Interestingly, a recent research used a support vector classifier machine learning model trained with both whole-genome sequence data and gene expression profiles of *P. aeruginosa* isolates, that identified similar biomarkers which are responsible for aminoglycoside and cephalosporin resistance (i.e. tobramycin and ceftazidime) [34]. However, the previous study did not report the genomic locations of these biomarkers. In our research, the top ranked k-mers associated with gentamicin and cefoperazone-sulbactam resistance were located on the MDR region of the *qnrVC1* bearing plasmid, which suggested this region could be important for developing drug resistance for the ED sublineage.

We observed that gene loss events frequently occurred among virulence genes involved in H1-type VI secretion system (*ppkA*), quorum sensing (*rhlI*), antimicrobial activity (*phzC1*) and iron uptake (*pchE/F/G/H*, *pvdD/J*) compared to non-ED ST316 isolates. It was likely that the ED sublineage tended to be less virulent, as the number of virulence genes in the ED sublineage was significantly lower than that in non-ED isolates. Previous study has revealed that *P. aeruginosa ppkA* null mutant exhibited reduced virulence in neutropenic mice [41]. Recent study showed that wild-type *P. aeruginosa* was more attractive prey for macrophages or stimulated their own ingestion compared to the strain with defective mutations in the *lasI* and *rhlI* genes [42]. It was possible that loss of *rhlI* could promote extracellular multiplication of ED isolates. In addition, high concentrations of intracellular iron may facilitate rapid FQ-mediated killing, thus loss of genes involved in iron uptake could increase fitness in the ED sublineage subjected to FQs [43]. Furthermore, specific mutations in genes coding surface structures such as, *pilU* and *lpxB*, probably contributed to the adaptation of the ED sublineage in the ear canal.

As resistance to FQs was also observed in other *P. aeruginosa* lineages isolated from ear swabs during our survey (Table 1), one could imagine that the success of the ED sublineage ear infection patients did not fully rely on resistance. Compared with non-ST316 ear swab isolates, the *exoU* gene was enriched in the ED sublineage. It was evidenced that *exoU* ^+^ isolates are more likely to be FQ-resistant than *exoS* ^+^, as well as more likely to acquire resistance-conferring mutations in *gyrA/B* and *parC/E* [44]. Additionally, the *pilA* gene which was important for host cell colonization [45] was conserved in ST316 strains, while only detected in 40.38 % non-ST316 isolates. Furthermore, the *pilA* gene in ST316 isolates belonged to an A-type allele, however most *pilA* in non-ST316 isolates belonged to B- or C-type alleles. In additation, specific LPS-related virulence genes could also contribute to the pathogenicity of the ED sublineage, as these surface structures played important roles in host-pathogen interactions.

This study has some limitations. First, all *P. aeruginosa* ear swab isolates analysed in this study were collected from Shanghai; therefore, the results may lack external validity. Second, direct evidence indicating that the ST316 Shanghai sublineage is a high-risk clone causing aural infection is lacking. To address this problem, experimental animal models should be investigated. Third, the lack of publicly available ST316 genomes and detailed epidemiological information makes it difficult to accurately trace the emergence and evolution of the ST316 ED sublineage.

In summary, we characterized detailed genomic features of an emerging ST316 ED sublineage that spread to cause ear infection epidemics in Shanghai. Our results suggested that this clone emerged recently and tended to be MDR. Classical mutations in QRDRs, acquisition of MDR plasmid, and mutations in MDR determinants were identified in this clone. Specific mutations in virulence determinants and loss of certain virulence genes could have contributed to its adaption in ear canal. Furthermore, specific virulence genes associated with cell surface structure could make ST316 strains an ear infection high-risk clone. Importantly, our analysis suggested the *qnrVC1* bearing plasmid was important for the success of the ED sublineage.

## Supplementary Data

Supplementary material 1
