# Supplementary material for: Dissecting the genotypic features of a fluoroquinolone-resistant Pseudomonas aeruginosa ST316 sublineage causing ear infections in Shanghai, China
Source: Microb Genom. 2023 Apr 20;9(4):mgen000989. doi: 10.1099/mgen.0.000989 (PMC10210959; doi:10.1099/mgen.0.000989)
Supplement: Supplementary material 1 [file mgen-9-989-s001.pdf]

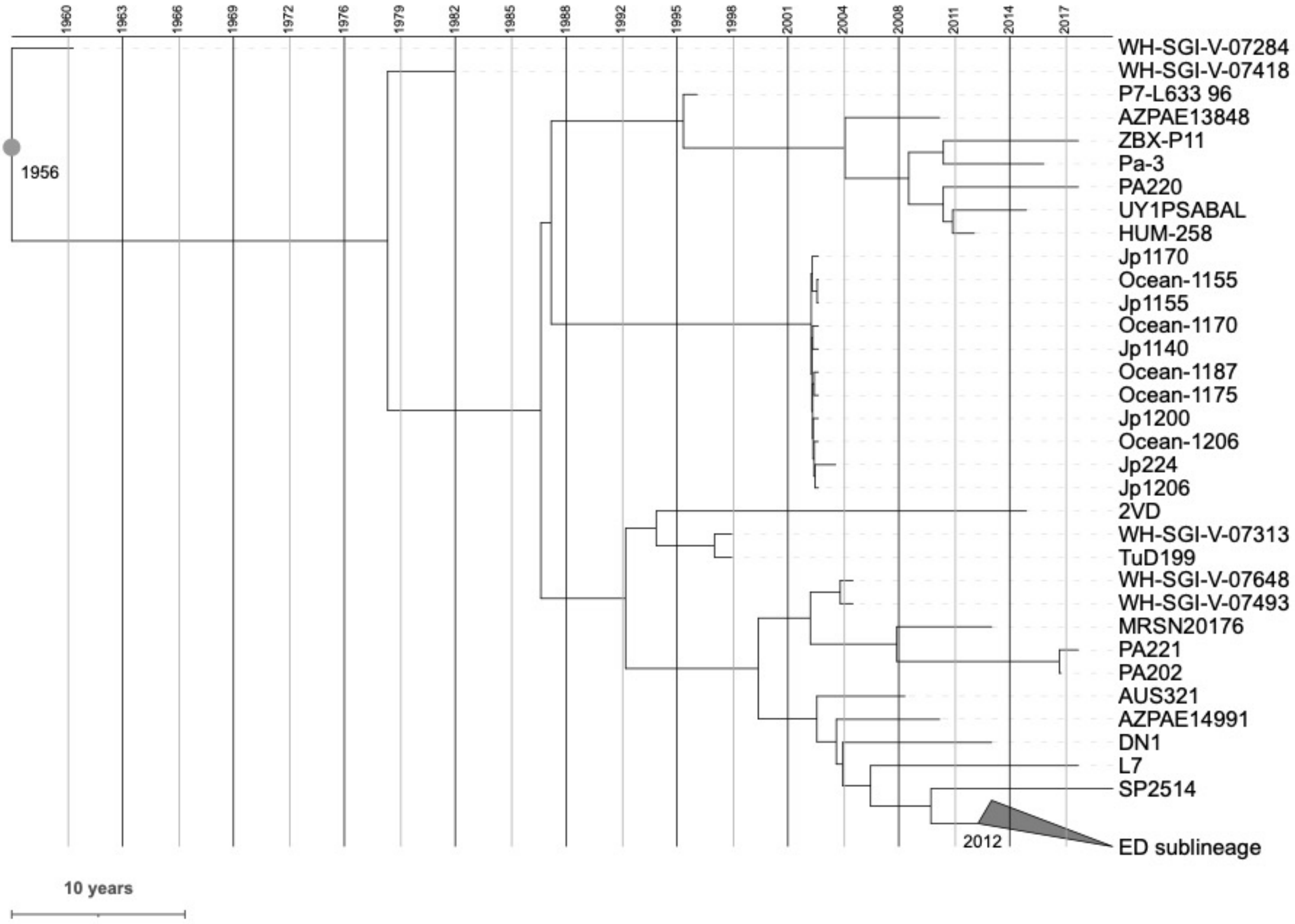

Fig. S1: Bayesian tree inference of *P. aeruginosa* ST316 lineage. Selected divergence time are shown at the nodes. The estimated mutation rate was 4.17 x 10^{-6} (95% HPD 3.89 x 10^{-6} to 4.44 x 10^{-6}) per site per year, which was largely in line with previous evolutionary rates reported for *P. aeruginosa(1, 2)*.

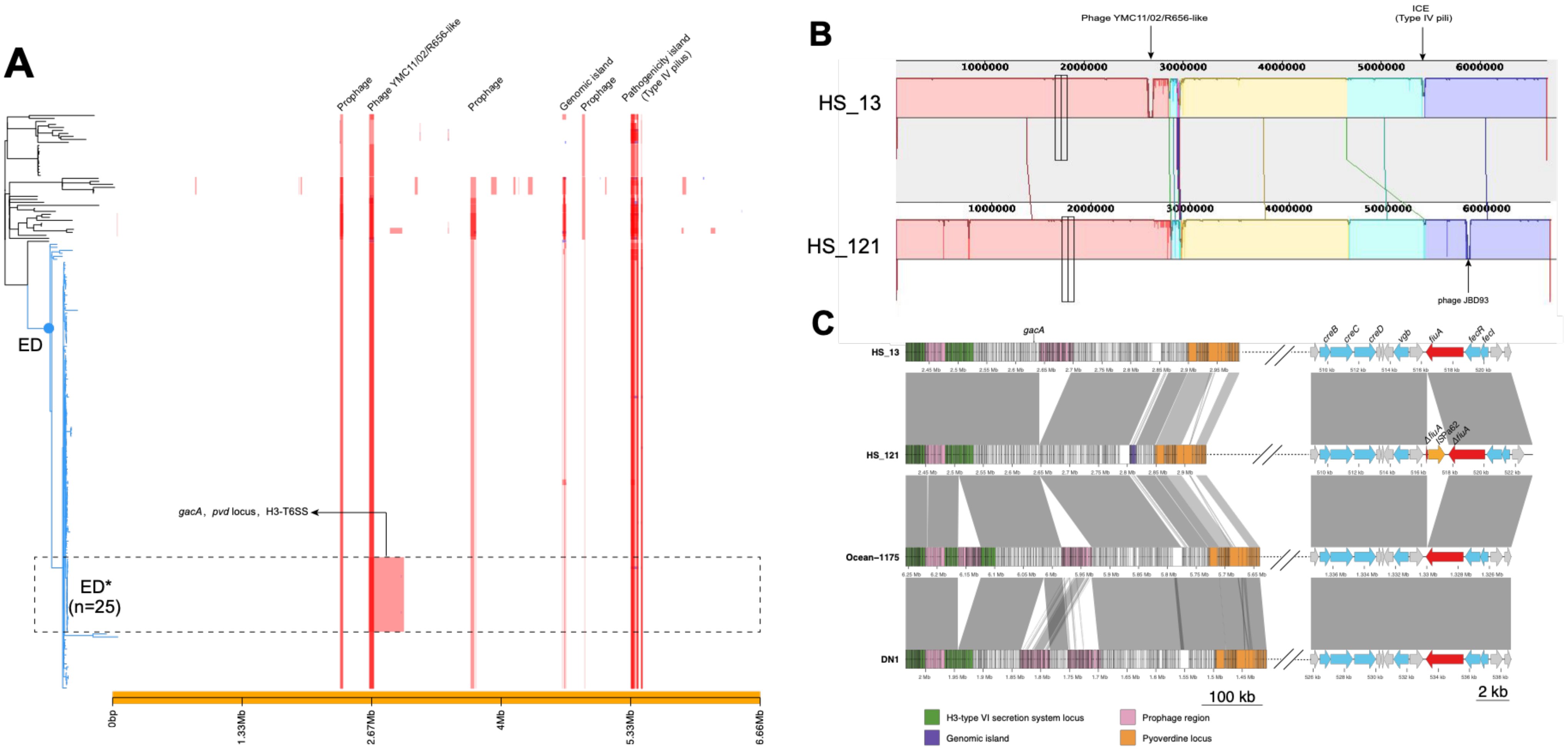

Fig. S2: Distribution of recombination events across the ST316 lineage and whole genome alignment between HS_13 (ED) and HS_121 (ED*). (A) The phylogeny (left) s displayed alongside the linearized chromosome (right). Red blocks (right) indicate inferred recombination events each affecting selected taxa and genomic location. **(B)** Comparison of global genomic synteny of HS_13 to HS_121. Genomic synteny plot was generated using Mauve v2.3.1(3). **(C)** Comparison of the specific recombination region and inactivation of *fiuA* detected in ED* (represented by HS_121) to reference HS_13 and two non-ED sublineage ST316 strains, Ocean-1175 and DN1. The recombination region is frequently affected by prophages. The *fiuA* gene in HS_121 is affected by an *IS*Pa62 element when compared to other ST316 isolates.

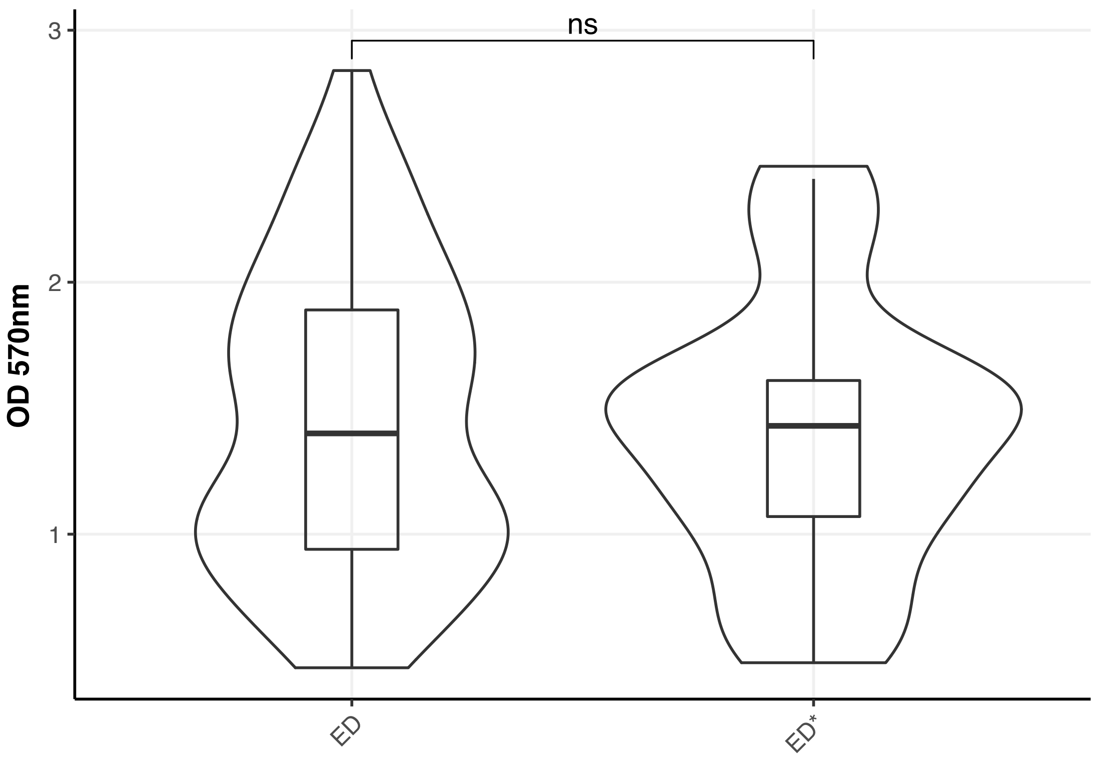

Fig. S3: Biofilm formation ability (OD570; y-axis) of 122 ED isolates other than ED* and 25 ED* isolates. Biofilm data was taken from our previous work(4).

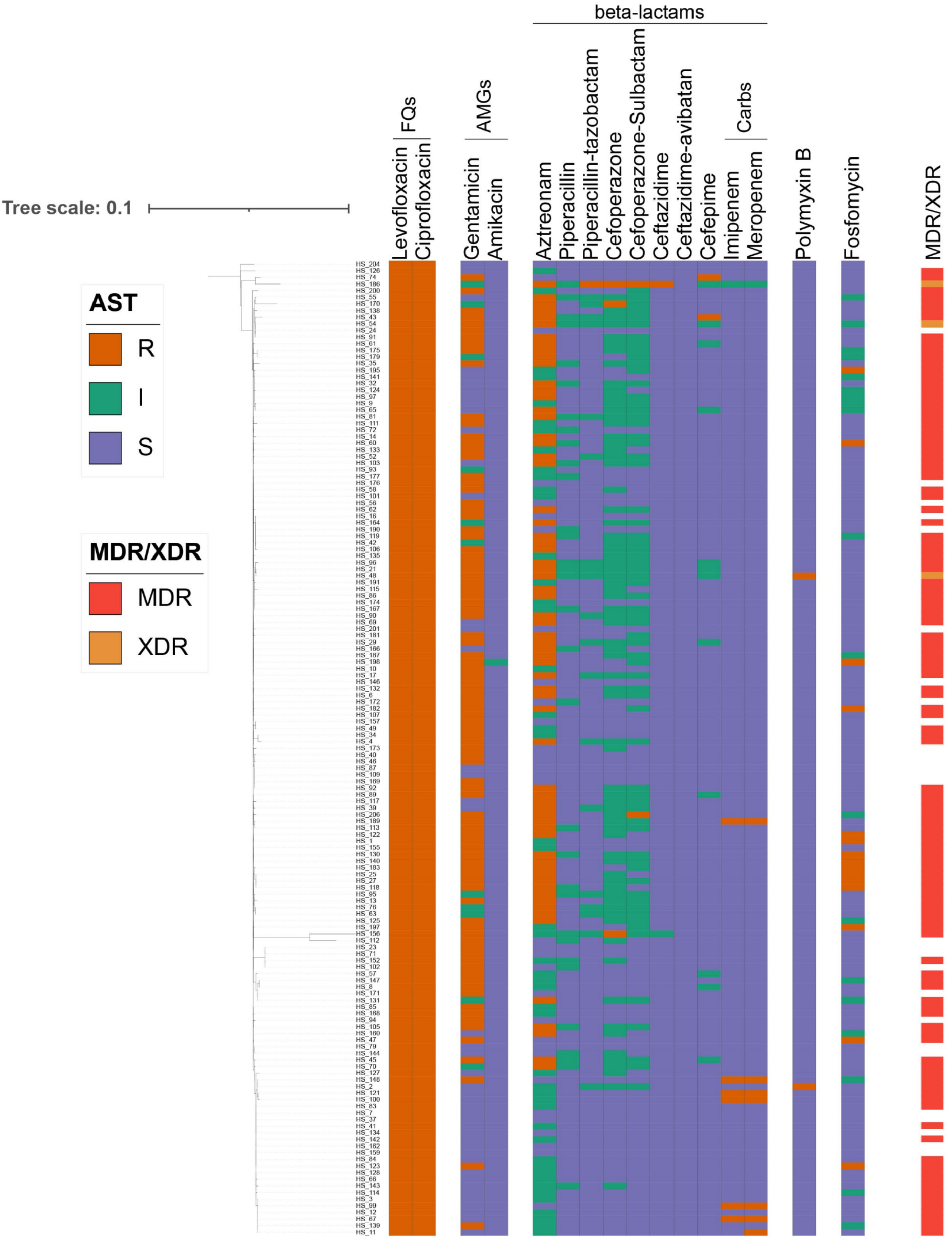

Fig. S4: Antimicrobial susceptibility of 147 ED isolates from our collection. R, resistant; I, intermediate; S, susceptible; FQs, fluoroquinolones; AMGs, aminoglycosides; Carbs, carbapenems; MDR, multiple-drug resistant; XDR, extensively drug-resistant. Antimicrobial susceptibility data was taken from our previous publication(4).

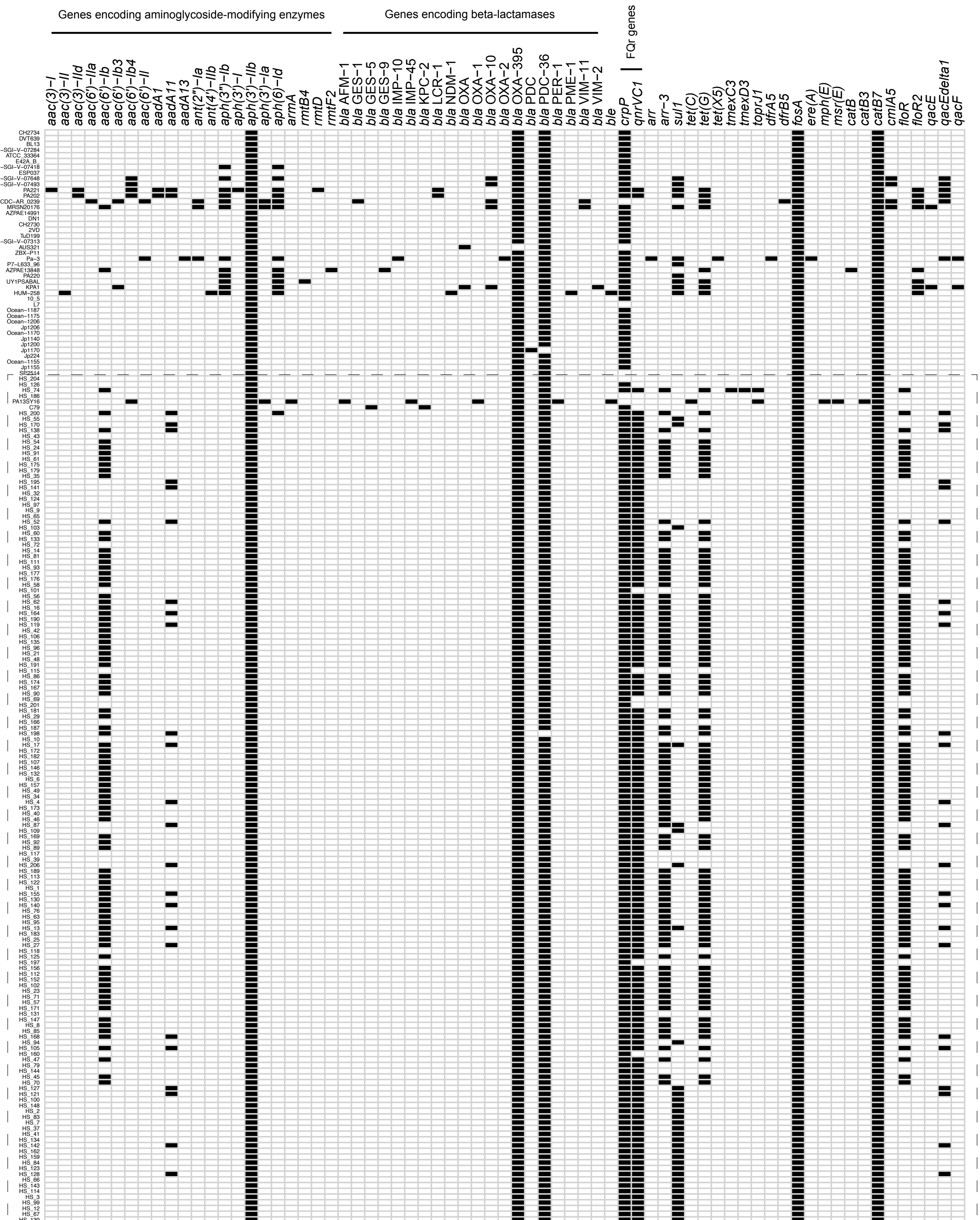

Fig. S5: Distributions of acquired AMR genes in the ST316 lineage. Squares, which are colored in black represent the presence of the AMR gene. ED sublineage was framed by a dashed line box.

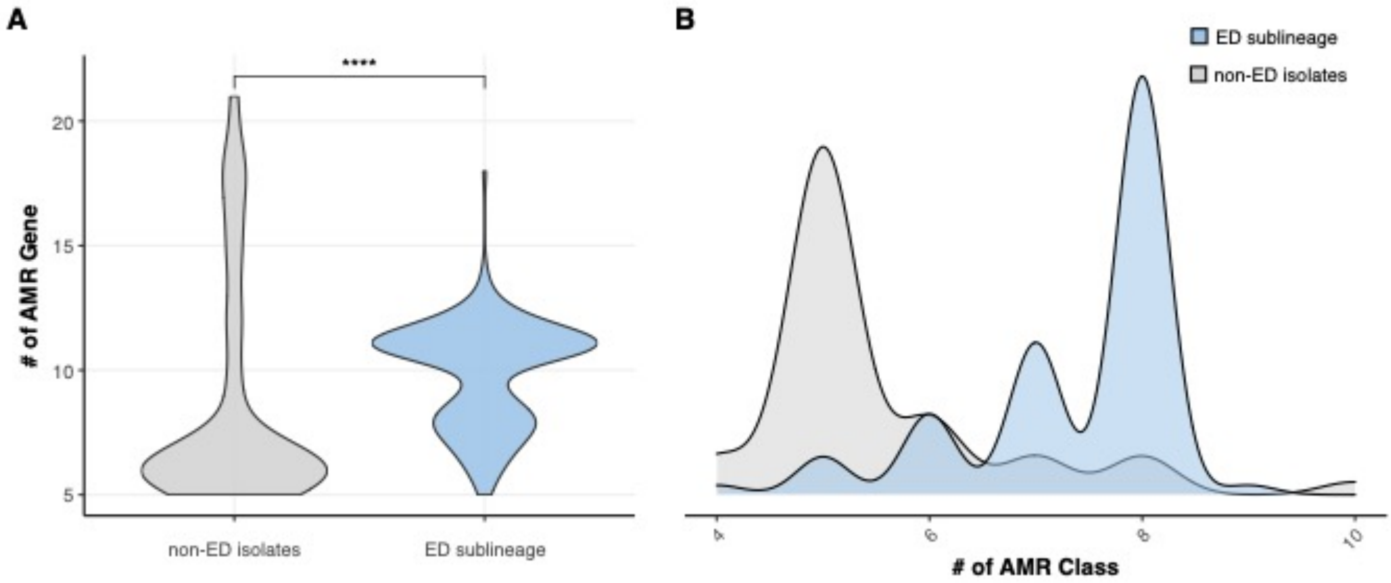

Fig. S6: Comparison of acquired AMR genes between non-ED and ED isolates. (A) Number of acquired AMR genes per isolate in non-ED and ED. ****, P < 0.00001. (B) Density plot showing the distributions of number of drug classes for which acquired resistance determinants were detected in each genome.

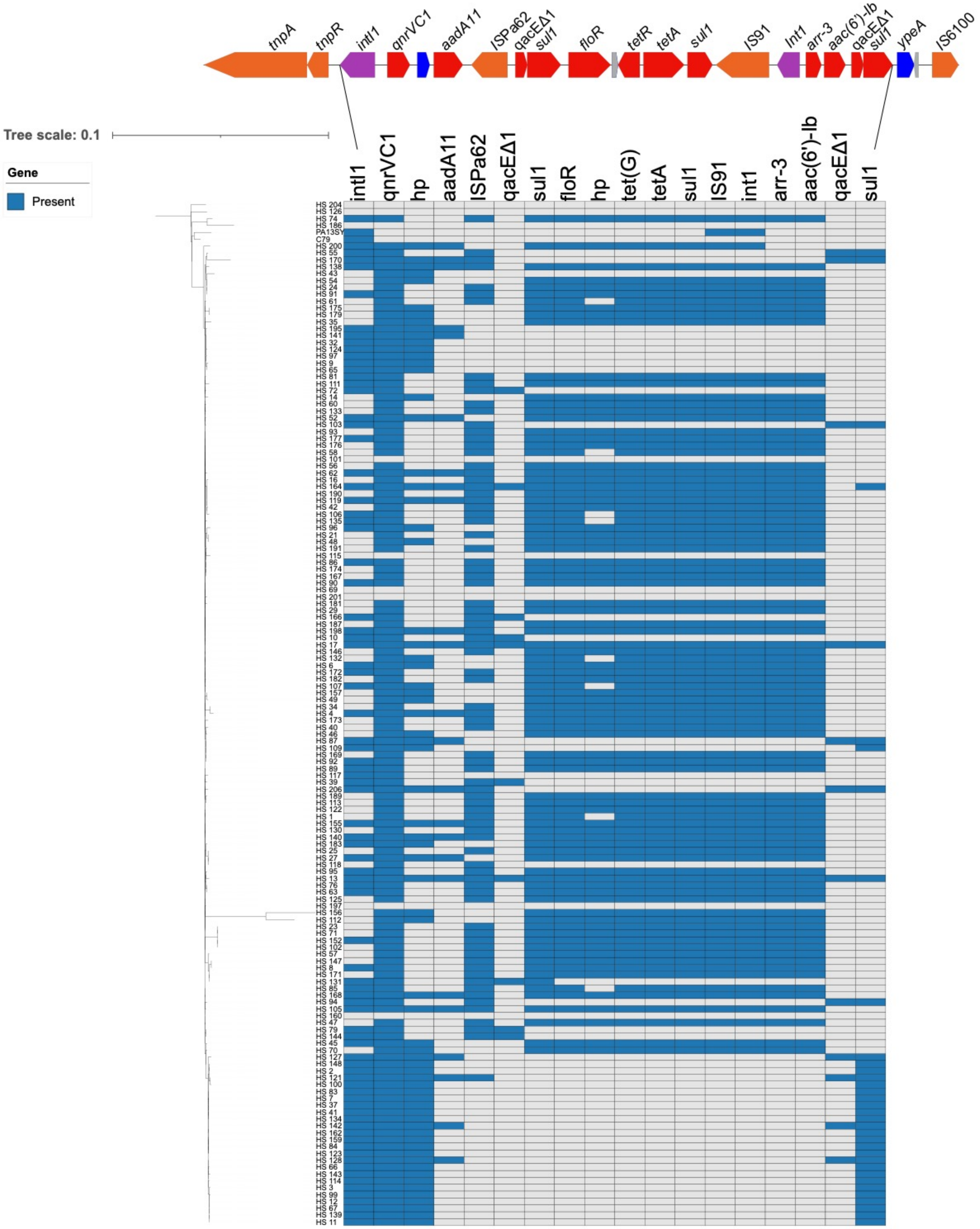

Fig. S7: Comparison of class I integron on the *qnrVC1*-bearing plasmid across ED sublineage. The complete integron (top) carrying *qnrVC1* from HS_13 is shown, genes are represented by arrow boxes. The heatmap showed the presence or absence of genes on this integron, Squares, which are colored in blue represent the presence of this gene. The presence/absence pattern was detected using Panaroo v1.2.10 with a sensitive mode(5).

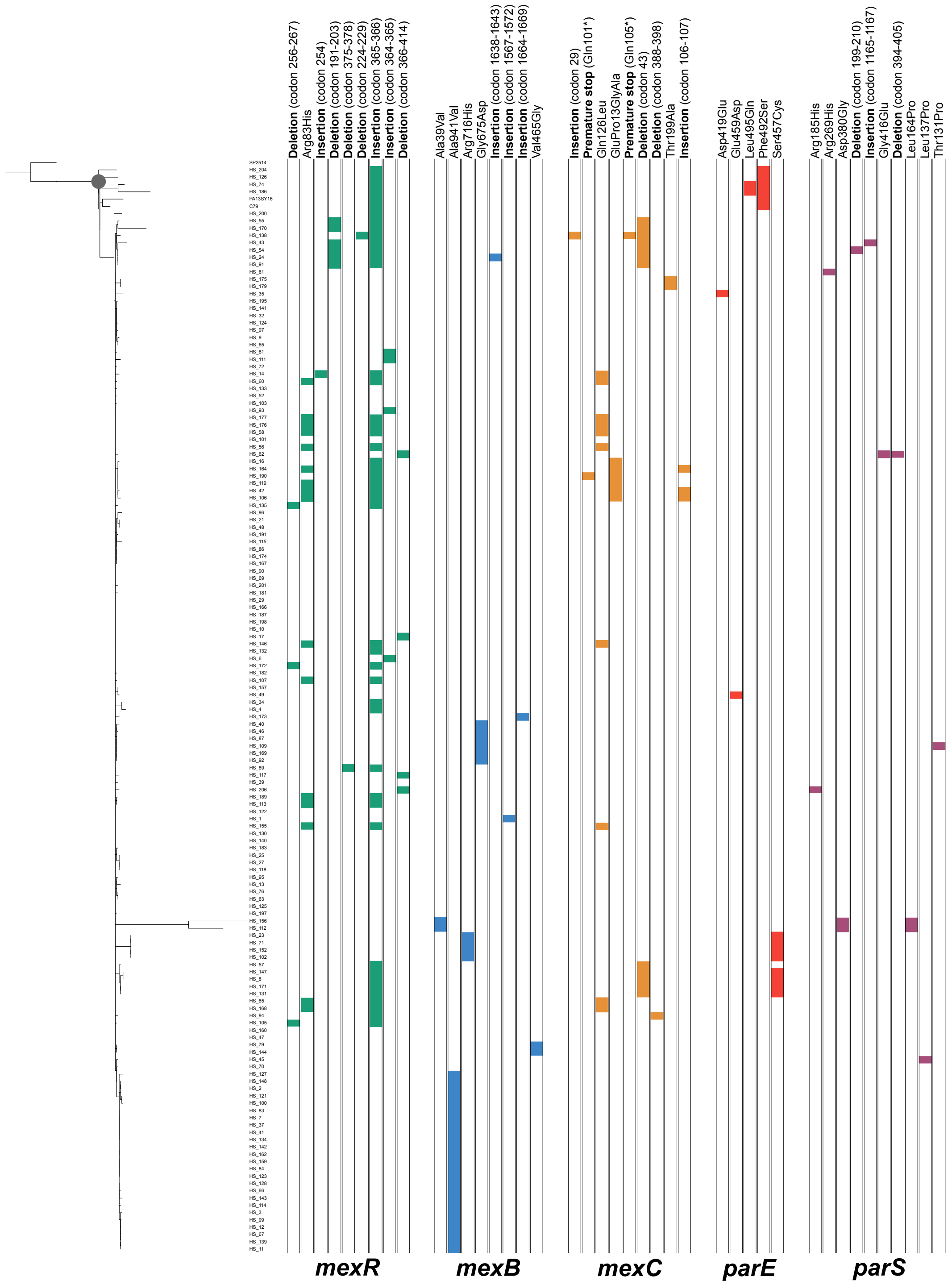

Fig. S8: Convergent evolution on resistance genes. A maximum likelihood phylogenetic tree (strain SP2514 as an outgroup) for ED sublineage (left) with the mutational events of resistance genes (right) that are repeatedly and independently mutated among the outbreak isolates. Each track represents a unique mutation within a gene. HS_13 was the reference genome.

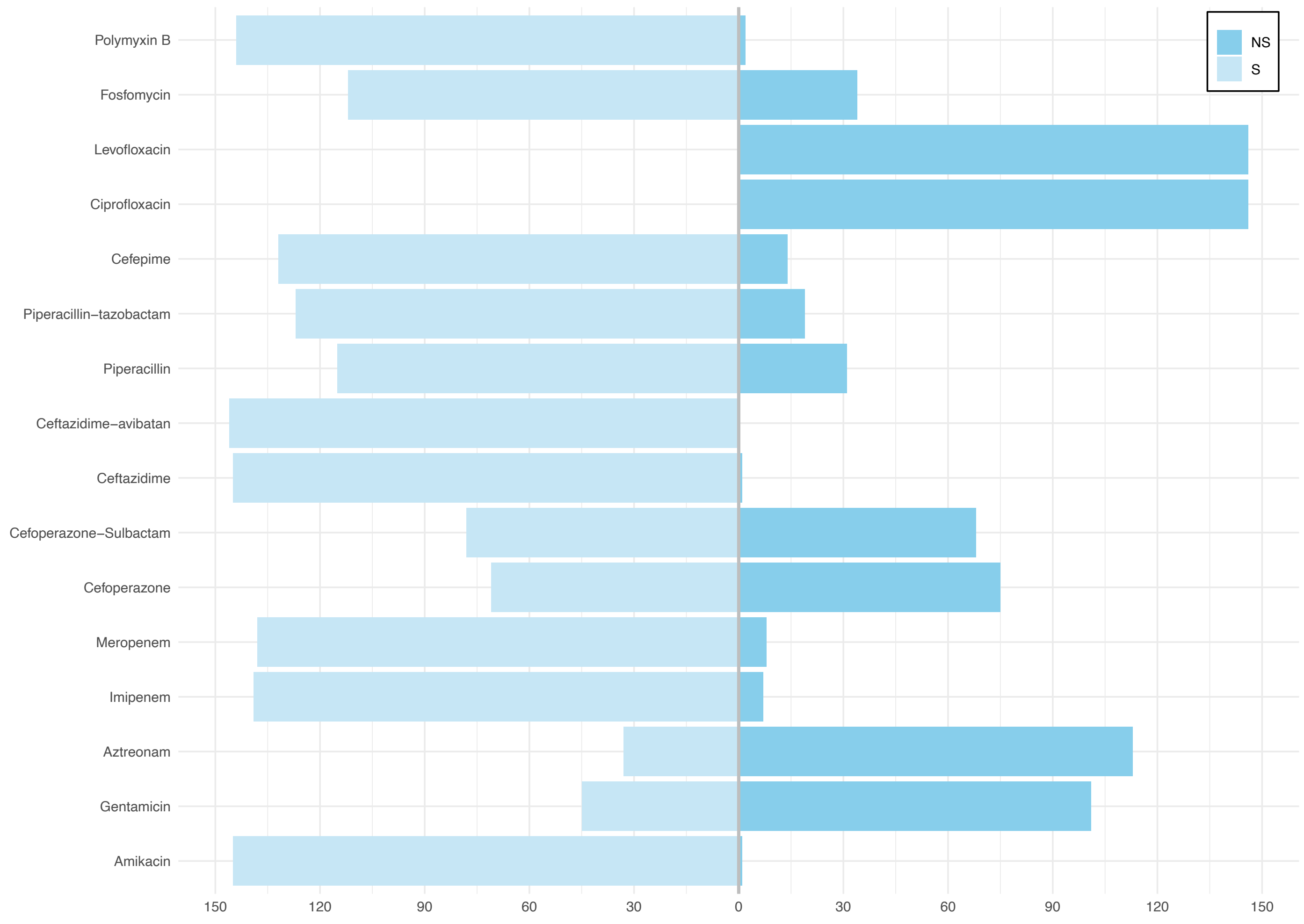

Fig. S9: distribution of susceptibility (S) and non-susceptibility (R and I) strains (totally 147 isolates) across each antibiotic.

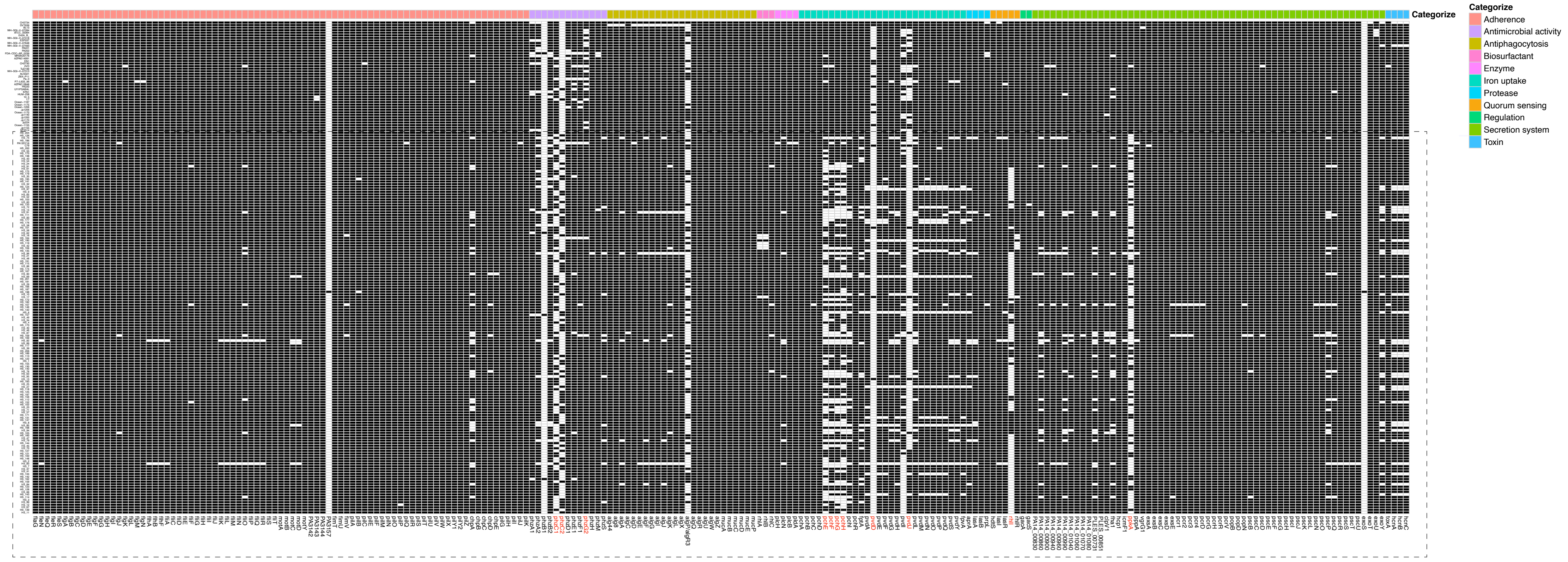

Fig. S10: Distribution of virulence genes across ST316 lineage. Virulence genes that are significantly differed (P < 0.0001, chi-square test or Fisher's exact test) between ED and non-ED isolates are colored in red (top). Squares, which are colored in black represent the presence of this gene. ED sublineage was framed by a dashed line box.

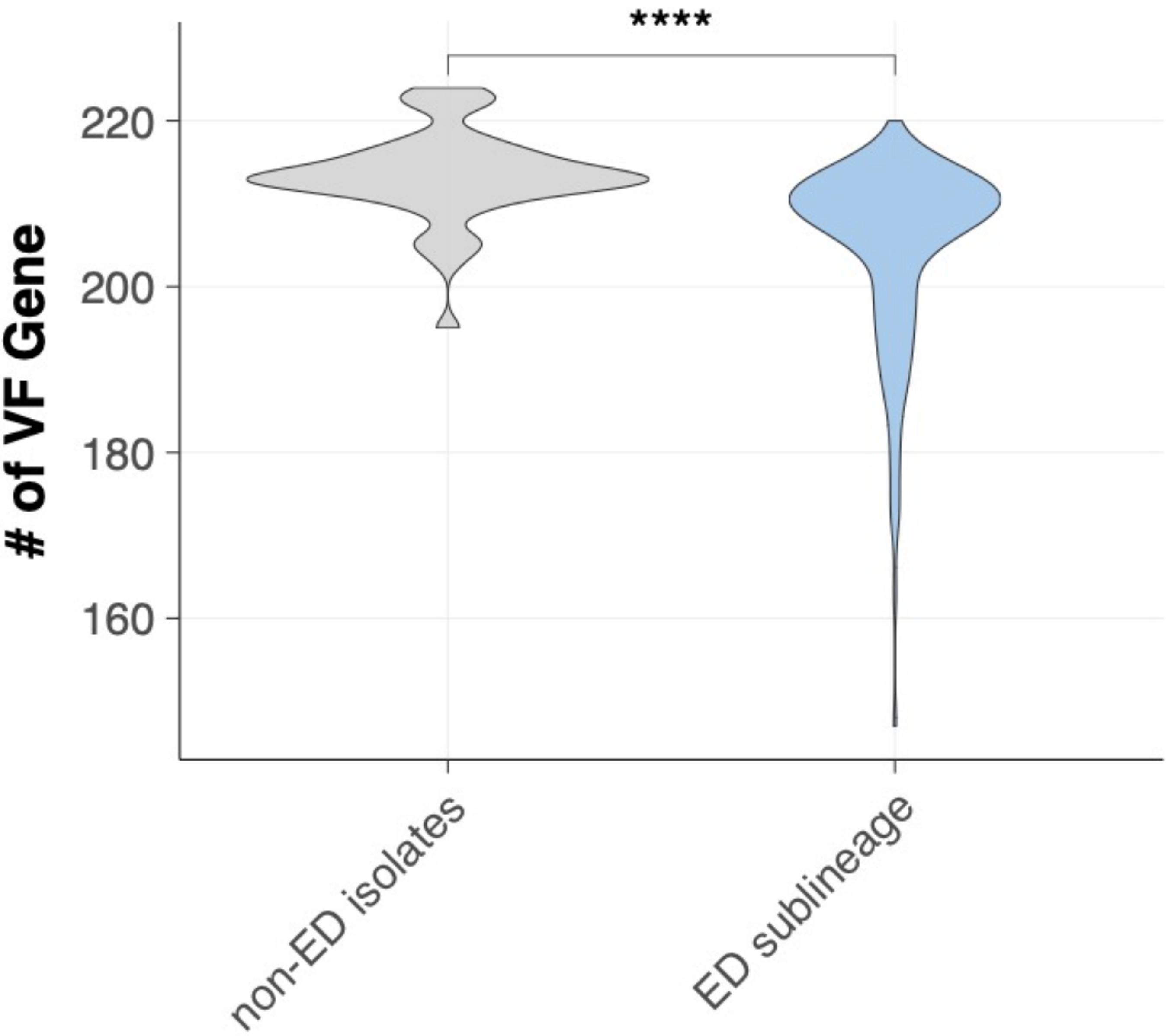

Fig. S11: Comparison of acquired virulence genes between non-ED and ED isolates. Number of virulence genes per isolate in non-ED and ED. ****, P < 0.00001.

Strain	BioSample Accession	Collection Date	Geographic Location	Iatitude and Longitude	Isolation Source
10_5	SAMEA3296136				Environment
2VD	SAMN08712272	2015	Trento (Italy)	46.01 N 10.52 E	Environment
ATCC_33364	SAMN04028053				Unknown
AUS321	SAMN07423938	2008	Mundubbera (Australia)		Environment
AZPAE13848	SAMN03105417	2010	India		Unknown
AZPAE14991	SAMN03105688	2010	Santander (Spain)		Abdominal cavity
BL13	SAMN02360726		United States		Eye
C79	SAMN11836198	2017	China		Unknown
CH2730	SAMN11110369		Berlin (Germany)		Unknown
CH2734	SAMN11110370		Berlin (Germany)		Unknown
DN1	SAMN05717682	2013	China		Environment
DVT639	SAMN14271779		Pennsylvania, Pittsburgh (United States)	40.44 N 79.99 W	Unknown
E42A_B_	SAMN05226612		Bucarest (Romania)		Environment
ESP037	SAMN11110495		Palma de Mallorca (Spain)		Unknown
FDA-CDC-AR_0239	SAMN04901629				Respiratory tract
HUM-258	SAMN09477992	2012	Estonia	58.36 N 26.68 E	Wound
Jp1140	SAMN07424069	2003	Japan		Environment
Jp1155	SAMN10478477	2003	Japan		Environment
Jp1170	SAMN10478478	2003	Japan		Environment
Jp1200	SAMN10478479	2003	Japan		Environment
Jp1206	SAMN10478480	2003	Japan		Environment
Jp224	SAMN07424067	2004	Japan		Environment

KPA1	SAMN15083412		Kenya		Unknown
L7	SAMN12712224	2018	Gungdong (China)		Respiratory tract
MRSN20176	SAMN09788414	2013	Afghanistan		Skin
Ocean-1155	SAMN07347483	2003			Environment
Ocean-1170	SAMN07347484	2003			Environment
Ocean-1175	SAMN07347485	2003			Environment
Ocean-1187	SAMN07347489	2003			Environment
Ocean-1206	SAMN07347490	2003			Environment
P7-L633_96	SAMN02720833	1996	India	17.42 N 78.42 E	Eye
PA13SY16	SAMN04966046	2013	Gungdong (China)		Urine
PA202	SAMN13340388	2017	India		Eye
PA220	SAMN13340394	2018	India		Eye
PA221	SAMN13340395	2018	India		Eye
Pa-3	SAMN14463699	2016	Pakistan	33.68 N 73.04 E	Respiratory tract
SP2514	SAMN18310626	2020	Vellore (India)	12.92 N 79.13 E	Respiratory tract
TuD199	SAMN07424093	1998	Tunis (Tunisia)		Respiratory tract
UY1PSABAL	SAMN12261616	2015	Yaounde (Cameroon)		Respiratory tract
WH-SGI-V-07284	SAMN04128727	1960	Romania		Environment
WH-SGI-V-07313	SAMN04128745	1998	Tunisia		Respiratory tract
WH-SGI-V-07418	SAMN04128592	1982			Unknown
WH-SGI-V-07493	SAMN04128612	2005	United States		Clinical Environment
WH-SGI-V-07648	SAMN04128658	2005	United States		Clinical Environment
ZBX-P11	SAMN16132399	2018	Beirut (Lebanon)	33.90 N 35.49 E	Clinical Environment

Tab. S1: meta information of the 45 publicly available ST316 strains used in this study.

Query	Subject	Subject accession	Subject geographic location	Subject organism	Subject ST	Identity (%)	Coverage (%)
pHS13-391	pR31014-IMP	MF344571.1	Beijing, China	*Pseudomonas aeruginosa* R31014	-	99.85	80.00
pHS13-391	pKB-PA_F19-4	CP086014.1	Yunnan, China	*Pseudomonas aeruginosa* KB-PA_F19	ST244	99.94	91.00
pHS13-391	p243931-IMP	MN208062.1	Beijing, China	*Pseudomonas aeruginosa* 243931	ST235	99.83	84.00
pHS13-391	p60503-DIM	MN208063.1	Beijing, China	*Pseudomonas aeruginosa* 60503	ST773	97.47	88.00
pHS13-391	pNDTH9845	CP073081.1	Zhejiang, China	*Pseudomonas aeruginosa* NDTH9845	ST463	99.93	92.00
pHS13-391	pHS17-127*	CP061377.1	Shanghai, China	*Pseudomonas aeruginosa* HS17-127	ST369	98.47	94.00
pHS13-391	p727-IMP	MF344568.1	Beijing, China	*Pseudomonas aeruginosa* 727	-	99.91	86.00
pHS13-391	pSY153-MDR	KY883660.1	Beijing, China	*Pseudomonas putida* SY153	-	99.99	93.00
pHS13-391	p519119-DIM	MN208061.1	Beijing, China	*Pseudomonas aeruginosa* 1705-19119	ST360	97.47	88.00
pHS13-391	pBJP69-DIM	MN208064.1	Beijing, China	*Pseudomonas sp.* BJP69	-	97.47	88.00
pHS13-391	p12969-DIM	KU130294.1	Chongqing, China	*Pseudomonas putida* 12969	-	97.87	88.00
pHS13-391	pWTJH17	CP073083.1	Zhejiang, China	*Pseudomonas aeruginosa* WTJH17	ST260	98.60	92.00
pHS13-391	pBM413	CP016215.1	Guangdong, China	*Pseudomonas aeruginosa* PA121617	ST389	99.94	93.00
pHS13-391	pPAG5	CP045003.1	Shanxi, China	*Pseudomonas aeruginosa* PAG5	ST639	99.84	94.00
pHS13-391	pA681-IMP	MF344570.1	Beijing, China	*Pseudomonas aeruginosa* A681	ST274	99.72	83.00

Tab. S2: BLASTN hits of the *qnrVC1*-bearing plasmid in ear swab isolates. Complete plasmid sequence, pHS13-391, from HS13 is used as a query. *An IncP-2 plasmid carrying IMP-45 that reported by our previous work.

Strain	GEN	IMP	TZP	PIP	CZA	CAZ	FOS	MEM	CIP	LEV	AMK	ATM	SCF	CEF	CFP	POL B
HS_1	64	1	4	8	2	1	>128	<0.06	>128	>128	4	16	16	2	16	1
HS_2	0.13	1	32	8	2	2	64	0.5	>128	>128	0.25	16	32	2	32	8
HS_3	2	1	16	16	2	2	64	0.25	>128	>128	2	16	16	4	16	0.5
HS_4	32	1	32	16	8	8	64	0.25	>128	>128	4	32	32	8	32	0.5
HS_5	1	1	4	8	1	2	64	1	0.13	0.5	1	4	4	2	4	1
HS_6	64	1	16	16	8	8	64	0.25	>128	>128	4	32	32	8	32	0.5
HS_7	2	1	16	8	2	2	64	0.25	>128	>128	2	8	16	4	16	2
HS_8	128	1	16	16	4	4	32	0.5	>128	>128	8	16	16	16	16	0.5
HS_9	2	1	16	16	4	8	128	0.25	>128	>128	2	16	32	8	32	0.5
HS_10	64	1	16	16	4	8	64	0.25	>128	>128	4	16	16	4	16	1
HS_11	1	2	16	16	2	2	64	8	>128	>128	2	16	16	4	16	0.5
HS_12	4	1	16	16	2	2	64	0.13	>128	>128	4	16	16	8	16	1
HS_13	16	1	16	16	8	8	64	0.25	>128	>128	2	32	32	8	32	0.5
HS_14	128	1	16	16	4	8	64	0.25	>128	>128	4	32	32	8	32	0.5
HS_15	2	1	4	4	2	2	16	0.5	0.25	0.5	4	4	4	4	4	0.5
HS_16	64	1	1	2	0.5	2	64	<0.06	>128	>128	4	2	4	4	4	0.5
HS_17	64	1	32	16	8	8	64	0.25	>128	>128	4	32	32	8	32	1
HS_19	2	0.5	8	4	2	2	128	0.25	0.25	1	4	4	4	2	4	0.5
HS_20	2	1	4	4	1	2	8	0.25	0.13	1	4	4	4	2	4	0.5
HS_21	64	1	32	32	8	8	64	0.5	>128	>128	8	32	32	16	32	1
HS_22	>128	0.5	1	2	0.5	1	32	<0.06	>128	>128	>128	2	4	4	1	0.25
HS_23	64	1	8	16	1	2	32	0.13	>128	>128	8	4	16	8	8	1
HS_24	16	0.5	4	4	0.25	0.5	32	<0.06	128	>128	2	0.5	4	1	4	0.5
HS_25	32	1	16	16	4	8	>128	0.25	>128	>128	4	32	16	8	32	0.5
HS_26	2	1	16	4	1	2	32	0.25	1	8	2	2	4	2	4	0.5
HS_27	32	1	16	16	8	8	>128	0.25	>128	>128	4	32	32	8	32	1
HS_28	1	1	16	16	4	4	16	2	0.5	128	1	32	32	8	32	1
HS_29	64	1	32	16	8	8	64	1	>128	>128	8	32	32	16	32	2
HS_30	64	1	16	16	4	4	64	0.5	>128	>128	4	32	32	8	32	1
HS_31	2	1	4	4	1	2	16	0.25	0.25	1	4	2	4	4	4	1
HS_32	4	1	16	32	4	8	64	0.25	>128	>128	4	32	32	8	32	1

HS_33	1	1	2	4	1	2	32	0.25	2	8	2	4	4	2	4	1
HS_34	32	1	16	16	4	4	64	0.13	>128	>128	4	16	16	8	16	2
HS_35	64	1	16	32	8	8	32	0.25	>128	>128	4	32	32	8	32	0.5
HS_36	2	1	2	4	1	1	32	<0.06	0.13	0.5	2	4	4	2	4	0.5
HS_37	1	1	16	16	2	2	64	0.25	>128	>128	2	8	16	4	16	0.5
HS_38	2	0.5	16	16	8	8	64	0.5	1	4	4	32	32	16	32	0.5
HS_39	2	1	32	16	8	8	64	0.25	>128	>128	4	32	32	8	32	1
HS_40	32	1	2	2	0.5	0.5	64	<0.06	>128	>128	4	2	2	2	4	0.5
HS_41	1	1	16	16	2	2	64	0.25	>128	>128	2	16	16	4	16	0.5
HS_42	8	1	16	16	8	8	64	0.13	>128	>128	0.5	32	32	8	32	2
HS_43	>128	1	32	32	8	8	8	1	>128	>128	8	32	32	32	32	1
HS_44	4	2	4	4	2	2	64	1	1	2	4	4	4	4	4	1
HS_45	64	2	16	32	8	8	64	1	>128	>128	8	32	32	16	32	1
HS_46	32	1	2	2	0.5	1	64	0.06	>128	>128	4	2	2	2	4	0.5
HS_47	32	1	8	16	1	2	>128	0.13	>128	>128	4	8	16	2	16	1
HS_48	64	1	32	32	8	8	64	0.5	>128	>128	8	32	32	16	32	4
HS_49	>128	2	16	4	2	4	64	0.13	>128	>128	16	16	16	8	16	2
HS_50	4	2	16	4	1	2	64	<0.06	0.25	1	4	4	4	2	4	1
HS_51	4	1	16	8	4	4	32	0.25	0.5	2	4	16	16	8	16	1
HS_52	32	1	32	16	8	8	64	0.25	>128	>128	2	32	32	8	32	0.5
HS_53	2	0.5	1	1	0.5	0.5	32	<0.06	0.25	1	4	2	2	2	1	0.5
HS_54	128	1	32	32	8	8	128	0.5	>128	>128	8	32	32	16	32	0.5
HS_55	2	0.5	32	32	8	8	128	0.13	>128	>128	4	32	32	8	32	1
HS_56	128	1	2	2	1	1	64	<0.06	>128	>128	8	2	4	4	4	1
HS_57	128	1	8	16	4	4	64	0.25	>128	>128	8	16	16	16	16	1
HS_58	64	1	16	16	4	4	64	0.25	>128	>128	4	16	16	8	32	0.5
HS_59	1	1	2	4	1	1	64	0.5	0.25	1	2	8	4	2	4	0.5
HS_60	16	1	16	32	8	8	>128	0.25	>128	>128	4	32	32	8	32	1
HS_61	128	2	16	16	8	8	64	0.5	>128	>128	8	32	32	16	32	1
HS_62	64	2	16	16	4	8	16	0.5	>128	>128	8	32	32	8	32	0.5
HS_63	8	1	32	16	8	8	64	0.25	>128	>128	2	32	32	8	32	0.5
HS_64	1	1	4	4	1	2	128	0.5	0.25	0.5	4	4	4	2	4	1

HS_65	1	1	16	16	8	8	128	0.25	>128	>128	4	32	32	16	32	0.5
HS_66	0.5	1	16	16	2	4	64	0.25	>128	>128	2	16	16	4	16	0.5
HS_67	0.5	16	16	16	2	4	64	8	>128	>128	2	16	16	4	16	0.5
HS_68	1	1	1	2	0.5	1	64	0.25	0.13	0.5	2	2	2	2	2	1
HS_69	0.5	1	16	16	8	8	64	0.25	>128	>128	2	32	32	8	32	0.5
HS_70	8	1	16	32	8	8	64	0.25	>128	>128	2	32	32	8	32	0.5
HS_71	64	1	8	16	1	4	64	0.13	>128	>128	8	4	16	4	16	0.5
HS_72	1	1	16	32	4	8	64	0.25	>128	>128	4	16	16	8	16	0.5
HS_73	>128	1	64	128	1	8	32	0.5	2	8	8	4	16	16	64	0.5
HS_74	128	2	4	2	2	4	64	0.25	>128	>128	4	2	8	64	8	1
HS_75	1	1	32	16	8	8	64	2	1	4	4	32	32	16	32	1
HS_76	8	1	32	16	8	8	64	0.25	>128	>128	2	32	32	8	32	0.5
HS_77	1	1	8	2	1	1	8	0.25	0.25	0.5	2	4	8	8	4	1
HS_78	1	2	4	4	1	2	32	0.5	0.25	1	4	4	4	4	4	0.5
HS_79	1	1	8	16	1	1	64	0.13	>128	>128	4	4	16	2	8	1
HS_80	1	2	4	4	2	4	64	0.5	1	8	4	4	4	8	4	1
HS_81	64	1	32	32	8	8	64	0.25	>128	>128	4	32	32	8	32	1
HS_83	1	1	16	16	2	4	64	0.25	>128	>128	4	16	16	4	16	1
HS_84	1	1	16	16	2	4	64	0.25	>128	>128	4	16	16	4	16	1
HS_85	64	1	16	16	4	4	64	0.25	128	>128	4	16	16	8	16	1
HS_86	64	1	16	16	4	8	64	0.25	>128	>128	8	32	32	8	32	0.5
HS_87	1	1	2	2	0.5	0.5	64	<0.06		>128	4	1	2	2	4	1
HS_88	1	1	1	2	0.5	0.5	32	<0.06	0.25	0.5	4	1	2	2	2	1
HS_89	64	1	16	16	8	8	64	0.5	>128	>128	8	32	32	16	32	1
HS_90	64	1	32	16	8	8	64	0.25	>128	>128	4	32	32	8	32	1
HS_91	64	1	16	16	8	8	64	0.25	>128	>128	4	32	32	8	32	1
HS_92	64	1	16	16	4	8	64	0.25	>128	>128	4	32	32	8	32	1
HS_93	8	1	16	16	4	4	32	0.5	>128	>128	2	16	16	8	16	1
HS_94	64	1	8	4	2	4	64	0.13	64	>128	8	4	8	4	4	1
HS_95	8	1	32	32	8	8	64	0.25	>128	>128	2	32	32	8	32	1
HS_96	>128	0.5	32	32	4	8	64	0.25	>128	>128	16	32	32	16	32	1
HS_97	2	0.5	16	16	8	4	128	0.13	>128	>128	2	32	32	8	32	0.5

HS_99	2	8	16	16	2	2	64	8	>128	>128	2	8	16	2	16	0.5
HS_100	2	8	16	16	2	2	64	8	>128	>128	2	16	16	2	16	1
HS_101	4	1	16	16	2	4	64	0.25	>128	>128	4	16	16	4	16	0.5
HS_102	128	1	16	32	1	2	64	0.13	>128	>128	8	8	16	4	16	0.5
HS_103	2	1	16	32	4	4	64	0.25	>128	>128	2	32	32	8	32	1
HS_104	4	1	8	8	1	2	64	0.13	64	128	4	16	8	1	16	0.5
HS_105	128	1	16	32	4	4	64	0.25	>128	>128	8	32	32	8	32	0.5
HS_106	16	1	16	16	4	4	64	0.25	>128	>128	2	32	32	8	32	1
HS_107	32	0.5	16	16	4	4	64	0.25	>128	>128	4	16	16	4	16	1
HS_109	4	2	2	4	0.5	0.5	64	0.13	>128	>128	8	1	2	2	4	0.5
HS_110	4	1	2	4	0.5	1	32	0.5	1	2	4	32	4	4	8	0.5
HS_111	32	1	16	16	4	4	64	0.5	>128	>128	4	16	32	4	32	0.5
HS_112	32	1	16	32	1	1	64	0.13	>128	>128	4	8	16	2	32	0.5
HS_113	64	1	16	32	4	4	64	0.25	>128	>128	4	32	32	8	32	0.13
HS_114	2	1	16	16	2	2	128	0.25	>128	>128	2	16	16	2	16	1
HS_115	64	1	16	16	4	4	64	0.25	>128	>128	4	32	16	4	32	1
HS_116	4	2	4	8	2	2	64	0.25	0.13	0.5	8	16	4	2	8	1
HS_117	2	1	16	16	4	4	64	0.25	>128	>128	2	32	32	8	32	1
HS_118	32	1	16	32	4	4	>128	0.25	>128	>128	2	32	16	4	32	1
HS_119	>128	1	16	32	4	4	128	0.25	>128	>128	16	32	32	8	32	1
HS_120	>128	0.5	32	64	2	2	>128	0.25	>128	>128	8	16	64	8	128	1
HS_121	2	8	16	16	2	2	64	8	>128	>128	2	16	16	2	16	1
HS_122	32	1	16	16	4	4	>128	0.25	>128	>128	4	32	16	4	32	1
HS_123	16	1	16	16	1	2	>128	0.25	>128	>128	2	16	16	2	16	1
HS_124	4	1	16	16	4	4	128	0.25	>128	>128	8	32	16	4	32	1
HS_125	16	1	16	16	4	4	128	0.25	>128	>128	2	32	32	4	32	1
HS_126	2	1	4	8	1	1	64	0.25	16	32	1	16	4	2	8	1
HS_127	2	1	16	16	2	2	64	0.25	>128	>128	2	16	16	2	32	1
HS_128	2	1	16	16	1	2	64	0.25	>128	>128	2	16	16	2	16	1
HS_130	64	1	16	32	4	4	>128	0.25	>128	>128	4	32	32	8	32	1
HS_131	8	1	16	16	4	4	128	0.5	>128	>128	8	32	32	8	32	1
HS_132	16	1	16	16	4	4	64	0.5	>128	>128	8	32	32	8	32	1

HS_133	16	1	16	16	4	4	64	0.25	>128	>128	2	16	16	4	32	2
HS_134	2	1	16	16	1	2	64	0.25	>128	>128	1	8	16	2	16	1
HS_135	64	1	16	16	4	4	64	0.25	>128	>128	4	16	32	4	32	1
HS_137	8	1	16	8	4	4	32	1	16	32	8	16	16	8	16	1
HS_138	32	1	16	16	8	8	64	0.25	>128	>128	4	32	32	8	32	1
HS_139	32	1	16	16	2	2	128	0.25	>128	>128	2	16	16	2	16	1
HS_140	32	1	16	8	4	4	>128	0.25	>128	>128	4	32	32	4	32	1
HS_141	2	1	16	8	4	4	128	0.25	>128	>128	2	16	16	4	16	0.5
HS_142	2	1	16	16	2	2	64	0.25	>128	>128	2	16	16	2	16	0.5
HS_143	2	0.5	16	32	1	2	64	0.25	>128	>128	2	16	16	2	32	0.5
HS_144	2	1	16	64	0.5	8	64	0.25	>128	>128	4	4	16	2	32	1
HS_145	2	2	2	2	1	1	>128	<0.06	0.13	0.5	4	2	4	1	4	1
HS_146	64	1	16	16	2	2	64	0.25	>128	>128	8	8	16	4	16	1
HS_147	128	2	16	16	4	4	128	0.25	>128	>128	8	16	16	8	16	1
HS_148	16	8	16	16	1	2	128	8	>128	>128	2	8	16	2	16	0.5
HS_149	4	1	4	8	1	2	64	0.13	0.13	0.25	4	8	4	2	8	2
HS_152	128	1	16	32	1	2	64	0.25	>128	>128	8	16	16	4	32	1
HS_153	32	1	2	4	1	1	128	<0.06	32	32	2	2	2	1	4	0.5
HS_154	2	0.5	2	4	1	1	64	<0.06	0.13	0.25	4	4	4	1	8	2
HS_155	32	0.5	16	16	2	2	64	0.13	>128	>128	2	16	16	4	16	1
HS_156	32	1	32	64	1	16	16	0.13	>128	>128	4	16	32	4	64	0.5
HS_157	>128	1	1	2	0.5	0.5	64	<0.06	>128	>128	8	1	2	4	4	1
HS_158	2	1	1	2	0.25	0.5	128	0.25	0.13	0.25	2	1	2	0.5	2	2
HS_159	2	1	16	16	1	2	64	0.25	>128	>128	2	8	16	2	16	1
HS_160	2	1	16	16	4	4	128	0.25	>128	>128	2	32	16	4	32	2
HS_161	1	1	4	8	1	2	8	0.25	0.13	0.25	4	8	4	1	8	1
HS_162	2	1	16	16	2	2	64	0.25	>128	>128	1	8	16	2	16	0.5
HS_163	4	1	1	2	0.25	0.5	8	0.13	0.25	0.5	4	1	1	1	2	0.5
HS_164	8	1	16	16	4	4	64	0.25	>128	>128	1	32	32	4	32	1
HS_165	4	2	4	4	1	1	32	2	32	64	8	4	4	4	8	0.5
HS_166	4	1	16	32	8	4	64	0.25	>128	>128	4	>128	16	4	32	1
HS_167	64	1	16	32	4	4	64	0.25	>128	>128	4	16	32	4	32	1

HS_168	32	0.5	16	16	2	2	64	<0.06	>128	>128	4	16	16	4	16	1
HS_169	64	1	2	4	0.5	0.5	64	0.13	>128	>128	4	1	4	0.5	4	0.5
HS_170	8	1	32	16	8	8	64	0.25	>128	>128	4	64	32	8	64	0.5
HS_171	>128	1	8	8	4	4	64	0.25	>128	>128	8	8	16	8	16	1
HS_172	>128	1	8	32	4	4	64	0.25	>128	>128	8	8	16	8	16	1
HS_173	128	1	16	16	0.5	8	64	0.13	>128	>128	8	8	16	4	32	1
HS_174	64	1	16	16	4	2	64	0.25	>128	>128	4	16	16	4	16	1
HS_175	32	1	16	16	4	4	128	0.25	>128	>128	2	32	32	4	32	1
HS_176	32	1	16	16	2	2	64	0.13	>128	>128	4	8	16	4	16	1
HS_177	32	1	16	32	4	4	64	0.13	>128	>128	4	16	16	4	16	1
HS_178	4	0.25	4	16	0.25	0.5	64	0.5	128	64	1	4	4	1	8	0.5
HS_179	8	1	16	8	4	4	128	0.25	128	>128	2	32	32	4	8	1
HS_180	>128	1	8	32	4	2	8	2	128	>128	>128	16	8	8	8	0.5
HS_181	16	1	16	16	4	8	64	0.25	128	>128	2	32	32	8	8	0.5
HS_182	32	1	16	16	4	4	>128	0.25	128	>128	4	32	32	8	8	0.5
HS_183	32	1	16	8	4	4	>128	0.25	128	>128	2	32	32	4	8	1
HS_184	4	0.5	4	4	1	1	128	0.13	0.13	0.5	4	4	2	1	4	0.5
HS_185	4	1	4	>128	1	1	64	0.5	0.13	0.25	4	4	4	1	4	0.5
HS_186	8	4	128	32	8	64	64	4	>128	>128	8	64	64	16	128	0.5
HS_187	16	1	16	16	4	4	128	0.25	>128	>128	2	32	32	4	32	1
HS_188	2	1	8	32	1	2	>128	0.25	0.13	0.25	4	8	8	2	8	1
HS_189	32	8	16	8	8	8	64	16	>128	>128	4	32	32	8	32	1
HS_190	128	1	8	32	2	2	64	0.13	>128	>128	8	8	8	8	16	0.5
HS_191	64	1	16	4	4	4	64	0.25	>128	>128	4	16	32	4	32	1
HS_192	4	1	4	2	1	1	64	0.25	0.25	0.5	4	4	4	1	4	0.5
HS_193	8	0.5	16	16	2	2	64	0.25	>128	>128	2	32	32	4	16	1
HS_194	1	0.5	4	4	2	2	64	0.5	0.13	0.5	4	16	8	2	4	1
HS_195	2	0.5	16	16	2	2	>128	0.13	>128	>128	4	16	32	2	16	1
HS_196	1	1	8	8	2	2	128	0.25	0.25	2	2	32	16	4	16	2
HS_197	16	1	16	16	2	2	>128	0.25	>128	>128	8	16	32	1	16	1
HS_198	32	0.13	16	16	2	2	>128	<0.06	>128	>128	32	32	32	4	16	2
HS_199	>128	32	64	>128	4	16	32	128	32	32	64	32	128	8	>128	1

HS_200	16	0.5	16	16	2	2	32	0.25	>128	>128	2	16	32	4	16	1
HS_201	2	0.5	8	8	1	1	64	0.13	>128	>128	1	8	16	1	8	1
HS_202	2	1	4	8	1	1	32	0.25	0.13	0.5	4	4	4	1	4	1
HS_203	1	0.25	4	8	1	2	32	0.13	<0.06	0.5	2	4	8	1	8	2
HS_204	2	0.5	4	4	1	1	64	0.13	8	16	4	4	8	1	4	2
HS_205	1	0.5	4	4	1	1	32	0.25	<0.06	0.5	2	4	4	1	4	1
HS_206	32	0.5	16	16	4	4	128	0.5	>128	>128	4	32	64	8	32	2
HS_207	1	1	4	4	1	2	32	0.13	<0.06	1	2	4	4	1	4	2

Tab. S3: Minimum inhibitory concentration values of 199 ear swab isolates reported in this study. GEN, Gentamicin; IMP, Imipenem; TZP, Piperacillin-tazobactam; PIP, Piperacillin; CZA, Ceftazidime-avibactam; CAZ, Ceftazidime; FOS, Fosfomycin; MEM, Meropenem; CIP, Ciprofloxacin, LEV, Levofloxacin; AMK, Amikacin; ATM, Aztreonam; SCF, Cefoperazone-Sulbactam; CEF, Cefepime; CFP, Cefoperazone; POL B, Polymyxin B.

Gene	Description	Mutation type	COG_category
glyS	Glycyl-tRNA synthetase beta subunit	NSY	J
-	DSBA-like thioredoxin domain	NSY	Q
pilU	twitching motility protein	NSY	NU
coq7	Oxygenase that introduces the hydroxyl group at carbon five of 2-nonaprenyl-3-methyl-6-methoxy-1,4-benzoquinol resulting in the formation of 2-nonaprenyl-3-methyl-5-hydroxy-6-methoxy-1,4- benzoquinol	NSY	H
-	Belongs to the class I-like SAM-binding methyltransferase superfamily. C5-methyltransferase family	NSY	H
-	Major Facilitator Superfamily	NSY	EGP
-	polygalacturonase activity	NSY	M
atuE	Enoyl-CoA hydratase/isomerase	NSY	I
-	-	LOS	-
hasF	Outer membrane efflux protein	LOS	MU
-	PFAM coagulation factor 5 8 type domain protein	NSY	U
lpxB	Condensation of UDP-2,3-diacylglucosamine and 2,3- diacylglucosamine-1-phosphate to form lipid A disaccharide, a precursor of lipid A, a phosphorylated glycolipid that anchors the lipopolysaccharide to the outer membrane of the cell	NSY	M
cdsA	Belongs to the CDS family	NSY	I
purT	Involved in the de novo purine biosynthesis. Catalyzes the transfer of formate to 5-phospho-ribosyl-glycinamide (GAR), producing 5-phospho-ribosyl-N-formylglycinamide (FGAR). Formate is provided by PurU via hydrolysis of 10-formyl-tetrahydrofolate	NSY	F
smc	Required for chromosome condensation and partitioning	NSY	D
yjaB	Acetyltransferase (GNAT) domain	NSY	K
IV02_08980	Bacterial regulatory protein, Fis family	NSY	T
-	Belongs to the major facilitator superfamily	NSY	EGP
-	Domain of unknown function (DUF4880)	NSY	PT
-	Phosphonate ABC transporter phosphate-binding periplasmic protein	NSY	-
-	CHASE4 domain	NSY	T
-	Enoyl-CoA hydratase/isomerase	NSY	I
-	Peptidase dimerisation domain	LOS	S

-	4-hydroxy-3-methylbut-2-enyl diphosphate reductase	NSY	S
-	Protein of unknown function (DUF3426)	NSY	S

Tab. S4: Mutations that were shared in all ED isolates but missing in all non-ED genomes. NSY, nonsynonymous mutations; LOS, loss-of-function mutations (i.e., indels and premature stop). COG functional categories: D, cell cycle control and mitosis; E, amino acid metabolism and transport; F, nucleotide metabolism and transport; G, carbohydrate metabolism and transport; H, coenzyme metabolism; I, lipid metabolism; J, translation; K, transcription; M, Cell wall/membrane/envelop biogenesis; N, cell motility; P, inorganic ion transport and metabolism; Q, secondary structure; S, function unknown; T, signal transduction; U, intracellular trafficking and secretion.

Drug	Kmer ID	Reference Region (chromosome/plasmid)	Putatiive annotation	Additional annotation	Weight
Gentamicin	K56917	131403..131558 (plasmid)	*sul1*	qnrVC1 bearing plasmid MDR region	0.009599
	K20705	135938..137012 (plasmid)	*IS*91 family transposase	qnrVC1 bearing plasmid MDR region	0.009123
	K27240	132171..132362 (plasmid)	*floR*	qnrVC1 bearing plasmid MDR region	0.008872
	K53309	132114..132170 (plasmid)	*floR*	qnrVC1 bearing plasmid MDR region	0.008679
	K53298	131719..131774 (plasmid)	*floR*	qnrVC1 bearing plasmid MDR region	0.00831
	K53317	132144..132200 (plasmid)	*floR*	qnrVC1 bearing plasmid MDR region	0.008212
	K53284	131611..131648 (plasmid)		qnrVC1 bearing plasmid MDR region	0.007346
	K17876	137568..137627 (plasmid)		qnrVC1 bearing plasmid MDR region	0.007206
	K41263	131550..131581 (plasmid)		qnrVC1 bearing plasmid MDR region	0.007034
	K53242	131042..131102 (plasmid)	*sul1*	qnrVC1 bearing plasmid MDR region	0.006942
Fosfomycin	K46375	5444738..5444771 (chromosome)	conjugal transfer protein TraG N-terminal domain-containing protein		0.001961
	K46375	4661752..4661785 (chromosome)	conjugal transfer protein TraG N-terminal domain-containing protein		0.001961
	K51257	2888255..2888315 (chromosome)	protease modulator HflK		0.001636
	K17716	3385278..3385310 (chromosome)	MFS transporter		0.001605
	K31300	5421242..5421302 (chromosome)		Type IVa pilus pathogenicity island	0.001423
	K31300	5441097..5441157 (chromosome)		Type IVa pilus pathogenicity island	0.001423
	K31300	5431180..5431240 (chromosome)		Type IVa pilus pathogenicity island	0.001423
	K31300	4658111..4658171 (chromosome)			0.001423
	K35987	2240784..2240816 (chromosome)	*sbrR*, Anti-sigma factor SbrR		0.001307
Cefoperazone-Sulbactam	K1103	130023..130053 (plasmid)		qnrVC1 bearing plasmid MDR region	0.001546
	K29977	124909..125899 (plasmid)	*int1*	qnrVC1 bearing plasmid MDR region	0.001477
	K1105	130026..130056 (plasmid)		qnrVC1 bearing plasmid MDR region	0.001467

K45074	2944912..2945031 (chromosome)	*pvdI*	Pyoverdine (Virulence gene)	0.001456
K46742	1954668..1954706 (chromosome)			0.001252
K17289	2842562..2842622 (chromosome)	*pvdI*	Pyoverdine (Virulence gene)	0.001209
K51453	2944867..2944927 (chromosome)	*pvdI*	Pyoverdine (Virulence gene)	0.001183
K51453	2937286..2937346 (chromosome)	*pvdI*	Pyoverdine (Virulence gene)	0.001183
K49796	2944902..2944941 (chromosome)	*pvdI*	Pyoverdine (Virulence gene)	0.001159
K45100	2937286..2937329 (chromosome)	*pvdI*	Pyoverdine (Virulence gene)	0.001142
K45100	2944867..2944910 (chromosome)	*pvdI*	Pyoverdine (Virulence gene)	0.001142

Tab. S5: k-mers most predictive (top 10) of resistance in the random forest model.

Cluster	Non-unique Gene name	Annotation	Number_pos_present_in	Number_neg_present_in	Number_pos_not_present_in	Number_neg_not_present_in	Sensitivity	Specificity	Odds_ratio	Naive_p	Bonferroni_p	Benjamini_H_p
group_9923		hypothetical protein	147	0	0	52	100	100	inf	3.53E-49	5.26E-45	1.46E-46
group_9743		hypothetical protein	147	0	0	52	100	100	inf	3.53E-49	5.26E-45	1.46E-46
group_9431		hypothetical protein	147	0	0	52	100	100	inf	3.53E-49	5.26E-45	1.46E-46
group_9400		hypothetical protein	147	0	0	52	100	100	inf	3.53E-49	5.26E-45	1.46E-46
group_9351		hypothetical protein	147	0	0	52	100	100	inf	3.53E-49	5.26E-45	1.46E-46
group_9058		hypothetical protein	147	0	0	52	100	100	inf	3.53E-49	5.26E-45	1.46E-46
group_8991		hypothetical protein	147	0	0	52	100	100	inf	3.53E-49	5.26E-45	1.46E-46
group_8513		hypothetical protein	147	0	0	52	100	100	inf	3.53E-49	5.26E-45	1.46E-46
group_8286		hypothetical protein	147	0	0	52	100	100	inf	3.53E-49	5.26E-45	1.46E-46
group_8220		hypothetical protein	147	0	0	52	100	100	inf	3.53E-49	5.26E-45	1.46E-46

group_8007		hypothetical protein	147	0	0	52	100	100	inf	3.53 E-49	5.26E-45	1.46E-46
group_7902		hypothetical protein	147	0	0	52	100	100	inf	3.53 E-49	5.26E-45	1.46E-46
group_7901		hypothetical protein	147	0	0	52	100	100	inf	3.53 E-49	5.26E-45	1.46E-46
group_7651		hypothetical protein	147	0	0	52	100	100	inf	3.53 E-49	5.26E-45	1.46E-46
group_7298		hypothetical protein	147	0	0	52	100	100	inf	3.53 E-49	5.26E-45	1.46E-46
group_6931		hypothetical protein	147	0	0	52	100	100	inf	3.53 E-49	5.26E-45	1.46E-46
group_5952		hypothetical protein	147	0	0	52	100	100	inf	3.53 E-49	5.26E-45	1.46E-46
group_5645		hypothetical protein	147	0	0	52	100	100	inf	3.53 E-49	5.26E-45	1.46E-46
group_5561		hypothetical protein	147	0	0	52	100	100	inf	3.53 E-49	5.26E-45	1.46E-46
group_5451		hypothetical protein	147	0	0	52	100	100	inf	3.53 E-49	5.26E-45	1.46E-46
group_5324	soj_2	Sporulation initiation inhibitor protein Soj	147	0	0	52	100	100	inf	3.53 E-49	5.26E-45	1.46E-46
group_5074		hypothetical protein	147	0	0	52	100	100	inf	3.53 E-49	5.26E-45	1.46E-46
group_3523		hypothetical protein	147	0	0	52	100	100	inf	3.53 E-49	5.26E-45	1.46E-46
group_3370		hypothetical protein	147	0	0	52	100	100	inf	3.53 E-49	5.26E-45	1.46E-46
group_3186		putative HTH-type	147	0	0	52	100	100	inf	3.53 E-49	5.26E-45	1.46E-46

		transcriptional regulator										
group_2992		hypothetical protein	147	0	0	52	100	100	inf	3.53 E-49	5.26E-45	1.46E-46
group_2713		hypothetical protein	147	0	0	52	100	100	inf	3.53 E-49	5.26E-45	1.46E-46
group_2588		hypothetical protein	147	0	0	52	100	100	inf	3.53 E-49	5.26E-45	1.46E-46
group_2047	hsdS	Type-1 restriction enzyme EcoKI specificity protein	147	0	0	52	100	100	inf	3.53 E-49	5.26E-45	1.46E-46
group_1962		hypothetical protein	147	0	0	52	100	100	inf	3.53 E-49	5.26E-45	1.46E-46
group_1951	hsdM	Type I restriction enzyme EcoKI M protein	147	0	0	52	100	100	inf	3.53 E-49	5.26E-45	1.46E-46
group_1651		hypothetical protein	147	0	0	52	100	100	inf	3.53 E-49	5.26E-45	1.46E-46
group_1121		hypothetical protein	147	0	0	52	100	100	inf	3.53 E-49	5.26E-45	1.46E-46
group_347		hypothetical protein	147	0	0	52	100	100	inf	3.53 E-49	5.26E-45	1.46E-46
group_211		hypothetical protein	147	0	0	52	100	100	inf	3.53 E-49	5.26E-45	1.46E-46
group_160		hypothetical protein	147	0	0	52	100	100	inf	3.53 E-49	5.26E-45	1.46E-46
group_10717		hypothetical protein	147	1	0	51	100	98	inf	5.23 E-47	7.78E-43	1.20E-44

group_10228	pilA	Fimbrial protein	147	1	0	51	100	98	inf	5.23 E-47	7.78E-43	1.20E-44
group_10190		hypothetical protein	147	1	0	51	100	98	inf	5.23 E-47	7.78E-43	1.20E-44
group_10131		hypothetical protein	147	1	0	51	100	98	inf	5.23 E-47	7.78E-43	1.20E-44
group_9911		hypothetical protein	147	1	0	51	100	98	inf	5.23 E-47	7.78E-43	1.20E-44
group_9383		hypothetical protein	147	1	0	51	100	98	inf	5.23 E-47	7.78E-43	1.20E-44
recD2_3~~~recD2_2	recD2_3;recD2_2	ATP-dependent RecD-like DNA helicase	147	1	0	51	100	98	inf	5.23 E-47	7.78E-43	1.20E-44
group_8966		hypothetical protein	147	1	0	51	100	98	inf	5.23 E-47	7.78E-43	1.20E-44
group_7830		hypothetical protein	147	1	0	51	100	98	inf	5.23 E-47	7.78E-43	1.20E-44
group_6047		hypothetical protein	147	1	0	51	100	98	inf	5.23 E-47	7.78E-43	1.20E-44
group_4659		hypothetical protein	147	1	0	51	100	98	inf	5.23 E-47	7.78E-43	1.20E-44
group_3721		hypothetical protein	147	1	0	51	100	98	inf	5.23 E-47	7.78E-43	1.20E-44
group_3166	xerD_1	Tyrosine recombinase XerD	147	1	0	51	100	98	inf	5.23 E-47	7.78E-43	1.20E-44
group_2980		hypothetical protein	147	1	0	51	100	98	inf	5.23 E-47	7.78E-43	1.20E-44
group_2486	intS_2	Prophage integrase IntS	147	1	0	51	100	98	inf	5.23 E-47	7.78E-43	1.20E-44
group_2059		hypothetical protein	147	1	0	51	100	98	inf	5.23 E-47	7.78E-43	1.20E-44

group_1910	hypothetical protein	147	1	0	51	100	98	inf	5.23 E-47	7.78E-43	1.20E-44
group_1643	hypothetical protein	147	1	0	51	100	98	inf	5.23 E-47	7.78E-43	1.20E-44
group_1532	hypothetical protein	147	1	0	51	100	98	inf	5.23 E-47	7.78E-43	1.20E-44
group_1321	hypothetical protein	147	1	0	51	100	98	inf	5.23 E-47	7.78E-43	1.20E-44
group_1238	hypothetical protein	147	1	0	51	100	98	inf	5.23 E-47	7.78E-43	1.20E-44
group_1159	hypothetical protein	147	1	0	51	100	98	inf	5.23 E-47	7.78E-43	1.20E-44
group_1092	hypothetical protein	147	1	0	51	100	98	inf	5.23 E-47	7.78E-43	1.20E-44
group_1014	hypothetical protein	147	1	0	51	100	98	inf	5.23 E-47	7.78E-43	1.20E-44
group_773	hypothetical protein	147	1	0	51	100	98	inf	5.23 E-47	7.78E-43	1.20E-44
group_727	hypothetical protein	147	1	0	51	100	98	inf	5.23 E-47	7.78E-43	1.20E-44
group_599	hypothetical protein	147	1	0	51	100	98	inf	5.23 E-47	7.78E-43	1.20E-44
group_277	hypothetical protein	147	1	0	51	100	98	inf	5.23 E-47	7.78E-43	1.20E-44
group_188	hypothetical protein	147	1	0	51	100	98	inf	5.23 E-47	7.78E-43	1.20E-44
group_8682	hypothetical protein	145	0	2	52	99	100	inf	5.06 E-46	7.52E-42	9.90E-44
group_7730	hypothetical protein	145	0	2	52	99	100	inf	5.06 E-46	7.52E-42	9.90E-44
group_4704	hypothetical protein	145	0	2	52	99	100	inf	5.06 E-46	7.52E-42	9.90E-44

group_4380		hypothetical protein	145	0	2	52	99	100	inf	5.06 E-46	7.52E-42	9.90E-44
group_3843		hypothetical protein	145	0	2	52	99	100	inf	5.06 E-46	7.52E-42	9.90E-44
group_3052		hypothetical protein	145	0	2	52	99	100	inf	5.06 E-46	7.52E-42	9.90E-44
group_2534	intA_2	Prophage integrase IntA	145	0	2	52	99	100	inf	5.06 E-46	7.52E-42	9.90E-44
group_2387		hypothetical protein	145	0	2	52	99	100	inf	5.06 E-46	7.52E-42	9.90E-44
group_1986		hypothetical protein	145	0	2	52	99	100	inf	5.06 E-46	7.52E-42	9.90E-44
rep_4	rep_4	ATP-dependent DNA helicase Rep	145	0	2	52	99	100	inf	5.06 E-46	7.52E-42	9.90E-44
recF_3	recF_3	DNA replication and repair protein RecF	145	0	2	52	99	100	inf	5.06 E-46	7.52E-42	9.90E-44
group_1657		hypothetical protein	146	1	1	51	99	98	##	2.70 E-45	4.02E-41	5.22E-43
group_9910	;czcC_1	hypothetical protein;Cobalt-zinc-cadmium resistance protein CzcC	147	2	0	50	100	96	inf	3.90 E-45	5.80E-41	6.51E-43
group_9725		hypothetical protein	147	2	0	50	100	96	inf	3.90 E-45	5.80E-41	6.51E-43
group_8923		hypothetical protein	147	2	0	50	100	96	inf	3.90 E-45	5.80E-41	6.51E-43

group_8150		hypothetical protein	147	2	0	50	100	96	inf	3.90 E-45	5.80E-41	6.51E-43
cusF	cusF	Cation efflux system protein CusF	147	2	0	50	100	96	inf	3.90 E-45	5.80E-41	6.51E-43
group_5191		hypothetical protein	147	2	0	50	100	96	inf	3.90 E-45	5.80E-41	6.51E-43
group_4694		hypothetical protein	147	2	0	50	100	96	inf	3.90 E-45	5.80E-41	6.51E-43
group_1213		hypothetical protein	147	2	0	50	100	96	inf	3.90 E-45	5.80E-41	6.51E-43
group_1184		hypothetical protein	147	2	0	50	100	96	inf	3.90 E-45	5.80E-41	6.51E-43
group_1140		hypothetical protein	147	2	0	50	100	96	inf	3.90 E-45	5.80E-41	6.51E-43
group_293	cusA	Cation efflux system protein CusA	147	2	0	50	100	96	inf	3.90 E-45	5.80E-41	6.51E-43
group_99		hypothetical protein	147	2	0	50	100	96	inf	3.90 E-45	5.80E-41	6.51E-43
group_6758		hypothetical protein	144	0	3	52	98	100	inf	9.27 E-45	1.38E-40	1.48E-42
group_4815		hypothetical protein	144	0	3	52	98	100	inf	9.27 E-45	1.38E-40	1.48E-42
group_4811		hypothetical protein	144	0	3	52	98	100	inf	9.27 E-45	1.38E-40	1.48E-42
group_1524		hypothetical protein	144	0	3	52	98	100	inf	9.27 E-45	1.38E-40	1.48E-42
group_10162		hypothetical protein	145	1	2	51	99	98	##	7.11 E-44	1.06E-39	1.07E-41
group_1266		putative type I restriction	145	1	2	51	99	98	##	7.11 E-44	1.06E-39	1.07E-41

		enzymeP M protein										
group_1206		hypothetical protein	145	1	2	51	99	98	##	7.11 E-44	1.06E-39	1.07E-41
group_518	smc_1	Chromosome partition protein Smc	145	1	2	51	99	98	##	7.11 E-44	1.06E-39	1.07E-41
group_359	hsdR_1	Type-1 restriction enzyme R protein	145	1	2	51	99	98	##	7.11 E-44	1.06E-39	1.07E-41
group_165		hypothetical protein	145	1	2	51	99	98	##	7.11 E-44	1.06E-39	1.07E-41
group_8954		hypothetical protein	143	0	4	52	97	100	inf	1.30 E-43	1.93E-39	1.87E-41
group_3468		hypothetical protein	143	0	4	52	97	100	inf	1.30 E-43	1.93E-39	1.87E-41
group_1747		hypothetical protein	143	0	4	52	97	100	inf	1.30 E-43	1.93E-39	1.87E-41
group_1531		hypothetical protein	143	0	4	52	97	100	inf	1.30 E-43	1.93E-39	1.87E-41
group_9811		hypothetical protein	147	3	0	49	100	94	inf	1.95 E-43	2.90E-39	2.60E-41
group_8200		hypothetical protein	147	3	0	49	100	94	inf	1.95 E-43	2.90E-39	2.60E-41
group_7821		hypothetical protein	147	3	0	49	100	94	inf	1.95 E-43	2.90E-39	2.60E-41
group_7463		hypothetical protein	147	3	0	49	100	94	inf	1.95 E-43	2.90E-39	2.60E-41
group_2779		hypothetical protein	147	3	0	49	100	94	inf	1.95 E-43	2.90E-39	2.60E-41
group_1223		hypothetical protein	147	3	0	49	100	94	inf	1.95 E-43	2.90E-39	2.60E-41

rep_3~~~rep_4	rep_3;;rep_4	ATP-dependent DNA helicase Rep;hypothetical protein	147	3	0	49	100	94	inf	1.95 E-43	2.90E-39	2.60E-41
group_8782	cusB	Cation efflux system protein CusB	146	2	1	50	99	96	##	1.96 E-43	2.92E-39	2.60E-41
group_1695		hypothetical protein	146	2	1	50	99	96	##	1.96 E-43	2.92E-39	2.60E-41
group_1667		hypothetical protein	144	1	3	51	98	98	##	1.27 E-42	1.89E-38	1.60E-40
group_1659		hypothetical protein	144	1	3	51	98	98	##	1.27 E-42	1.89E-38	1.60E-40
group_1658		hypothetical protein	144	1	3	51	98	98	##	1.27 E-42	1.89E-38	1.60E-40
group_1543		hypothetical protein	144	1	3	51	98	98	##	1.27 E-42	1.89E-38	1.60E-40
group_1540		hypothetical protein	144	1	3	51	98	98	##	1.27 E-42	1.89E-38	1.60E-40
rdgC_1	rdgC_1	Recombination-associated protein RdgC	144	1	3	51	98	98	##	1.27 E-42	1.89E-38	1.60E-40
group_8765		hypothetical protein	142	0	5	52	97	100	inf	1.48 E-42	2.20E-38	1.85E-40
group_1089		hypothetical protein	145	2	2	50	99	96	##	5.03 E-42	7.48E-38	5.94E-40
sutR_1	sutR_1	HTH-type transcriptional regulator SutR	145	2	2	50	99	96	##	5.03 E-42	7.48E-38	5.94E-40
lon_2~~~lon_3~~~lon_4	lon_2;lon_3;lon_4	Lon protease	145	2	2	50	99	96	##	5.03 E-42	7.48E-38	5.94E-40

group_1002		hypothetical protein	145	2	2	50	99	96	##	5.03 E-42	7.48E-38	5.94E-40
group_979		hypothetical protein	145	2	2	50	99	96	##	5.03 E-42	7.48E-38	5.94E-40
group_928		hypothetical protein	145	2	2	50	99	96	##	5.03 E-42	7.48E-38	5.94E-40
group_775		hypothetical protein	145	2	2	50	99	96	##	5.03 E-42	7.48E-38	5.94E-40
group_10353		hypothetical protein	147	4	0	48	100	92	inf	7.35 E-42	1.09E-37	7.87E-40
group_9907	lexA_1;	LexA repressor;putative HTH-type transcriptional regulator	147	4	0	48	100	92	inf	7.35 E-42	1.09E-37	7.87E-40
group_8905		hypothetical protein	147	4	0	48	100	92	inf	7.35 E-42	1.09E-37	7.87E-40
group_8040		hypothetical protein	147	4	0	48	100	92	inf	7.35 E-42	1.09E-37	7.87E-40
group_3942		IS3 family transposase ISPsy29	147	4	0	48	100	92	inf	7.35 E-42	1.09E-37	7.87E-40
group_3326		hypothetical protein	147	4	0	48	100	92	inf	7.35 E-42	1.09E-37	7.87E-40
intA~~~intA_1~~~intA_2	intA;intA_1;intA_2	Prophage integrase IntA	147	4	0	48	100	92	inf	7.35 E-42	1.09E-37	7.87E-40
group_63		hypothetical protein	147	4	0	48	100	92	inf	7.35 E-42	1.09E-37	7.87E-40
group_43		hypothetical protein	147	4	0	48	100	92	inf	7.35 E-42	1.09E-37	7.87E-40

group_9956		hypothetical protein	0	48	147	4	0	7.7	0	7.35 E-42	1.09E-37	7.87E-40
group_8885		hypothetical protein	0	48	147	4	0	7.7	0	7.35 E-42	1.09E-37	7.87E-40
group_386		hypothetical protein	0	48	147	4	0	7.7	0	7.35 E-42	1.09E-37	7.87E-40
group_305		hypothetical protein	0	48	147	4	0	7.7	0	7.35 E-42	1.09E-37	7.87E-40
aplIM	;aplIM	hypothetical protein;Modification methylase AplI	146	3	1	49	99	94	##	9.55 E-42	1.42E-37	9.66E-40
group_1697		hypothetical protein	146	3	1	49	99	94	##	9.55 E-42	1.42E-37	9.66E-40
group_1681		hypothetical protein	146	3	1	49	99	94	##	9.55 E-42	1.42E-37	9.66E-40
group_1678		hypothetical protein	146	3	1	49	99	94	##	9.55 E-42	1.42E-37	9.66E-40
xre_1	xre_1	HTH-type transcriptional regulator Xre	146	3	1	49	99	94	##	9.55 E-42	1.42E-37	9.66E-40
group_532		hypothetical protein	146	3	1	49	99	94	##	9.55 E-42	1.42E-37	9.66E-40
mutL_1~~~mutL_2	;mutL_1;mutL_2	hypothetical protein;DNA mismatch repair protein MutL	146	3	1	49	99	94	##	9.55 E-42	1.42E-37	9.66E-40
group_366		hypothetical protein	146	3	1	49	99	94	##	9.55 E-42	1.42E-37	9.66E-40

group_9757		hypothetical protein	141	0	6	52	96	100	inf	1.43E-41	2.13E-37	1.30E-39
group_9090		hypothetical protein	141	0	6	52	96	100	inf	1.43E-41	2.13E-37	1.30E-39
group_8730		hypothetical protein	141	0	6	52	96	100	inf	1.43E-41	2.13E-37	1.30E-39
group_8029		hypothetical protein	141	0	6	52	96	100	inf	1.43E-41	2.13E-37	1.30E-39
group_7972		hypothetical protein	141	0	6	52	96	100	inf	1.43E-41	2.13E-37	1.30E-39
group_7755		hypothetical protein	141	0	6	52	96	100	inf	1.43E-41	2.13E-37	1.30E-39
group_7335		hypothetical protein	141	0	6	52	96	100	inf	1.43E-41	2.13E-37	1.30E-39
group_6106		hypothetical protein	141	0	6	52	96	100	inf	1.43E-41	2.13E-37	1.30E-39
group_5802		hypothetical protein	141	0	6	52	96	100	inf	1.43E-41	2.13E-37	1.30E-39
group_4674		hypothetical protein	141	0	6	52	96	100	inf	1.43E-41	2.13E-37	1.30E-39
group_4068		hypothetical protein	141	0	6	52	96	100	inf	1.43E-41	2.13E-37	1.30E-39
exoX	exoX	Exodeoxyribonuclease 10	141	0	6	52	96	100	inf	1.43E-41	2.13E-37	1.30E-39
group_3842		hypothetical protein	141	0	6	52	96	100	inf	1.43E-41	2.13E-37	1.30E-39
tus	tus	DNA replication terminus site-binding protein	141	0	6	52	96	100	inf	1.43E-41	2.13E-37	1.30E-39
group_3235		hypothetical protein	141	0	6	52	96	100	inf	1.43E-41	2.13E-37	1.30E-39

group_2489	ltrA	Group II intron-encoded protein LtrA	141	0	6	52	96	100	inf	1.43 E-41	2.13E-37	1.30E-39
umuC_2	umuC_2	Protein UmuC	141	0	6	52	96	100	inf	1.43 E-41	2.13E-37	1.30E-39
group_4363		hypothetical protein	143	1	4	51	97	98	##	1.74 E-41	2.58E-37	1.57E-39
group_1664		hypothetical protein	144	2	3	50	98	96	##	8.77 E-41	1.30E-36	7.37E-39
group_1631		hypothetical protein	144	2	3	50	98	96	##	8.77 E-41	1.30E-36	7.37E-39
group_1629		hypothetical protein	144	2	3	50	98	96	##	8.77 E-41	1.30E-36	7.37E-39
group_1628		hypothetical protein	144	2	3	50	98	96	##	8.77 E-41	1.30E-36	7.37E-39
prtR_2~~~prtR_3	prtR_2;prtR_3	HTH-type transcriptional regulator PrtR	144	2	3	50	98	96	##	8.77 E-41	1.30E-36	7.37E-39
group_1617		hypothetical protein	144	2	3	50	98	96	##	8.77 E-41	1.30E-36	7.37E-39
group_1541		hypothetical protein	144	2	3	50	98	96	##	8.77 E-41	1.30E-36	7.37E-39
intS_3	intS_3	Prophage integrase IntS	144	2	3	50	98	96	##	8.77 E-41	1.30E-36	7.37E-39
torI	torI	Response regulator inhibitor for tor operon	144	2	3	50	98	96	##	8.77 E-41	1.30E-36	7.37E-39
group_1538		hypothetical protein	144	2	3	50	98	96	##	8.77 E-41	1.30E-36	7.37E-39

lexA_2	lexA_2	LexA repressor	144	2	3	50	98	96	##	8.77E-41	1.30E-36	7.37E-39
group_1526		hypothetical protein	144	2	3	50	98	96	##	8.77E-41	1.30E-36	7.37E-39
group_7395		hypothetical protein	147	5	0	47	100	90	inf	2.24E-40	3.33E-36	1.84E-38
group_5620		hypothetical protein	147	5	0	47	100	90	inf	2.24E-40	3.33E-36	1.84E-38
group_1212		hypothetical protein;IS66 family transposase ISPre3	147	5	0	47	100	90	inf	2.24E-40	3.33E-36	1.84E-38
group_106		hypothetical protein	147	5	0	47	100	90	inf	2.24E-40	3.33E-36	1.84E-38
group_10122		hypothetical protein	146	4	1	48	99	92	##	3.51E-40	5.22E-36	2.85E-38
group_531		hypothetical protein	146	4	1	48	99	92	##	3.51E-40	5.22E-36	2.85E-38
group_10526		hypothetical protein	141	1	6	51	96	98	##	1.82E-39	2.71E-35	1.09E-37
group_10373		hypothetical protein	141	1	6	51	96	98	##	1.82E-39	2.71E-35	1.09E-37
group_10100		hypothetical protein	141	1	6	51	96	98	##	1.82E-39	2.71E-35	1.09E-37
group_9898		hypothetical protein	141	1	6	51	96	98	##	1.82E-39	2.71E-35	1.09E-37
umuC~~~umuC_1	umuC;umuC_1	Protein UmuC	141	1	6	51	96	98	##	1.82E-39	2.71E-35	1.09E-37
group_9708		hypothetical protein	141	1	6	51	96	98	##	1.82E-39	2.71E-35	1.09E-37
group_9701		hypothetical protein	141	1	6	51	96	98	##	1.82E-39	2.71E-35	1.09E-37

group_9500		hypothetical protein	141	1	6	51	96	98	##	1.82 E-39	2.71E-35	1.09E-37
group_9397		hypothetical protein	141	1	6	51	96	98	##	1.82 E-39	2.71E-35	1.09E-37
group_9267		hypothetical protein	141	1	6	51	96	98	##	1.82 E-39	2.71E-35	1.09E-37
group_9069	clsB;	Cardiolipin synthase B;hypothetical protein	141	1	6	51	96	98	##	1.82 E-39	2.71E-35	1.09E-37
group_8973		hypothetical protein	141	1	6	51	96	98	##	1.82 E-39	2.71E-35	1.09E-37
group_8801		hypothetical protein	141	1	6	51	96	98	##	1.82 E-39	2.71E-35	1.09E-37
group_8795		hypothetical protein	141	1	6	51	96	98	##	1.82 E-39	2.71E-35	1.09E-37
group_8346		hypothetical protein	141	1	6	51	96	98	##	1.82 E-39	2.71E-35	1.09E-37
group_8297		hypothetical protein	141	1	6	51	96	98	##	1.82 E-39	2.71E-35	1.09E-37
group_7810		hypothetical protein	141	1	6	51	96	98	##	1.82 E-39	2.71E-35	1.09E-37
group_7765		hypothetical protein	141	1	6	51	96	98	##	1.82 E-39	2.71E-35	1.09E-37
group_7714		hypothetical protein	141	1	6	51	96	98	##	1.82 E-39	2.71E-35	1.09E-37
group_7691		hypothetical protein	141	1	6	51	96	98	##	1.82 E-39	2.71E-35	1.09E-37
group_7625		hypothetical protein	141	1	6	51	96	98	##	1.82 E-39	2.71E-35	1.09E-37
group_7624		hypothetical protein	141	1	6	51	96	98	##	1.82 E-39	2.71E-35	1.09E-37

group_7400		hypothetical protein	141	1	6	51	96	98	##	1.82 E-39	2.71E-35	1.09E-37
group_7243		hypothetical protein	141	1	6	51	96	98	##	1.82 E-39	2.71E-35	1.09E-37
group_7233		hypothetical protein	141	1	6	51	96	98	##	1.82 E-39	2.71E-35	1.09E-37
group_7157		hypothetical protein	141	1	6	51	96	98	##	1.82 E-39	2.71E-35	1.09E-37
group_7056		hypothetical protein	141	1	6	51	96	98	##	1.82 E-39	2.71E-35	1.09E-37
group_7030		hypothetical protein	141	1	6	51	96	98	##	1.82 E-39	2.71E-35	1.09E-37
group_7024		hypothetical protein	141	1	6	51	96	98	##	1.82 E-39	2.71E-35	1.09E-37
group_6897		hypothetical protein	141	1	6	51	96	98	##	1.82 E-39	2.71E-35	1.09E-37
group_6783		hypothetical protein	141	1	6	51	96	98	##	1.82 E-39	2.71E-35	1.09E-37
group_6771		hypothetical protein	141	1	6	51	96	98	##	1.82 E-39	2.71E-35	1.09E-37
hfq_2	hfq_2	RNA-binding protein Hfq	141	1	6	51	96	98	##	1.82 E-39	2.71E-35	1.09E-37
group_6575		hypothetical protein	141	1	6	51	96	98	##	1.82 E-39	2.71E-35	1.09E-37
group_6514		hypothetical protein	141	1	6	51	96	98	##	1.82 E-39	2.71E-35	1.09E-37
relA_2	relA_2	GTP pyrophospho kinase	141	1	6	51	96	98	##	1.82 E-39	2.71E-35	1.09E-37
group_6437		hypothetical protein	141	1	6	51	96	98	##	1.82 E-39	2.71E-35	1.09E-37
group_6436		hypothetical protein	141	1	6	51	96	98	##	1.82 E-39	2.71E-35	1.09E-37

group_6204	hypothetical protein	141	1	6	51	96	98	##	1.82E-39	2.71E-35	1.09E-37
group_6194	hypothetical protein	141	1	6	51	96	98	##	1.82E-39	2.71E-35	1.09E-37
group_6183	hypothetical protein	141	1	6	51	96	98	##	1.82E-39	2.71E-35	1.09E-37
group_6001	hypothetical protein	141	1	6	51	96	98	##	1.82E-39	2.71E-35	1.09E-37
group_5868	hypothetical protein	141	1	6	51	96	98	##	1.82E-39	2.71E-35	1.09E-37
group_5765	hypothetical protein	141	1	6	51	96	98	##	1.82E-39	2.71E-35	1.09E-37
group_5503	hypothetical protein	141	1	6	51	96	98	##	1.82E-39	2.71E-35	1.09E-37
group_5302	hypothetical protein	141	1	6	51	96	98	##	1.82E-39	2.71E-35	1.09E-37
group_5249	hypothetical protein	141	1	6	51	96	98	##	1.82E-39	2.71E-35	1.09E-37
group_5025	hypothetical protein	141	1	6	51	96	98	##	1.82E-39	2.71E-35	1.09E-37
group_4728	hypothetical protein	141	1	6	51	96	98	##	1.82E-39	2.71E-35	1.09E-37
group_4726	hypothetical protein	141	1	6	51	96	98	##	1.82E-39	2.71E-35	1.09E-37
group_4560	hypothetical protein	141	1	6	51	96	98	##	1.82E-39	2.71E-35	1.09E-37
group_4419	hypothetical protein	141	1	6	51	96	98	##	1.82E-39	2.71E-35	1.09E-37
group_4401	hypothetical protein	141	1	6	51	96	98	##	1.82E-39	2.71E-35	1.09E-37
group_4336	hypothetical protein	141	1	6	51	96	98	##	1.82E-39	2.71E-35	1.09E-37

group_4000		hypothetical protein	141	1	6	51	96	98	##	1.82 E-39	2.71E-35	1.09E-37
group_3596		hypothetical protein	141	1	6	51	96	98	##	1.82 E-39	2.71E-35	1.09E-37
group_3419		hypothetical protein	141	1	6	51	96	98	##	1.82 E-39	2.71E-35	1.09E-37
group_3102	xerC_2	Tyrosine recombinase XerC	141	1	6	51	96	98	##	1.82 E-39	2.71E-35	1.09E-37
group_3101		hypothetical protein	141	1	6	51	96	98	##	1.82 E-39	2.71E-35	1.09E-37
pvuIIM	pvuIIM	Modification methylase PvuII	141	1	6	51	96	98	##	1.82 E-39	2.71E-35	1.09E-37
group_2381		hypothetical protein	141	1	6	51	96	98	##	1.82 E-39	2.71E-35	1.09E-37
group_1941		IS66 family transposase ISSal1	141	1	6	51	96	98	##	1.82 E-39	2.71E-35	1.09E-37
group_1913		hypothetical protein	141	1	6	51	96	98	##	1.82 E-39	2.71E-35	1.09E-37
umuC~~~umuC_3~~~umuC_4	umuC;umuC_3;umuC_4	Protein UmuC	141	1	6	51	96	98	##	1.82 E-39	2.71E-35	1.09E-37
group_377		hypothetical protein	141	1	6	51	96	98	##	1.82 E-39	2.71E-35	1.09E-37
group_5023		hypothetical protein	144	3	3	49	98	94	784	4.05 E-39	6.03E-35	2.15E-37
group_5020		hypothetical protein	144	3	3	49	98	94	784	4.05 E-39	6.03E-35	2.15E-37
group_5017		hypothetical protein	144	3	3	49	98	94	784	4.05 E-39	6.03E-35	2.15E-37
group_4799	ssb_1	Single-stranded	144	3	3	49	98	94	784	4.05 E-39	6.03E-35	2.15E-37

polC_1~~~polC_2	polC_1;polC_2	DNA-binding protein DNA polymerase III PolC-type	144	3	3	49	98	94	784	4.05E-39	6.03E-35	2.15E-37
group_4796		hypothetical protein	144	3	3	49	98	94	784	4.05E-39	6.03E-35	2.15E-37
group_4792		hypothetical protein	144	3	3	49	98	94	784	4.05E-39	6.03E-35	2.15E-37
group_4791		hypothetical protein	144	3	3	49	98	94	784	4.05E-39	6.03E-35	2.15E-37
group_4790		hypothetical protein	144	3	3	49	98	94	784	4.05E-39	6.03E-35	2.15E-37
group_4789		hypothetical protein	144	3	3	49	98	94	784	4.05E-39	6.03E-35	2.15E-37
group_4787		hypothetical protein	144	3	3	49	98	94	784	4.05E-39	6.03E-35	2.15E-37
group_1662		hypothetical protein	144	3	3	49	98	94	784	4.05E-39	6.03E-35	2.15E-37
group_1652		hypothetical protein	144	3	3	49	98	94	784	4.05E-39	6.03E-35	2.15E-37
group_1630		hypothetical protein	144	3	3	49	98	94	784	4.05E-39	6.03E-35	2.15E-37
group_1627		hypothetical protein	144	3	3	49	98	94	784	4.05E-39	6.03E-35	2.15E-37
group_1626		hypothetical protein	144	3	3	49	98	94	784	4.05E-39	6.03E-35	2.15E-37
group_1625		hypothetical protein	144	3	3	49	98	94	784	4.05E-39	6.03E-35	2.15E-37
group_1624		hypothetical protein	144	3	3	49	98	94	784	4.05E-39	6.03E-35	2.15E-37
group_1623		hypothetical protein	144	3	3	49	98	94	784	4.05E-39	6.03E-35	2.15E-37

group_1622		hypothetical protein	144	3	3	49	98	94	784	4.05 E-39	6.03E-35	2.15E-37
group_1620		hypothetical protein	144	3	3	49	98	94	784	4.05 E-39	6.03E-35	2.15E-37
group_1619		hypothetical protein	144	3	3	49	98	94	784	4.05 E-39	6.03E-35	2.15E-37
group_1618		hypothetical protein	144	3	3	49	98	94	784	4.05 E-39	6.03E-35	2.15E-37
csrA_2	csrA_2	Translational regulator CsrA	144	3	3	49	98	94	784	4.05 E-39	6.03E-35	2.15E-37
group_1613		hypothetical protein	144	3	3	49	98	94	784	4.05 E-39	6.03E-35	2.15E-37
group_1612		hypothetical protein	144	3	3	49	98	94	784	4.05 E-39	6.03E-35	2.15E-37
group_1611		hypothetical protein	144	3	3	49	98	94	784	4.05 E-39	6.03E-35	2.15E-37
group_1566		hypothetical protein	144	3	3	49	98	94	784	4.05 E-39	6.03E-35	2.15E-37
group_1539		hypothetical protein	144	3	3	49	98	94	784	4.05 E-39	6.03E-35	2.15E-37
group_1535		hypothetical protein	144	3	3	49	98	94	784	4.05 E-39	6.03E-35	2.15E-37
group_1534		hypothetical protein	144	3	3	49	98	94	784	4.05 E-39	6.03E-35	2.15E-37
group_1529		hypothetical protein	144	3	3	49	98	94	784	4.05 E-39	6.03E-35	2.15E-37
group_1525		hypothetical protein	144	3	3	49	98	94	784	4.05 E-39	6.03E-35	2.15E-37
group_10175		hypothetical protein	147	6	0	46	100	88	inf	5.70 E-39	8.48E-35	2.91E-37
ybiI_3	ybiI_3	putative protein YbiI	147	6	0	46	100	88	inf	5.70 E-39	8.48E-35	2.91E-37

group_8760	hypothetical protein	147	6	0	46	100	88	inf	5.70 E-39	8.48E-35	2.91E-37
group_871	hypothetical protein	147	6	0	46	100	88	inf	5.70 E-39	8.48E-35	2.91E-37
group_565	hypothetical protein	147	6	0	46	100	88	inf	5.70 E-39	8.48E-35	2.91E-37
group_448	hypothetical protein	147	6	0	46	100	88	inf	5.70 E-39	8.48E-35	2.91E-37
group_422	hypothetical protein	147	6	0	46	100	88	inf	5.70 E-39	8.48E-35	2.91E-37
group_257	hypothetical protein	147	6	0	46	100	88	inf	5.70 E-39	8.48E-35	2.91E-37
group_166	hypothetical protein	147	6	0	46	100	88	inf	5.70 E-39	8.48E-35	2.91E-37
group_105	hypothetical protein	147	6	0	46	100	88	inf	5.70 E-39	8.48E-35	2.91E-37
group_1635	hypothetical protein	146	5	1	47	99	90	##	1.04 E-38	1.54E-34	5.25E-37
group_1634	hypothetical protein	146	5	1	47	99	90	##	1.04 E-38	1.54E-34	5.25E-37
group_1484	hypothetical protein	146	5	1	47	99	90	##	1.04 E-38	1.54E-34	5.25E-37
group_10647	hypothetical protein	140	1	7	51	95	98	##	1.50 E-38	2.23E-34	7.44E-37
group_9740	hypothetical protein	140	1	7	51	95	98	##	1.50 E-38	2.23E-34	7.44E-37
group_8053	hypothetical protein	140	1	7	51	95	98	##	1.50 E-38	2.23E-34	7.44E-37
group_5423	hypothetical protein	140	1	7	51	95	98	##	1.50 E-38	2.23E-34	7.44E-37
group_5083	hypothetical protein	140	1	7	51	95	98	##	1.50 E-38	2.23E-34	7.44E-37

group_657		hypothetical protein	140	1	7	51	95	98	##	1.50 E-38	2.23E-34	7.44E-37
group_10074		hypothetical protein	137	0	10	52	93	100	inf	3.80 E-38	5.65E-34	1.87E-36
group_7517		hypothetical protein	137	0	10	52	93	100	inf	3.80 E-38	5.65E-34	1.87E-36
group_4794		hypothetical protein	143	3	4	49	97	94	584	5.27 E-38	7.83E-34	2.58E-36
group_1036		hypothetical protein	143	3	4	49	97	94	584	5.27 E-38	7.83E-34	2.58E-36
group_10746		hypothetical protein	141	2	6	50	96	96	588	1.17 E-37	1.74E-33	2.71E-36
group_10734		hypothetical protein	141	2	6	50	96	96	588	1.17 E-37	1.74E-33	2.71E-36
group_10675		hypothetical protein	141	2	6	50	96	96	588	1.17 E-37	1.74E-33	2.71E-36
group_10658		hypothetical protein	141	2	6	50	96	96	588	1.17 E-37	1.74E-33	2.71E-36
group_10641		hypothetical protein	141	2	6	50	96	96	588	1.17 E-37	1.74E-33	2.71E-36
group_10609		hypothetical protein	141	2	6	50	96	96	588	1.17 E-37	1.74E-33	2.71E-36
group_10600		hypothetical protein	141	2	6	50	96	96	588	1.17 E-37	1.74E-33	2.71E-36
spo0J~~~noc	spo0J;;noc	Stage 0 sporulation protein J;hypothetical protein;Nucleoid occlusion protein	141	2	6	50	96	96	588	1.17 E-37	1.74E-33	2.71E-36
group_10544		hypothetical protein	141	2	6	50	96	96	588	1.17 E-37	1.74E-33	2.71E-36

group_10538		hypothetical protein	141	2	6	50	96	96	588	1.17 E-37	1.74E-33	2.71E-36
group_10532		hypothetical protein	141	2	6	50	96	96	588	1.17 E-37	1.74E-33	2.71E-36
group_10487		hypothetical protein	141	2	6	50	96	96	588	1.17 E-37	1.74E-33	2.71E-36
rcsC_3~~~rcsC_1	rcsC_3;rcsC_1	Sensor histidine kinase RcsC	141	2	6	50	96	96	588	1.17 E-37	1.74E-33	2.71E-36
lig	;lig	hypothetical protein;DNA ligase	141	2	6	50	96	96	588	1.17 E-37	1.74E-33	2.71E-36
group_10434		hypothetical protein	141	2	6	50	96	96	588	1.17 E-37	1.74E-33	2.71E-36
group_10421		hypothetical protein	141	2	6	50	96	96	588	1.17 E-37	1.74E-33	2.71E-36
group_10417		hypothetical protein	141	2	6	50	96	96	588	1.17 E-37	1.74E-33	2.71E-36
group_10408		hypothetical protein	141	2	6	50	96	96	588	1.17 E-37	1.74E-33	2.71E-36
group_10402		hypothetical protein	141	2	6	50	96	96	588	1.17 E-37	1.74E-33	2.71E-36
atsB_2~~~atsB	atsB_2;atsB	Anaerobic sulfatase-maturating enzyme	141	2	6	50	96	96	588	1.17 E-37	1.74E-33	2.71E-36
atsB_1	;atsB_1	Anaerobic sulfatase-maturating enzyme;hypothetical protein	141	2	6	50	96	96	588	1.17 E-37	1.74E-33	2.71E-36

group_10387		hypothetical protein	141	2	6	50	96	96	588	1.17 E-37	1.74E-33	2.71E-36
cdaR_1~~~cdaR_2	cdaR_1;cdaR_2	Carbohydrate diacid regulator	141	2	6	50	96	96	588	1.17 E-37	1.74E-33	2.71E-36
group_10364		hypothetical protein	141	2	6	50	96	96	588	1.17 E-37	1.74E-33	2.71E-36
group_10359		hypothetical protein	141	2	6	50	96	96	588	1.17 E-37	1.74E-33	2.71E-36
group_10295		hypothetical protein	141	2	6	50	96	96	588	1.17 E-37	1.74E-33	2.71E-36
group_10294		hypothetical protein	141	2	6	50	96	96	588	1.17 E-37	1.74E-33	2.71E-36
group_10289		hypothetical protein	141	2	6	50	96	96	588	1.17 E-37	1.74E-33	2.71E-36
group_10218		hypothetical protein	141	2	6	50	96	96	588	1.17 E-37	1.74E-33	2.71E-36
group_10159		putative signaling protein	141	2	6	50	96	96	588	1.17 E-37	1.74E-33	2.71E-36
group_10101		hypothetical protein	141	2	6	50	96	96	588	1.17 E-37	1.74E-33	2.71E-36
group_10096		hypothetical protein	141	2	6	50	96	96	588	1.17 E-37	1.74E-33	2.71E-36
group_10094		hypothetical protein	141	2	6	50	96	96	588	1.17 E-37	1.74E-33	2.71E-36
group_10079	xerC_1	Tyrosine recombinase XerC	141	2	6	50	96	96	588	1.17 E-37	1.74E-33	2.71E-36
group_10064		hypothetical protein	141	2	6	50	96	96	588	1.17 E-37	1.74E-33	2.71E-36
group_9935		hypothetical protein	141	2	6	50	96	96	588	1.17 E-37	1.74E-33	2.71E-36

group_9929		hypothetical protein	141	2	6	50	96	96	588	1.17 E-37	1.74E-33	2.71E-36
yiaD_3~~~yiaD_1	yiaD_3;yiaD_1	putative lipoprotein YiaD	141	2	6	50	96	96	588	1.17 E-37	1.74E-33	2.71E-36
group_9892		hypothetical protein	141	2	6	50	96	96	588	1.17 E-37	1.74E-33	2.71E-36
group_9891		hypothetical protein	141	2	6	50	96	96	588	1.17 E-37	1.74E-33	2.71E-36
group_9888		hypothetical protein	141	2	6	50	96	96	588	1.17 E-37	1.74E-33	2.71E-36
group_9868		hypothetical protein	141	2	6	50	96	96	588	1.17 E-37	1.74E-33	2.71E-36
group_9861		hypothetical protein	141	2	6	50	96	96	588	1.17 E-37	1.74E-33	2.71E-36
group_9847		hypothetical protein	141	2	6	50	96	96	588	1.17 E-37	1.74E-33	2.71E-36
group_9845		hypothetical protein	141	2	6	50	96	96	588	1.17 E-37	1.74E-33	2.71E-36
group_9843		hypothetical protein	141	2	6	50	96	96	588	1.17 E-37	1.74E-33	2.71E-36
nadE_2	;nadE_2	hypothetical protein;NH(3)-dependent NAD(+) synthetase	141	2	6	50	96	96	588	1.17 E-37	1.74E-33	2.71E-36
group_9836		hypothetical protein	141	2	6	50	96	96	588	1.17 E-37	1.74E-33	2.71E-36
group_9801		hypothetical protein	141	2	6	50	96	96	588	1.17 E-37	1.74E-33	2.71E-36
group_9774		hypothetical protein	141	2	6	50	96	96	588	1.17 E-37	1.74E-33	2.71E-36

group_9756		hypothetical protein	141	2	6	50	96	96	588	1.17E-37	1.74E-33	2.71E-36
group_9732		hypothetical protein	141	2	6	50	96	96	588	1.17E-37	1.74E-33	2.71E-36
group_9720		hypothetical protein	141	2	6	50	96	96	588	1.17E-37	1.74E-33	2.71E-36
group_9719		hypothetical protein	141	2	6	50	96	96	588	1.17E-37	1.74E-33	2.71E-36
group_9695		hypothetical protein	141	2	6	50	96	96	588	1.17E-37	1.74E-33	2.71E-36
group_9681	;dnaG_2	hypothetical protein;DNA primase	141	2	6	50	96	96	588	1.17E-37	1.74E-33	2.71E-36
group_9633		hypothetical protein	141	2	6	50	96	96	588	1.17E-37	1.74E-33	2.71E-36
group_9621		hypothetical protein	141	2	6	50	96	96	588	1.17E-37	1.74E-33	2.71E-36
group_9613		hypothetical protein	141	2	6	50	96	96	588	1.17E-37	1.74E-33	2.71E-36
group_9606		hypothetical protein	141	2	6	50	96	96	588	1.17E-37	1.74E-33	2.71E-36
group_9600		hypothetical protein	141	2	6	50	96	96	588	1.17E-37	1.74E-33	2.71E-36
group_9557		hypothetical protein	141	2	6	50	96	96	588	1.17E-37	1.74E-33	2.71E-36
group_9525		hypothetical protein	141	2	6	50	96	96	588	1.17E-37	1.74E-33	2.71E-36
group_9468		hypothetical protein	141	2	6	50	96	96	588	1.17E-37	1.74E-33	2.71E-36
group_9414		hypothetical protein	141	2	6	50	96	96	588	1.17E-37	1.74E-33	2.71E-36
group_9382		hypothetical protein	141	2	6	50	96	96	588	1.17E-37	1.74E-33	2.71E-36

group_9370		hypothetical protein	141	2	6	50	96	96	588	1.17 E-37	1.74E-33	2.71E-36
group_9362		hypothetical protein	141	2	6	50	96	96	588	1.17 E-37	1.74E-33	2.71E-36
group_9313		hypothetical protein	141	2	6	50	96	96	588	1.17 E-37	1.74E-33	2.71E-36
group_9302		hypothetical protein	141	2	6	50	96	96	588	1.17 E-37	1.74E-33	2.71E-36
group_9275		hypothetical protein	141	2	6	50	96	96	588	1.17 E-37	1.74E-33	2.71E-36
rssB_3~~~cheB_3	rssB_3;cheB_3	Regulator of RpoS;Protein-glutamate methylesterase/protein-glutamine glutaminase	141	2	6	50	96	96	588	1.17 E-37	1.74E-33	2.71E-36
group_9242		hypothetical protein	141	2	6	50	96	96	588	1.17 E-37	1.74E-33	2.71E-36
group_9193		hypothetical protein	141	2	6	50	96	96	588	1.17 E-37	1.74E-33	2.71E-36
group_9043		hypothetical protein	141	2	6	50	96	96	588	1.17 E-37	1.74E-33	2.71E-36
group_8947	csrA1	Translational regulator CsrA1	141	2	6	50	96	96	588	1.17 E-37	1.74E-33	2.71E-36
group_8935		hypothetical protein	141	2	6	50	96	96	588	1.17 E-37	1.74E-33	2.71E-36
group_8907		hypothetical protein	141	2	6	50	96	96	588	1.17 E-37	1.74E-33	2.71E-36
group_8876		hypothetical protein	141	2	6	50	96	96	588	1.17 E-37	1.74E-33	2.71E-36

group_8861	hypothetical protein	141	2	6	50	96	96	588	1.17 E-37	1.74E-33	2.71E-36
group_8742	hypothetical protein	141	2	6	50	96	96	588	1.17 E-37	1.74E-33	2.71E-36
group_8588	hypothetical protein	141	2	6	50	96	96	588	1.17 E-37	1.74E-33	2.71E-36
group_8562	hypothetical protein	141	2	6	50	96	96	588	1.17 E-37	1.74E-33	2.71E-36
group_8536	hypothetical protein	141	2	6	50	96	96	588	1.17 E-37	1.74E-33	2.71E-36
group_8520	hypothetical protein	141	2	6	50	96	96	588	1.17 E-37	1.74E-33	2.71E-36
group_8414	hypothetical protein	141	2	6	50	96	96	588	1.17 E-37	1.74E-33	2.71E-36
group_8378	hypothetical protein	141	2	6	50	96	96	588	1.17 E-37	1.74E-33	2.71E-36
group_8377	hypothetical protein	141	2	6	50	96	96	588	1.17 E-37	1.74E-33	2.71E-36
group_8347	hypothetical protein	141	2	6	50	96	96	588	1.17 E-37	1.74E-33	2.71E-36
group_8345	hypothetical protein	141	2	6	50	96	96	588	1.17 E-37	1.74E-33	2.71E-36
group_8265	hypothetical protein	141	2	6	50	96	96	588	1.17 E-37	1.74E-33	2.71E-36
group_8167	hypothetical protein	141	2	6	50	96	96	588	1.17 E-37	1.74E-33	2.71E-36
group_8161	hypothetical protein	141	2	6	50	96	96	588	1.17 E-37	1.74E-33	2.71E-36
group_8128	hypothetical protein	141	2	6	50	96	96	588	1.17 E-37	1.74E-33	2.71E-36
group_8035	hypothetical protein	141	2	6	50	96	96	588	1.17 E-37	1.74E-33	2.71E-36

group_8015		hypothetical protein	141	2	6	50	96	96	588	1.17 E-37	1.74E-33	2.71E-36
group_7980		hypothetical protein	141	2	6	50	96	96	588	1.17 E-37	1.74E-33	2.71E-36
group_7936		hypothetical protein	141	2	6	50	96	96	588	1.17 E-37	1.74E-33	2.71E-36
group_7909		hypothetical protein	141	2	6	50	96	96	588	1.17 E-37	1.74E-33	2.71E-36
group_7851	hupB_2	DNA-binding protein HU-beta	141	2	6	50	96	96	588	1.17 E-37	1.74E-33	2.71E-36
group_7836		hypothetical protein	141	2	6	50	96	96	588	1.17 E-37	1.74E-33	2.71E-36
group_7786		hypothetical protein	141	2	6	50	96	96	588	1.17 E-37	1.74E-33	2.71E-36
group_7763		hypothetical protein	141	2	6	50	96	96	588	1.17 E-37	1.74E-33	2.71E-36
group_7762		hypothetical protein	141	2	6	50	96	96	588	1.17 E-37	1.74E-33	2.71E-36
group_7690		hypothetical protein	141	2	6	50	96	96	588	1.17 E-37	1.74E-33	2.71E-36
group_7626		hypothetical protein	141	2	6	50	96	96	588	1.17 E-37	1.74E-33	2.71E-36
group_7562		hypothetical protein	141	2	6	50	96	96	588	1.17 E-37	1.74E-33	2.71E-36
group_7561		hypothetical protein	141	2	6	50	96	96	588	1.17 E-37	1.74E-33	2.71E-36
group_7491		hypothetical protein	141	2	6	50	96	96	588	1.17 E-37	1.74E-33	2.71E-36
group_7429		hypothetical protein	141	2	6	50	96	96	588	1.17 E-37	1.74E-33	2.71E-36
group_7393		hypothetical protein	141	2	6	50	96	96	588	1.17 E-37	1.74E-33	2.71E-36

group_7392	hypothetical protein	141	2	6	50	96	96	588	1.17 E-37	1.74E-33	2.71E-36
group_7367	hypothetical protein	141	2	6	50	96	96	588	1.17 E-37	1.74E-33	2.71E-36
group_7345	hypothetical protein	141	2	6	50	96	96	588	1.17 E-37	1.74E-33	2.71E-36
group_7344	hypothetical protein	141	2	6	50	96	96	588	1.17 E-37	1.74E-33	2.71E-36
group_7308	hypothetical protein	141	2	6	50	96	96	588	1.17 E-37	1.74E-33	2.71E-36
group_7307	hypothetical protein	141	2	6	50	96	96	588	1.17 E-37	1.74E-33	2.71E-36
group_7281	hypothetical protein	141	2	6	50	96	96	588	1.17 E-37	1.74E-33	2.71E-36
group_7277	hypothetical protein	141	2	6	50	96	96	588	1.17 E-37	1.74E-33	2.71E-36
group_7256	hypothetical protein	141	2	6	50	96	96	588	1.17 E-37	1.74E-33	2.71E-36
group_7255	hypothetical protein	141	2	6	50	96	96	588	1.17 E-37	1.74E-33	2.71E-36
group_7225	hypothetical protein	141	2	6	50	96	96	588	1.17 E-37	1.74E-33	2.71E-36
group_7203	hypothetical protein	141	2	6	50	96	96	588	1.17 E-37	1.74E-33	2.71E-36
group_7182	hypothetical protein	141	2	6	50	96	96	588	1.17 E-37	1.74E-33	2.71E-36
group_7181	hypothetical protein	141	2	6	50	96	96	588	1.17 E-37	1.74E-33	2.71E-36
group_7180	hypothetical protein	141	2	6	50	96	96	588	1.17 E-37	1.74E-33	2.71E-36
group_7122	hypothetical protein	141	2	6	50	96	96	588	1.17 E-37	1.74E-33	2.71E-36

group_7121		hypothetical protein	141	2	6	50	96	96	588	1.17 E-37	1.74E-33	2.71E-36
group_7031		hypothetical protein	141	2	6	50	96	96	588	1.17 E-37	1.74E-33	2.71E-36
group_7000		hypothetical protein	141	2	6	50	96	96	588	1.17 E-37	1.74E-33	2.71E-36
group_6975		hypothetical protein	141	2	6	50	96	96	588	1.17 E-37	1.74E-33	2.71E-36
group_6933		hypothetical protein	141	2	6	50	96	96	588	1.17 E-37	1.74E-33	2.71E-36
group_6904		hypothetical protein	141	2	6	50	96	96	588	1.17 E-37	1.74E-33	2.71E-36
group_6859		hypothetical protein	141	2	6	50	96	96	588	1.17 E-37	1.74E-33	2.71E-36
group_6834		hypothetical protein	141	2	6	50	96	96	588	1.17 E-37	1.74E-33	2.71E-36
group_6833		hypothetical protein	141	2	6	50	96	96	588	1.17 E-37	1.74E-33	2.71E-36
group_6832		hypothetical protein	141	2	6	50	96	96	588	1.17 E-37	1.74E-33	2.71E-36
group_6824		hypothetical protein	141	2	6	50	96	96	588	1.17 E-37	1.74E-33	2.71E-36
group_6811		hypothetical protein	141	2	6	50	96	96	588	1.17 E-37	1.74E-33	2.71E-36
group_6810		hypothetical protein	141	2	6	50	96	96	588	1.17 E-37	1.74E-33	2.71E-36
walR_3	walR_3	Transcriptional regulatory protein WalR	141	2	6	50	96	96	588	1.17 E-37	1.74E-33	2.71E-36
group_6729		hypothetical protein	141	2	6	50	96	96	588	1.17 E-37	1.74E-33	2.71E-36
group_6713		hypothetical protein	141	2	6	50	96	96	588	1.17 E-37	1.74E-33	2.71E-36

group_6712		hypothetical protein	141	2	6	50	96	96	588	1.17 E-37	1.74E-33	2.71E-36
group_6711	walR_2	Transcriptional regulatory protein WalR	141	2	6	50	96	96	588	1.17 E-37	1.74E-33	2.71E-36
group_6704		hypothetical protein	141	2	6	50	96	96	588	1.17 E-37	1.74E-33	2.71E-36
group_6666		hypothetical protein	141	2	6	50	96	96	588	1.17 E-37	1.74E-33	2.71E-36
rodZ_2	rodZ_2	Cytoskeleton protein RodZ	141	2	6	50	96	96	588	1.17 E-37	1.74E-33	2.71E-36
group_6576		hypothetical protein	141	2	6	50	96	96	588	1.17 E-37	1.74E-33	2.71E-36
dksA_2	dksA_2	RNA polymerase-binding transcription factor DksA	141	2	6	50	96	96	588	1.17 E-37	1.74E-33	2.71E-36
group_6515		hypothetical protein	141	2	6	50	96	96	588	1.17 E-37	1.74E-33	2.71E-36
group_6465		hypothetical protein	141	2	6	50	96	96	588	1.17 E-37	1.74E-33	2.71E-36
group_6435		hypothetical protein	141	2	6	50	96	96	588	1.17 E-37	1.74E-33	2.71E-36
group_6397		hypothetical protein	141	2	6	50	96	96	588	1.17 E-37	1.74E-33	2.71E-36
group_6360		hypothetical protein	141	2	6	50	96	96	588	1.17 E-37	1.74E-33	2.71E-36
group_6359		hypothetical protein	141	2	6	50	96	96	588	1.17 E-37	1.74E-33	2.71E-36
group_6333		hypothetical protein	141	2	6	50	96	96	588	1.17 E-37	1.74E-33	2.71E-36

group_6332	hypothetical protein	141	2	6	50	96	96	588	1.17 E-37	1.74E-33	2.71E-36
group_6295	hypothetical protein	141	2	6	50	96	96	588	1.17 E-37	1.74E-33	2.71E-36
group_6268	hypothetical protein	141	2	6	50	96	96	588	1.17 E-37	1.74E-33	2.71E-36
group_6205	hypothetical protein	141	2	6	50	96	96	588	1.17 E-37	1.74E-33	2.71E-36
group_6203	hypothetical protein	141	2	6	50	96	96	588	1.17 E-37	1.74E-33	2.71E-36
group_6182	hypothetical protein	141	2	6	50	96	96	588	1.17 E-37	1.74E-33	2.71E-36
group_6129	hypothetical protein	141	2	6	50	96	96	588	1.17 E-37	1.74E-33	2.71E-36
group_6094	hypothetical protein	141	2	6	50	96	96	588	1.17 E-37	1.74E-33	2.71E-36
group_6073	hypothetical protein	141	2	6	50	96	96	588	1.17 E-37	1.74E-33	2.71E-36
group_6072	hypothetical protein	141	2	6	50	96	96	588	1.17 E-37	1.74E-33	2.71E-36
group_6044	hypothetical protein	141	2	6	50	96	96	588	1.17 E-37	1.74E-33	2.71E-36
group_5867	hypothetical protein	141	2	6	50	96	96	588	1.17 E-37	1.74E-33	2.71E-36
group_5793	hypothetical protein	141	2	6	50	96	96	588	1.17 E-37	1.74E-33	2.71E-36
group_5772	hypothetical protein	141	2	6	50	96	96	588	1.17 E-37	1.74E-33	2.71E-36
group_5761	hypothetical protein	141	2	6	50	96	96	588	1.17 E-37	1.74E-33	2.71E-36
group_5746	hypothetical protein	141	2	6	50	96	96	588	1.17 E-37	1.74E-33	2.71E-36

group_5719		hypothetical protein	141	2	6	50	96	96	588	1.17 E-37	1.74E-33	2.71E-36
group_5705		hypothetical protein	141	2	6	50	96	96	588	1.17 E-37	1.74E-33	2.71E-36
group_5696		hypothetical protein	141	2	6	50	96	96	588	1.17 E-37	1.74E-33	2.71E-36
group_5685		hypothetical protein	141	2	6	50	96	96	588	1.17 E-37	1.74E-33	2.71E-36
group_5663		hypothetical protein	141	2	6	50	96	96	588	1.17 E-37	1.74E-33	2.71E-36
group_5657		hypothetical protein	141	2	6	50	96	96	588	1.17 E-37	1.74E-33	2.71E-36
group_5639		hypothetical protein	141	2	6	50	96	96	588	1.17 E-37	1.74E-33	2.71E-36
group_5585		hypothetical protein	141	2	6	50	96	96	588	1.17 E-37	1.74E-33	2.71E-36
group_5565		hypothetical protein	141	2	6	50	96	96	588	1.17 E-37	1.74E-33	2.71E-36
sspA_3	sspA_3	Stringent starvation protein A	141	2	6	50	96	96	588	1.17 E-37	1.74E-33	2.71E-36
group_5410		hypothetical protein	141	2	6	50	96	96	588	1.17 E-37	1.74E-33	2.71E-36
group_5409		hypothetical protein	141	2	6	50	96	96	588	1.17 E-37	1.74E-33	2.71E-36
group_5375		hypothetical protein	141	2	6	50	96	96	588	1.17 E-37	1.74E-33	2.71E-36
vfr_2	vfr_2	Cyclic AMP receptor-like protein	141	2	6	50	96	96	588	1.17 E-37	1.74E-33	2.71E-36
group_5358		hypothetical protein	141	2	6	50	96	96	588	1.17 E-37	1.74E-33	2.71E-36

group_5331		hypothetical protein	141	2	6	50	96	96	588	1.17 E-37	1.74E-33	2.71E-36
group_5312		hypothetical protein	141	2	6	50	96	96	588	1.17 E-37	1.74E-33	2.71E-36
group_5294		hypothetical protein	141	2	6	50	96	96	588	1.17 E-37	1.74E-33	2.71E-36
group_5274		hypothetical protein	141	2	6	50	96	96	588	1.17 E-37	1.74E-33	2.71E-36
group_5273		hypothetical protein	141	2	6	50	96	96	588	1.17 E-37	1.74E-33	2.71E-36
group_5181		hypothetical protein	141	2	6	50	96	96	588	1.17 E-37	1.74E-33	2.71E-36
group_5164		hypothetical protein	141	2	6	50	96	96	588	1.17 E-37	1.74E-33	2.71E-36
group_5163		hypothetical protein	141	2	6	50	96	96	588	1.17 E-37	1.74E-33	2.71E-36
group_5162		hypothetical protein	141	2	6	50	96	96	588	1.17 E-37	1.74E-33	2.71E-36
group_5118		hypothetical protein	141	2	6	50	96	96	588	1.17 E-37	1.74E-33	2.71E-36
group_4978		hypothetical protein	141	2	6	50	96	96	588	1.17 E-37	1.74E-33	2.71E-36
sprT_1	sprT_1	Protein SprT	141	2	6	50	96	96	588	1.17 E-37	1.74E-33	2.71E-36
group_4943		hypothetical protein	141	2	6	50	96	96	588	1.17 E-37	1.74E-33	2.71E-36
group_4869		hypothetical protein	141	2	6	50	96	96	588	1.17 E-37	1.74E-33	2.71E-36
group_4867		hypothetical protein	141	2	6	50	96	96	588	1.17 E-37	1.74E-33	2.71E-36
group_4827		hypothetical protein	141	2	6	50	96	96	588	1.17 E-37	1.74E-33	2.71E-36

group_4798		hypothetical protein	141	2	6	50	96	96	588	1.17 E-37	1.74E-33	2.71E-36
group_4727		hypothetical protein	141	2	6	50	96	96	588	1.17 E-37	1.74E-33	2.71E-36
group_4524		hypothetical protein	141	2	6	50	96	96	588	1.17 E-37	1.74E-33	2.71E-36
group_4337		hypothetical protein	141	2	6	50	96	96	588	1.17 E-37	1.74E-33	2.71E-36
group_4287		hypothetical protein	141	2	6	50	96	96	588	1.17 E-37	1.74E-33	2.71E-36
group_4191		hypothetical protein	141	2	6	50	96	96	588	1.17 E-37	1.74E-33	2.71E-36
group_4189		hypothetical protein	141	2	6	50	96	96	588	1.17 E-37	1.74E-33	2.71E-36
group_4176		hypothetical protein	141	2	6	50	96	96	588	1.17 E-37	1.74E-33	2.71E-36
group_4107		hypothetical protein	141	2	6	50	96	96	588	1.17 E-37	1.74E-33	2.71E-36
group_4053		hypothetical protein	141	2	6	50	96	96	588	1.17 E-37	1.74E-33	2.71E-36
group_4050		hypothetical protein	141	2	6	50	96	96	588	1.17 E-37	1.74E-33	2.71E-36
group_4045		hypothetical protein	141	2	6	50	96	96	588	1.17 E-37	1.74E-33	2.71E-36
group_4033		hypothetical protein	141	2	6	50	96	96	588	1.17 E-37	1.74E-33	2.71E-36
group_4032		hypothetical protein	141	2	6	50	96	96	588	1.17 E-37	1.74E-33	2.71E-36
parB_1	parB_1	putative chromosome-partitioning protein ParB	141	2	6	50	96	96	588	1.17 E-37	1.74E-33	2.71E-36

group_3931		hypothetical protein	141	2	6	50	96	96	588	1.17 E-37	1.74E-33	2.71E-36
group_3853		hypothetical protein	141	2	6	50	96	96	588	1.17 E-37	1.74E-33	2.71E-36
group_3835		hypothetical protein	141	2	6	50	96	96	588	1.17 E-37	1.74E-33	2.71E-36
soj_3	soj_3	Sporulation initiation inhibitor protein Soj	141	2	6	50	96	96	588	1.17 E-37	1.74E-33	2.71E-36
group_3804		hypothetical protein	141	2	6	50	96	96	588	1.17 E-37	1.74E-33	2.71E-36
group_3793		hypothetical protein	141	2	6	50	96	96	588	1.17 E-37	1.74E-33	2.71E-36
group_3780		hypothetical protein	141	2	6	50	96	96	588	1.17 E-37	1.74E-33	2.71E-36
group_3773		hypothetical protein	141	2	6	50	96	96	588	1.17 E-37	1.74E-33	2.71E-36
group_3716		hypothetical protein	141	2	6	50	96	96	588	1.17 E-37	1.74E-33	2.71E-36
group_3715	rdgC_1	Recombination-associated protein RdgC	141	2	6	50	96	96	588	1.17 E-37	1.74E-33	2.71E-36
group_3677		hypothetical protein	141	2	6	50	96	96	588	1.17 E-37	1.74E-33	2.71E-36
group_3669		hypothetical protein	141	2	6	50	96	96	588	1.17 E-37	1.74E-33	2.71E-36
cheB1_2	cheB1_2	Protein-glutamate methylesterase/protein-glutamine glutaminase 1	141	2	6	50	96	96	588	1.17 E-37	1.74E-33	2.71E-36

group_3648		hypothetical protein	141	2	6	50	96	96	588	1.17 E-37	1.74E-33	2.71E-36
group_3581		hypothetical protein	141	2	6	50	96	96	588	1.17 E-37	1.74E-33	2.71E-36
group_3494		hypothetical protein	141	2	6	50	96	96	588	1.17 E-37	1.74E-33	2.71E-36
group_3373		hypothetical protein	141	2	6	50	96	96	588	1.17 E-37	1.74E-33	2.71E-36
group_3278	yejK_2	Nucleoid-associated protein YejK	141	2	6	50	96	96	588	1.17 E-37	1.74E-33	2.71E-36
group_3277	pilT_3	Twitching mobility protein	141	2	6	50	96	96	588	1.17 E-37	1.74E-33	2.71E-36
group_3230		hypothetical protein	141	2	6	50	96	96	588	1.17 E-37	1.74E-33	2.71E-36
group_3224		hypothetical protein	141	2	6	50	96	96	588	1.17 E-37	1.74E-33	2.71E-36
recA_2	recA_2	Protein RecA	141	2	6	50	96	96	588	1.17 E-37	1.74E-33	2.71E-36
group_3116		hypothetical protein	141	2	6	50	96	96	588	1.17 E-37	1.74E-33	2.71E-36
group_3065		hypothetical protein	141	2	6	50	96	96	588	1.17 E-37	1.74E-33	2.71E-36
group_3036		hypothetical protein	141	2	6	50	96	96	588	1.17 E-37	1.74E-33	2.71E-36
group_3024		hypothetical protein	141	2	6	50	96	96	588	1.17 E-37	1.74E-33	2.71E-36
group_2976		hypothetical protein	141	2	6	50	96	96	588	1.17 E-37	1.74E-33	2.71E-36
group_2941		hypothetical protein	141	2	6	50	96	96	588	1.17 E-37	1.74E-33	2.71E-36

dnaN_1	dnaN_1	Beta sliding clamp	141	2	6	50	96	96	588	1.17 E-37	1.74E-33	2.71E-36
group_2898	recD2	ATP-dependent RecD-like DNA helicase	141	2	6	50	96	96	588	1.17 E-37	1.74E-33	2.71E-36
group_2890		hypothetical protein	141	2	6	50	96	96	588	1.17 E-37	1.74E-33	2.71E-36
cobS_2	cobS_2	Aerobic cobaltochelatase subunit CobS	141	2	6	50	96	96	588	1.17 E-37	1.74E-33	2.71E-36
group_2868		hypothetical protein	141	2	6	50	96	96	588	1.17 E-37	1.74E-33	2.71E-36
group_2848		hypothetical protein	141	2	6	50	96	96	588	1.17 E-37	1.74E-33	2.71E-36
group_2827		hypothetical protein	141	2	6	50	96	96	588	1.17 E-37	1.74E-33	2.71E-36
group_2816		Anaerobic sulfatase-maturating enzyme	141	2	6	50	96	96	588	1.17 E-37	1.74E-33	2.71E-36
group_2808	garK_2	Glycerate 2-kinase	141	2	6	50	96	96	588	1.17 E-37	1.74E-33	2.71E-36
group_2756		hypothetical protein	141	2	6	50	96	96	588	1.17 E-37	1.74E-33	2.71E-36
group_2724		hypothetical protein	141	2	6	50	96	96	588	1.17 E-37	1.74E-33	2.71E-36
group_2718		hypothetical protein	141	2	6	50	96	96	588	1.17 E-37	1.74E-33	2.71E-36
group_2706		hypothetical protein	141	2	6	50	96	96	588	1.17 E-37	1.74E-33	2.71E-36

group_2689		hypothetical protein	141	2	6	50	96	96	588	1.17 E-37	1.74E-33	2.71E-36
group_2680		hypothetical protein	141	2	6	50	96	96	588	1.17 E-37	1.74E-33	2.71E-36
korB	korB	Transcriptional repressor protein KorB	141	2	6	50	96	96	588	1.17 E-37	1.74E-33	2.71E-36
group_2599		hypothetical protein	141	2	6	50	96	96	588	1.17 E-37	1.74E-33	2.71E-36
group_2544		hypothetical protein	141	2	6	50	96	96	588	1.17 E-37	1.74E-33	2.71E-36
group_2535		hypothetical protein	141	2	6	50	96	96	588	1.17 E-37	1.74E-33	2.71E-36
group_2530		hypothetical protein	141	2	6	50	96	96	588	1.17 E-37	1.74E-33	2.71E-36
group_2512		hypothetical protein	141	2	6	50	96	96	588	1.17 E-37	1.74E-33	2.71E-36
group_2500		hypothetical protein	141	2	6	50	96	96	588	1.17 E-37	1.74E-33	2.71E-36
group_2491		hypothetical protein	141	2	6	50	96	96	588	1.17 E-37	1.74E-33	2.71E-36
group_2465		hypothetical protein	141	2	6	50	96	96	588	1.17 E-37	1.74E-33	2.71E-36
group_2405		hypothetical protein	141	2	6	50	96	96	588	1.17 E-37	1.74E-33	2.71E-36
group_2216		hypothetical protein	141	2	6	50	96	96	588	1.17 E-37	1.74E-33	2.71E-36
group_2186		hypothetical protein	141	2	6	50	96	96	588	1.17 E-37	1.74E-33	2.71E-36
sbcB_2	sbcB_2	Exodeoxyribonuclease I	141	2	6	50	96	96	588	1.17 E-37	1.74E-33	2.71E-36
group_2132		hypothetical protein	141	2	6	50	96	96	588	1.17 E-37	1.74E-33	2.71E-36

group_2119		hypothetical protein	141	2	6	50	96	96	588	1.17 E-37	1.74E-33	2.71E-36
group_2015		hypothetical protein	141	2	6	50	96	96	588	1.17 E-37	1.74E-33	2.71E-36
group_2002		hypothetical protein	141	2	6	50	96	96	588	1.17 E-37	1.74E-33	2.71E-36
group_1974		hypothetical protein	141	2	6	50	96	96	588	1.17 E-37	1.74E-33	2.71E-36
rpoS_2	rpoS_2	RNA polymerase sigma factor RpoS	141	2	6	50	96	96	588	1.17 E-37	1.74E-33	2.71E-36
group_1858		hypothetical protein	141	2	6	50	96	96	588	1.17 E-37	1.74E-33	2.71E-36
group_1788		hypothetical protein	141	2	6	50	96	96	588	1.17 E-37	1.74E-33	2.71E-36
group_1768		hypothetical protein	141	2	6	50	96	96	588	1.17 E-37	1.74E-33	2.71E-36
gspE	gspE	Putative type II secretion system protein E	141	2	6	50	96	96	588	1.17 E-37	1.74E-33	2.71E-36
ftsH2	ftsH2	ATP-dependent zinc metalloprotease FtsH 2	141	2	6	50	96	96	588	1.17 E-37	1.74E-33	2.71E-36
group_1219		hypothetical protein	141	2	6	50	96	96	588	1.17 E-37	1.74E-33	2.71E-36
group_1156		hypothetical protein	141	2	6	50	96	96	588	1.17 E-37	1.74E-33	2.71E-36
group_1097		hypothetical protein	141	2	6	50	96	96	588	1.17 E-37	1.74E-33	2.71E-36

group_1016		hypothetical protein	141	2	6	50	96	96	588	1.17 E-37	1.74E-33	2.71E-36
group_1015		hypothetical protein	141	2	6	50	96	96	588	1.17 E-37	1.74E-33	2.71E-36
group_1012		hypothetical protein	141	2	6	50	96	96	588	1.17 E-37	1.74E-33	2.71E-36
group_1010		hypothetical protein	141	2	6	50	96	96	588	1.17 E-37	1.74E-33	2.71E-36
group_999		hypothetical protein	141	2	6	50	96	96	588	1.17 E-37	1.74E-33	2.71E-36
group_982		hypothetical protein	141	2	6	50	96	96	588	1.17 E-37	1.74E-33	2.71E-36
orn_2	orn_2	Oligoribonuclease	141	2	6	50	96	96	588	1.17 E-37	1.74E-33	2.71E-36
group_980		hypothetical protein	141	2	6	50	96	96	588	1.17 E-37	1.74E-33	2.71E-36
group_925		hypothetical protein	141	2	6	50	96	96	588	1.17 E-37	1.74E-33	2.71E-36
group_915		hypothetical protein	141	2	6	50	96	96	588	1.17 E-37	1.74E-33	2.71E-36
group_850		hypothetical protein	141	2	6	50	96	96	588	1.17 E-37	1.74E-33	2.71E-36
kdpE_2~~~kdpE_1	kdpE_2;kdpE_1	KDP operon transcriptional regulatory protein KdpE	141	2	6	50	96	96	588	1.17 E-37	1.74E-33	2.71E-36
group_839		hypothetical protein	141	2	6	50	96	96	588	1.17 E-37	1.74E-33	2.71E-36
group_821		hypothetical protein	141	2	6	50	96	96	588	1.17 E-37	1.74E-33	2.71E-36
group_801		hypothetical protein	141	2	6	50	96	96	588	1.17 E-37	1.74E-33	2.71E-36

group_765		hypothetical protein	141	2	6	50	96	96	588	1.17E-37	1.74E-33	2.71E-36
group_739		hypothetical protein	141	2	6	50	96	96	588	1.17E-37	1.74E-33	2.71E-36
group_738		hypothetical protein	141	2	6	50	96	96	588	1.17E-37	1.74E-33	2.71E-36
group_708		hypothetical protein	141	2	6	50	96	96	588	1.17E-37	1.74E-33	2.71E-36
group_698		hypothetical protein	141	2	6	50	96	96	588	1.17E-37	1.74E-33	2.71E-36
group_685		hypothetical protein	141	2	6	50	96	96	588	1.17E-37	1.74E-33	2.71E-36
smc_1~~~smc_3	smc_1;;smc_3	Chromosome partition protein Smc;hypothetical protein	141	2	6	50	96	96	588	1.17E-37	1.74E-33	2.71E-36
group_614		hypothetical protein	141	2	6	50	96	96	588	1.17E-37	1.74E-33	2.71E-36
group_611		hypothetical protein	141	2	6	50	96	96	588	1.17E-37	1.74E-33	2.71E-36
uvrC_2~~~uvrC_1	uvrC_2;uvrC_1	UvrABC system protein C	141	2	6	50	96	96	588	1.17E-37	1.74E-33	2.71E-36
group_512		hypothetical protein	141	2	6	50	96	96	588	1.17E-37	1.74E-33	2.71E-36
outO_1~~~outO_2	outO_1;outO_2	Type 4 prepilin-like proteins leader peptide-processing enzyme	141	2	6	50	96	96	588	1.17E-37	1.74E-33	2.71E-36

group_493		hypothetical protein	141	2	6	50	96	96	588	1.17 E-37	1.74E-33	2.71E-36
group_453		hypothetical protein	141	2	6	50	96	96	588	1.17 E-37	1.74E-33	2.71E-36
group_443		hypothetical protein	141	2	6	50	96	96	588	1.17 E-37	1.74E-33	2.71E-36
group_441		hypothetical protein	141	2	6	50	96	96	588	1.17 E-37	1.74E-33	2.71E-36
group_435		hypothetical protein	141	2	6	50	96	96	588	1.17 E-37	1.74E-33	2.71E-36
group_378		hypothetical protein	141	2	6	50	96	96	588	1.17 E-37	1.74E-33	2.71E-36
group_351		hypothetical protein	141	2	6	50	96	96	588	1.17 E-37	1.74E-33	2.71E-36
group_245		hypothetical protein	141	2	6	50	96	96	588	1.17 E-37	1.74E-33	2.71E-36
group_244		hypothetical protein	141	2	6	50	96	96	588	1.17 E-37	1.74E-33	2.71E-36
group_234	recB_1	RecBCD enzyme subunit RecB	141	2	6	50	96	96	588	1.17 E-37	1.74E-33	2.71E-36
group_217		hypothetical protein	141	2	6	50	96	96	588	1.17 E-37	1.74E-33	2.71E-36
group_205		Deoxyribonuclease	141	2	6	50	96	96	588	1.17 E-37	1.74E-33	2.71E-36
group_181		hypothetical protein	141	2	6	50	96	96	588	1.17 E-37	1.74E-33	2.71E-36
group_132		hypothetical protein	141	2	6	50	96	96	588	1.17 E-37	1.74E-33	2.71E-36
group_123		hypothetical protein	141	2	6	50	96	96	588	1.17 E-37	1.74E-33	2.71E-36
group_101		hypothetical protein	141	2	6	50	96	96	588	1.17 E-37	1.74E-33	2.71E-36

group_89		hypothetical protein	141	2	6	50	96	96	588	1.17 E-37	1.74E-33	2.71E-36
group_54		hypothetical protein	141	2	6	50	96	96	588	1.17 E-37	1.74E-33	2.71E-36
group_46		hypothetical protein	141	2	6	50	96	96	588	1.17 E-37	1.74E-33	2.71E-36
group_8		hypothetical protein	141	2	6	50	96	96	588	1.17 E-37	1.74E-33	2.71E-36
group_7		hypothetical protein	141	2	6	50	96	96	588	1.17 E-37	1.74E-33	2.71E-36
group_10395		hypothetical protein	147	7	0	45	100	87	inf	1.25 E-37	1.87E-33	2.87E-36
group_6414		hypothetical protein	147	7	0	45	100	87	inf	1.25 E-37	1.87E-33	2.87E-36
group_6246		hypothetical protein	147	7	0	45	100	87	inf	1.25 E-37	1.87E-33	2.87E-36
yxbC	yxbC	putative protein YxbC	147	7	0	45	100	87	inf	1.25 E-37	1.87E-33	2.87E-36
group_2913		hypothetical protein	147	7	0	45	100	87	inf	1.25 E-37	1.87E-33	2.87E-36
group_2681		hypothetical protein	147	7	0	45	100	87	inf	1.25 E-37	1.87E-33	2.87E-36
group_1029		hypothetical protein	147	7	0	45	100	87	inf	1.25 E-37	1.87E-33	2.87E-36
group_9983		IS3 family transposase ISPa57	0	45	147	7	0	13	0	1.25 E-37	1.87E-33	2.87E-36
group_4808		hypothetical protein	144	4	3	48	98	92	576	1.41 E-37	2.10E-33	3.21E-36
group_1593		hypothetical protein	144	4	3	48	98	92	576	1.41 E-37	2.10E-33	3.21E-36
group_1545		hypothetical protein	144	4	3	48	98	92	576	1.41 E-37	2.10E-33	3.21E-36

group_1544		hypothetical protein	144	4	3	48	98	92	576	1.41 E-37	2.10E-33	3.21E-36
group_1542		hypothetical protein	144	4	3	48	98	92	576	1.41 E-37	2.10E-33	3.21E-36
group_1522		hypothetical protein	144	4	3	48	98	92	576	1.41 E-37	2.10E-33	3.21E-36
group_289		hypothetical protein	144	4	3	48	98	92	576	1.41 E-37	2.10E-33	3.21E-36
group_5496		hypothetical protein	136	0	11	52	93	100	inf	2.18 E-37	3.24E-33	4.92E-36
leuE_3	leuE_3	Leucine efflux protein	136	0	11	52	93	100	inf	2.18 E-37	3.24E-33	4.92E-36
group_852		hypothetical protein	142	3	5	49	97	94	464	5.57 E-37	8.29E-33	1.26E-35
group_9867	;recB_1	hypothetical protein;RecBCD enzyme subunit RecB	138	1	9	51	94	98	782	7.27 E-37	1.08E-32	1.63E-35
group_6251		hypothetical protein	138	1	9	51	94	98	782	7.27 E-37	1.08E-32	1.63E-35
group_1895	recF_4	DNA replication and repair protein RecF	138	1	9	51	94	98	782	7.27 E-37	1.08E-32	1.63E-35
group_9850		hypothetical protein	140	2	7	50	95	96	500	9.37 E-37	1.39E-32	2.09E-35
group_9007		hypothetical protein	140	2	7	50	95	96	500	9.37 E-37	1.39E-32	2.09E-35
group_1040		hypothetical protein	140	2	7	50	95	96	500	9.37 E-37	1.39E-32	2.09E-35
group_409		hypothetical protein	140	2	7	50	95	96	500	9.37 E-37	1.39E-32	2.09E-35

parE1~~~parE1_1~~~parE1_2	parE1;parE1_1;parE1_2	Toxin ParE1	143	4	4	48	97	92	429	1.79E-36	2.66E-32	3.99E-35
group_9056		hypothetical protein	137	1	10	51	93	98	699	4.40E-36	6.55E-32	9.78E-35
group_8456		hypothetical protein	137	1	10	51	93	98	699	4.40E-36	6.55E-32	9.78E-35
parE1	parE1	Toxin ParE1	137	1	10	51	93	98	699	4.40E-36	6.55E-32	9.78E-35
group_9852		hypothetical protein	141	3	6	49	96	94	384	5.00E-36	7.45E-32	1.10E-34
group_2602		hypothetical protein	141	3	6	49	96	94	384	5.00E-36	7.45E-32	1.10E-34
group_2550		hypothetical protein	141	3	6	49	96	94	384	5.00E-36	7.45E-32	1.10E-34
group_2134		hypothetical protein	141	3	6	49	96	94	384	5.00E-36	7.45E-32	1.10E-34
group_936		hypothetical protein	141	3	6	49	96	94	384	5.00E-36	7.45E-32	1.10E-34
group_534		hypothetical protein	141	3	6	49	96	94	384	5.00E-36	7.45E-32	1.10E-34
addA_1	addA_1	ATP-dependent helicase/nuclease subunit A	141	3	6	49	96	94	384	5.00E-36	7.45E-32	1.10E-34
group_152		hypothetical protein	141	3	6	49	96	94	384	5.00E-36	7.45E-32	1.10E-34
recD2_2	recD2_2	ATP-dependent RecD-like DNA helicase	141	3	6	49	96	94	384	5.00E-36	7.45E-32	1.10E-34
group_8824		hypothetical protein	140	3	7	49	95	94	327	3.92E-35	5.83E-31	8.58E-34

group_5182		Pentapeptide repeat protein	138	2	9	50	94	96	383	4.33 E-35	6.44E-31	9.46E-34
group_10032		hypothetical protein	143	5	4	47	97	90	336	4.89 E-35	7.28E-31	1.06E-33
group_4054		hypothetical protein	143	5	4	47	97	90	336	4.89 E-35	7.28E-31	1.06E-33
group_663		hypothetical protein	143	5	4	47	97	90	336	4.89 E-35	7.28E-31	1.06E-33
group_1533		hypothetical protein	144	6	3	46	98	88	368	9.33 E-35	1.39E-30	2.03E-33
group_1379		hypothetical protein	145	7	2	45	99	87	466	1.24 E-34	1.84E-30	2.68E-33
group_3535		hypothetical protein	135	1	12	51	92	98	574	1.28 E-34	1.91E-30	2.78E-33
group_7764		hypothetical protein	137	2	10	50	93	96	343	2.56 E-34	3.81E-30	5.53E-33
group_1654		IS110 family transposase ISPa62	137	2	10	50	93	96	343	2.56 E-34	3.81E-30	5.53E-33
group_10441		hypothetical protein	147	10	0	42	100	81	inf	6.61 E-34	9.84E-30	1.38E-32
group_10275	clpP_3;clpP_2	ATP-dependent Clp protease proteolytic subunit	147	10	0	42	100	81	inf	6.61 E-34	9.84E-30	1.38E-32
group_9629		hypothetical protein	147	10	0	42	100	81	inf	6.61 E-34	9.84E-30	1.38E-32
group_9475	rfbX	Putative O-antigen transporter	147	10	0	42	100	81	inf	6.61 E-34	9.84E-30	1.38E-32
bglA_2~~~bglA~~~bglA_1	bglA_2;bglA;bglA_1	Beta-glucosidase A	147	10	0	42	100	81	inf	6.61 E-34	9.84E-30	1.38E-32

capD_2~~~capD_1~~~capD	capD_2;capD_1;capD	UDP-glucose 4-epimerase	147	10	0	42	100	81	inf	6.61E-34	9.84E-30	1.38E-32
group_9352		hypothetical protein	147	10	0	42	100	81	inf	6.61E-34	9.84E-30	1.38E-32
wzzE~~~wzzB	;wzzE;wzzB	hypothetical protein;ECA polysaccharide chain length modulation protein;Chain length determinant protein	147	10	0	42	100	81	inf	6.61E-34	9.84E-30	1.38E-32
group_9153		hypothetical protein	147	10	0	42	100	81	inf	6.61E-34	9.84E-30	1.38E-32
group_9003		hypothetical protein	147	10	0	42	100	81	inf	6.61E-34	9.84E-30	1.38E-32
group_8392		hypothetical protein	147	10	0	42	100	81	inf	6.61E-34	9.84E-30	1.38E-32
group_8376		hypothetical protein	147	10	0	42	100	81	inf	6.61E-34	9.84E-30	1.38E-32
group_3611	gnu	N-acetyl-alpha-D-glucosaminyl-diphospho-ditransoctacis-undecaprenol 4-epimerase	147	10	0	42	100	81	inf	6.61E-34	9.84E-30	1.38E-32
group_3358		hypothetical protein	147	10	0	42	100	81	inf	6.61E-34	9.84E-30	1.38E-32
kfoC	kfoC	Chondroitin synthase	147	10	0	42	100	81	inf	6.61E-34	9.84E-30	1.38E-32

group_3220	tagO	putative undecaprenyl -phosphate N-acetylglucosaminyl 1-phosphate transferase	147	10	0	42	100	81	inf	6.61 E-34	9.84E-30	1.38E-32
wbjC	wbjC	UDP-2-acetamido-26-beta-L-arabino-hexul-4-ose reductase	147	10	0	42	100	81	inf	6.61 E-34	9.84E-30	1.38E-32
wbpI	wbpI	UDP-23-diacetamido-23-dideoxy-D-glucuronate 2-epimerase	147	10	0	42	100	81	inf	6.61 E-34	9.84E-30	1.38E-32
group_2537		hypothetical protein	147	10	0	42	100	81	inf	6.61 E-34	9.84E-30	1.38E-32
group_2066		hypothetical protein	147	10	0	42	100	81	inf	6.61 E-34	9.84E-30	1.38E-32
hel	hel	Lipoprotein E	147	10	0	42	100	81	inf	6.61 E-34	9.84E-30	1.38E-32
group_308		hypothetical protein	147	10	0	42	100	81	inf	6.61 E-34	9.84E-30	1.38E-32
group_9445		hypothetical protein	0	42	147	10	0	19	0	6.61 E-34	9.84E-30	1.38E-32
group_8999		hypothetical protein	0	42	147	10	0	19	0	6.61 E-34	9.84E-30	1.38E-32

group_3221		hypothetical protein	0	42	147	10	0	19	0	6.61 E-34	9.84E-30	1.38E-32
group_1054		hypothetical protein	143	6	4	46	97	88	274	1.12 E-33	1.67E-29	2.33E-32
vgrG1_4~~~vgrG1_7	vgrG1_4;vgrG1_7	Actin cross-linking toxin VgrG1	143	6	4	46	97	88	274	1.12 E-33	1.67E-29	2.33E-32
group_482		hypothetical protein	143	6	4	46	97	88	274	1.12 E-33	1.67E-29	2.33E-32
group_10315		hypothetical protein	140	4	7	48	95	92	240	1.24 E-33	1.84E-29	2.56E-32
group_7419		hypothetical protein	146	9	1	43	99	83	698	1.75 E-33	2.60E-29	3.61E-32
group_1455		IS3 family transposase ISPa32	146	9	1	43	99	83	698	1.75 E-33	2.60E-29	3.61E-32
group_10124	;umuD	hypothetical protein;Protein UmuD	141	5	6	47	96	90	221	4.20 E-33	6.25E-29	8.65E-32
yjiK_4	yjiK_4	putative protein YjiK	141	5	6	47	96	90	221	4.20 E-33	6.25E-29	8.65E-32
group_4508		IS6 family transposase IS6100	141	5	6	47	96	90	221	4.20 E-33	6.25E-29	8.65E-32
group_10046		hypothetical protein	129	0	18	52	88	100	inf	8.20 E-33	1.22E-28	1.68E-31
group_9315		hypothetical protein	147	11	0	41	100	79	inf	9.50 E-33	1.41E-28	1.94E-31
group_5055		hypothetical protein	147	11	0	41	100	79	inf	9.50 E-33	1.41E-28	1.94E-31
group_10257		hypothetical protein	0	41	147	11	0	21	0	9.50 E-33	1.41E-28	1.94E-31

group_10231		hypothetical protein	0	41	147	11	0	21	0	9.50E-33	1.41E-28	1.94E-31
ureI	ureI	Acid-activated urea channel	0	41	147	11	0	21	0	9.50E-33	1.41E-28	1.94E-31
group_4797	fabG_14	3-oxoacyl-[acyl-carrier-protein] reductase FabG	0	41	147	11	0	21	0	9.50E-33	1.41E-28	1.94E-31
group_592		hypothetical protein	143	7	4	45	97	87	230	2.21E-32	3.29E-28	4.50E-31
yedK_2~~~yedK_3	yedK_2;yedK_3	SOS response-associated protein YedK	140	5	7	47	95	90	188	3.13E-32	4.66E-28	6.37E-31

Tab. S6: Genome-wide association study (GWAS) identified genes associated with ST316 ear swab isolates.

References:

1. Waine DJ, Honeybourne D, Smith EG, Whitehouse JL, Dowson CG. 2009. Cross-sectional and longitudinal multilocus sequence typing of pseudomonas aeruginosa in cystic fibrosis sputum samples. J Clin Microbiol 47:3444-8.
2. Treepong P, Kos VN, Guyeux C, Blanc DS, Bertrand X, Valot B, Hocquet D. 2018. Global emergence of the widespread Pseudomonas aeruginosa ST235 clone. Clin Microbiol Infect 24:258-266.
3. Darling AC, Mau B, Blattner FR, Perna NT. 2004. Mauve: multiple alignment of conserved genomic sequence with rearrangements. Genome Res 14:1394-403.
4. Yang F, Liu C, Ji J, Cao W, Ding B, Xu X. 2021. Molecular Characteristics, Antimicrobial Resistance, and Biofilm Formation of Pseudomonas aeruginosa Isolated from Patients with Aural Infections in Shanghai, China. Infect Drug Resist 14:3637-3645.
5. Tonkin-Hill G, MacAlasdair N, Ruis C, Weimann A, Horesh G, Lees JA, Gladstone RA, Lo S, Beaudoin C, Floto RA, Frost SDW, Corander J, Bentley SD, Parkhill J. 2020. Producing polished prokaryotic pangenomes with the Panaroo pipeline. Genome Biol 21:180.
